# Human germline heterozygous gain-of-function *STAT6* variants cause severe allergic disease

**DOI:** 10.1084/jem.20221755

**Published:** 2023-03-08

**Authors:** Mehul Sharma, Daniel Leung, Mana Momenilandi, Lauren C.W. Jones, Lucia Pacillo, Alyssa E. James, Jill R. Murrell, Selket Delafontaine, Jesmeen Maimaris, Maryam Vaseghi-Shanjani, Kate L. Del Bel, Henry Y. Lu, Gilbert T. Chua, Silvia Di Cesare, Oriol Fornes, Zhongyi Liu, Gigliola Di Matteo, Maggie P. Fu, Donato Amodio, Issan Yee San Tam, Gavin Shueng Wai Chan, Ashish A. Sharma, Joshua Dalmann, Robin van der Lee, Géraldine Blanchard-Rohner, Susan Lin, Quentin Philippot, Phillip A. Richmond, Jessica J. Lee, Allison Matthews, Michael Seear, Alexandra K. Turvey, Rachael L. Philips, Terri F. Brown-Whitehorn, Christopher J. Gray, Kosuke Izumi, James R. Treat, Kathleen H. Wood, Justin Lack, Asya Khleborodova, Julie E. Niemela, Xingtian Yang, Rui Liang, Lin Kui, Christina Sze Man Wong, Grace Wing Kit Poon, Alexander Hoischen, Caspar I. van der Made, Jing Yang, Koon Wing Chan, Jaime Sou Da Rosa Duque, Pamela Pui Wah Lee, Marco Hok Kung Ho, Brian Hon Yin Chung, Huong Thi Minh Le, Wanling Yang, Pejman Rohani, Ali Fouladvand, Hassan Rokni-Zadeh, Majid Changi-Ashtiani, Mohammad Miryounesi, Anne Puel, Mohammad Shahrooei, Andrea Finocchi, Paolo Rossi, Beatrice Rivalta, Cristina Cifaldi, Antonio Novelli, Chiara Passarelli, Stefania Arasi, Dominique Bullens, Kate Sauer, Tania Claeys, Catherine M. Biggs, Emma C. Morris, Sergio D. Rosenzweig, John J. O’Shea, Wyeth W. Wasserman, H. Melanie Bedford, Clara D.M. van Karnebeek, Paolo Palma, Siobhan O. Burns, Isabelle Meyts, Jean-Laurent Casanova, Jonathan J. Lyons, Nima Parvaneh, Anh Thi Van Nguyen, Caterina Cancrini, Jennifer Heimall, Hanan Ahmed, Margaret L. McKinnon, Yu Lung Lau, Vivien Béziat, Stuart E. Turvey

**Affiliations:** 1 Dept. of Pediatrics, BC Children’s Hospital, University of British Columbia, Vancouver, Canada; 2 Dept. of Paediatrics and Adolescent Medicine, School of Clinical Medicine, The University of Hong Kong, Hong Kong, China; 3 Laboratory of Human Genetics of Infectious Diseases, Necker Branch, INSERM, Necker Hospital for Sick Children, Paris, France; 4 Imagine Institute, University of Paris-Cité, Paris, France; 5 Dept. of System Medicine, Pediatric Chair, University of Tor Vergata, Rome, Italy; 6 Academic Dept. of Pediatrics (DPUO), Unit of Clinical Immunology and Vaccinology, IRCCS Bambin Gesù Children Hospital, Rome, Italy; 7 Research Unit of Primary Immunodeficiency, IRCCS Bambin Gesù Children Hospital, Rome, Italy; 8 Translational Allergic Immunopathology Unit, Laboratory of Allergic Diseases, National Institute of Allergy and Infectious Diseases (NIAID), National Institutes of Health (NIH), Bethesda, MD, USA; 9 Pathology and Laboratory Medicine, Division of Genomic Diagnostics, Children’s Hospital of Philadelphia, Philadelphia, PA, USA; 10 Dept. of Microbiology, Immunology and Transplantation, Laboratory for Inborn Errors of Immunity, KU Leuven, Leuven, Belgium; 11 Dept. of Pediatrics, Pediatric Immunodeficiencies Division, University Hospitals Leuven, Leuven, Belgium; 12 Institute of Immunity and Transplantation, Division of Infection and Immunity, University College London, London, UK; 13 Dept. of Immunology, Royal Free London NHS Foundation Trust, London, UK; 14 Division of Hematology/Oncology, Boston Children’s Hospital, Harvard Medical School, Boston, MA, USA; 15 Dept. of Pediatric Oncology, Dana-Farber Cancer Institute, Harvard Medical School, Boston, MA, USA; 16 Broad Institute of Massachusetts Institute of Technology and Harvard, Cambridge, MA, USA; 17 Allergy Centre, Union Hospital, Hong Kong, China; 18 Centre for Molecular Medicine and Therapeutics, BC Children’s Hospital Research Institute, Vancouver, Canada; 19 Dept. of Medical Genetics, University of British Columbia, Vancouver, Canada; 20 Dept. of Medical Genetics, The University of British Columbia, Vancouver, Canada; 21 Genome Science and Technology Program, Faculty of Science, The University of British Columbia, Vancouver, Canada; 22 Dept. of Pathology, Queen Mary Hospital, Hong Kong, China; 23 Dept. of Pathology, Emory University, Atlanta, GA, USA; 24 Unit of Immunology and Vaccinology, Division of General Pediatrics, Dept. of Woman, Child, and Adolescent Medicine, Geneva University Hospitals and Faculty of Medicine, University of Geneva, Geneva, Switzerland; 25 Genome Science and Technology Graduate Program, University of British Columbia, Vancouver, Canada; 26 Dept. of Paediatrics, University of Toronto, Toronto, Canada; 27 Molecular Immunology and Inflammation Branch, National Institute of Arthritis, Musculoskeletal and Skin Diseases, NIH, Bethesda, MD, USA; 28 Dept. of Pediatrics, Division of Allergy and Immunology, Children’s Hospital of Philadelphia, Philadelphia, PA, USA; 29 Pediatrics, Division of Human Genetics, Children’s Hospital of Philadelphia, Philadelphia, PA, USA; 30 Pediatrics, Division of Pediatric Dermatology, Perelman School of Medicine, University of Pennsylvania, Philadelphia, PA, USA; 31 NIAID Collaborative Bioinformatics Resource, NIAID, NIH, Bethesda, MD, USA; 32 Immunology Service, Clinical Center, NIH, Bethesda, MD, USA; 33 Dept. of Pediatrics, University of California San Diego, La Jolla, CA, USA; 34 Dept. of Medicine, Divison of Dermatology, The University of Hong Kong, Hong Kong, China; 35 Dept. of Paediatrics and Adolescent Medicine, Queen Mary Hospital, Hong Kong, China; 36 Dept. of Human Genetics, Radboud University Medical Center, Nijmegen, Netherlands; 37 Virtus Medical, Hong Kong, China; 38 Pediatric Center, Vinmec Times City International General Hospital, Hanoi, Vietnam; 39 Pediatrics, Pediatric Gastroenterology and Hepatology Research Center, Pediatrics Centre of Excellence, Children’s Medical Center, University of Medical Sciences, Tehran, Iran; 40 Pediatrics, Allergy and Clinical Immunology, Lorestan University of Medical Sciences, Khoramabad, Iran; 41 Dept. of Medical Biotechnology, School of Medicine, Zanjan University of Medical Sciences, Zanjan, Iran; 42 School of Mathematics, Institute for Research in Fundamental Sciences, Tehran, Iran; 43 Dept. of Medical Genetics, School of Medicine, Shahid Beheshti University of Medical Sciences, Tehran, Iran; 44 Microbiology and Immunology, Laboratory of Clinical Bacteriology and Mycology, KU Leuven, Leuven, Belgium; 45 DPUO, Research Unit of Infectivology and Pediatrics Drugs Development, Bambino Gesù Children Hospital IRCCS, Rome, Italy; 46 Laboratory of Medical Genetics, Translational Cytogenomics Research Unit, Bambino Gesù Children Hospital IRCCS, Rome, Italy; 47 Allergy Unit, Area of Translational Research in Pediatric Specialities, Bambino Gesù Children’s Hospital, IRCCS, Rome, Italy; 48 Dept. of Microbiology, Immunology and Transplantation, Allergy and Clinical Immunology Research Group, KU Leuven, Leuven, Belgium; 49 Dept. of Pediatrics, Pediatric Allergy Division, University Hospitals Leuven, Leuven, Belgium; 50 Dept. of Pediatrics, Pediatric Pulmonology Division, AZ Sint-Jan Brugge, Brugge, Belgium; 51 Dept. of Pediatrics, Pediatric Pulmonology Division, University Hospitals Leuven, Leuven, Belgium; 52 Dept. of Pediatrics, Pediatric Gastroenterology Division, AZ Sint-Jan Brugge, Brugge, Belgium; 53 Genetics Program, North York General Hospital, Toronto, Canada; 54 Depts. of Pediatrics and Clinical Genetics, Amsterdam University Medical Centers, Amsterdam, Netherlands; 55 Howard Hughes Medical Institute, The Rockefeller University, New York, NY, USA; 56 Department of Pediatrics, Necker Hospital for Sick Children, AP-HP, France; 57 Department of Pediatrics, Children’s Medical Center, Tehran University of Medical Sciences, Tehran, Iran; 58 Dept. of Immunology, Allergy and Rheumatology, Division of Primary Immunodeficiency, Vietnam National Children’s Hospital, Hanoi, Vietnam; 59 Faculty of Health Sciences, McMaster University, Hamilton, Canada; 60 St. Giles Laboratory of Human Genetics of Infectious Diseases, Rockefeller Branch, The Rockefeller University, New York, NY, USA

## Abstract

STAT6 (signal transducer and activator of transcription 6) is a transcription factor that plays a central role in the pathophysiology of allergic inflammation. We have identified 16 patients from 10 families spanning three continents with a profound phenotype of early-life onset allergic immune dysregulation, widespread treatment-resistant atopic dermatitis, hypereosinophilia with esosinophilic gastrointestinal disease, asthma, elevated serum IgE, IgE-mediated food allergies, and anaphylaxis. The cases were either sporadic (seven kindreds) or followed an autosomal dominant inheritance pattern (three kindreds). All patients carried monoallelic rare variants in *STAT6* and functional studies established their gain-of-function (GOF) phenotype with sustained STAT6 phosphorylation, increased STAT6 target gene expression, and T_H_2 skewing. Precision treatment with the anti–IL-4Rα antibody, dupilumab, was highly effective improving both clinical manifestations and immunological biomarkers. This study identifies heterozygous GOF variants in *STAT6* as a novel autosomal dominant allergic disorder. We anticipate that our discovery of multiple kindreds with germline *STAT6* GOF variants will facilitate the recognition of more affected individuals and the full definition of this new primary atopic disorder.

## Teaser

Sharma et al. define a new primary atopic disorder caused by heterozygous gain-of-function variants in STAT6. This results in severe, early-onset allergies, and is seen in 16 patients from 10 families. Anti–IL-4Rα antibody and JAK inhibitor treatment were highly effective.

## Introduction

Asthma and related atopic diseases, including atopic dermatitis, food allergy, allergic rhinitis, and eosinophilic gastrointestinal diseases, are estimated to affect ∼20% of the global population imposing immense health and economic burdens (Dierick et al., 2020). Identifying human single-gene defects that lead to severe allergic disease—so-called primary atopic disorders (PADs)—is a powerful strategy to define the cellular and molecular mechanisms that drive human allergic inflammation (Lyons and Milner, 2018; Vaseghi-Shanjani et al., 2021). Identifying new PADs accelerates the diagnosis and treatment of affected individuals and can uncover new molecular targets for preventing and treating common allergic disease.

Currently there are only a few known inborn errors of immunity (IEIs) underlying severe allergic disease (Milner, 2020). Indeed, most cases are of unknown etiology, particularly those that are isolated or sporadic. In this study, we describe a novel human PAD caused by germline heterozygous gain-of-function (GOF) variants in the gene *STAT6* found in 16 individuals from 10 unrelated families spanning three continents. Signal transducer and activator of transcription 6 (STAT6) is the main transcription factor that mediates the biological effects of IL-4, a key cytokine necessary for type 2 differentiation of T cells, B cell survival, proliferation, and class switching to IgE (Goenka and Kaplan, 2011; Takeda et al., 1996; Villarino et al., 2020; Villarino et al., 2017), as well as that of IL-13, a cytokine linked to anaphylaxis (Gowthaman et al., 2019). Affected individuals experienced early-onset severe, sometimes fatal, multisystem allergic disease which was refractory to conventional treatments. Notably, precision therapeutics aimed at targeting exaggerated STAT6 signaling were beneficial in those who received them.

## Results

### Identification of 16 patients from 10 families with severe early-onset allergic disease heterozygous for rare damaging *STAT6* variants

**Figure 1.**
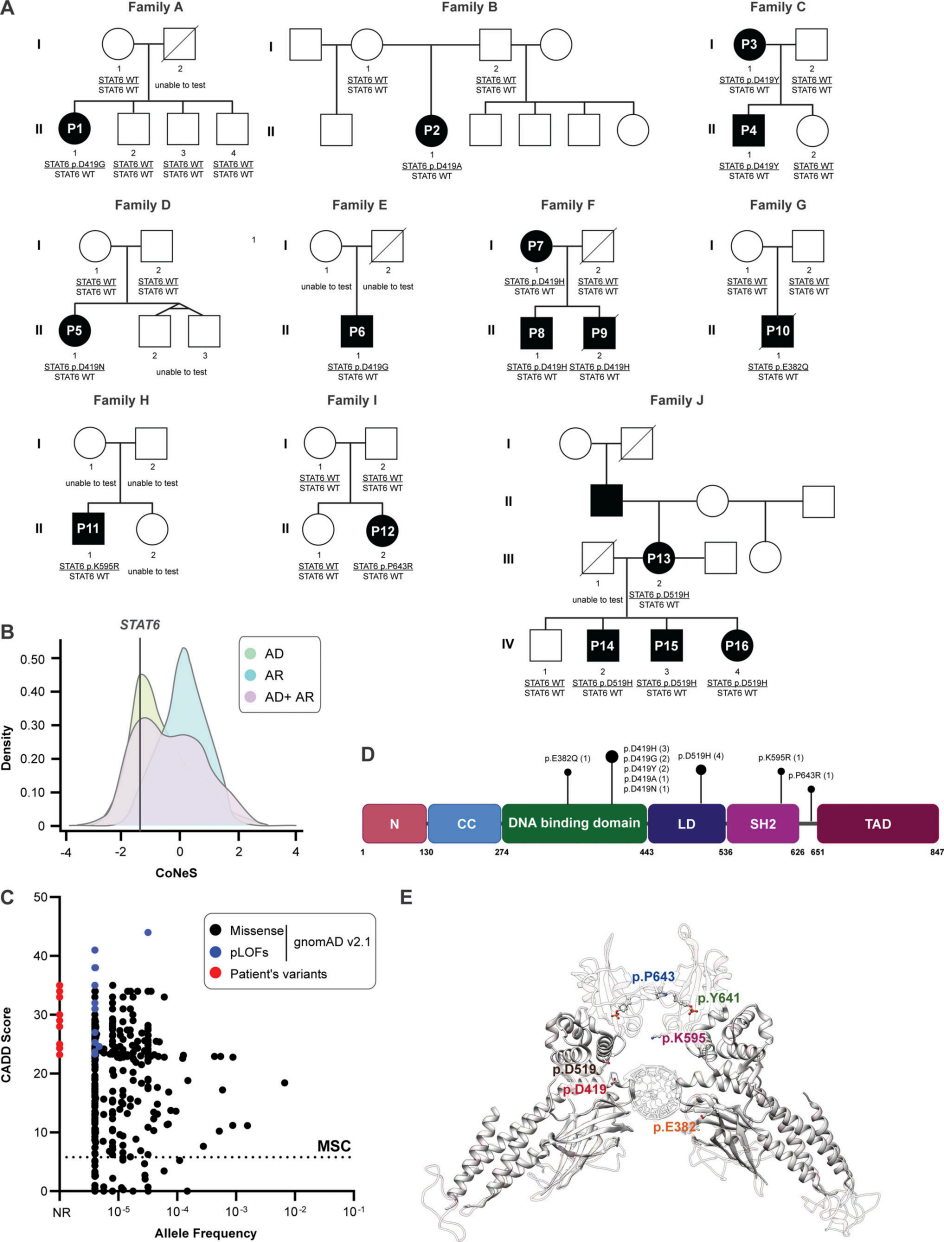
**16 patients with severe allergic disease and *STAT6* variants in different protein domains. (A)** Family pedigree of the 16 patients from 10 different families. Filled symbols = affected individual; unfilled symbols = unaffected individual. **(B)** Consensus negative selection (CoNeS) score for *STAT6* in relation to the score for known IEI genes reported with inheritance pattern of either AD, AR, or both (AD + AR). **(C)** Frequency and CADD score for missense (black) and predicted LOF (pLOF, blue) *STAT6* variants reported in a public database and STAT6 variants reported in our patient cohort (red). The dotted line corresponds to the mutation significance cutoff (MSC). **(D)** Schematic illustrating the protein domains of STAT6. Amino acid location of the variants shown are highlighted, with the length of the bar corresponding to the number of patients reported with variants at that site. **(E)** Structural model of the DNA-STAT6 homodimer complex showing location of the different STAT6 variants in relation to the DNA-binding interface.

**Figure S1.**
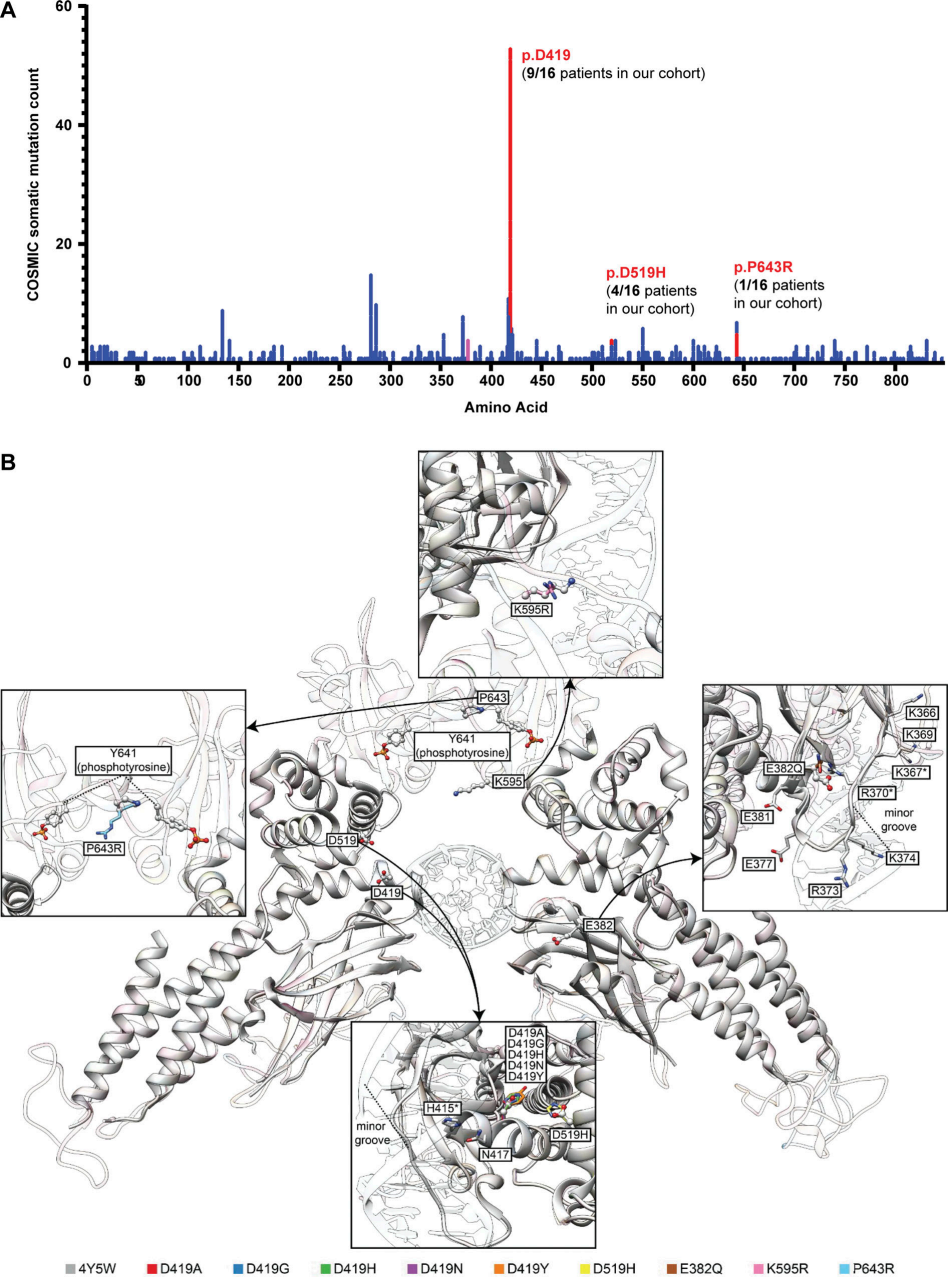
**Pathogenic *STAT6* germline variants lie in different protein domains and are frequently identified as somatic variants. (A)** Somatic mutation counts for different amino acid changed as reported by COSMIC for STAT6. Red highlighted changes are those germline variants also identified in our cohort that cause severe allergic disease. **(B)** Structural model of the DNA-STAT6 homodimer complex showing location of the different STAT6 variants in relation to the DNA-binding interface. Specifically, zoom-ins for variants at each location are shown in relation to previously described variants known to affect STAT6 function.

We studied 16 patients from 10 kindreds with severe early-onset allergic disease spanning three continents. Patients were identified by their expert clinicians as candidates for genetic assessment based on their extreme phenotype and, in some cases, their family history (see clinical narratives in Data S1; Tables S4 and S5; and Fig. 1 A). The patients were from diverse ethnicities, specifically European (Kindred D, F, and J), Middle Eastern (Kindred A and C), Hispanic (Kindred B), South Asian (Kindred H), East Asian (Kindred E), and Southeast Asian (Kindred Y). The cases were either sporadic (seven kindreds) or affected multiple individuals of either sex over different generations consistent with autosomal dominant (AD) inheritance (Kindreds C, F, and J). All patients carried monoallelic rare variants in *STAT6* (NM_001178079.2). The consensus negative selection score of STAT6 reveals a negative selection score that overlaps with known AD IEIs (Rapaport et al., 2021), consistent with the AD inheritance pattern observed in Kindreds C, F, and J (Fig. 1 B). In addition, and also consistent with an AD disorder, by sequencing both healthy parents (when available) we established that the STAT6 mutation was de novo in Patient 2 (P2), P5, P10, and P12 (from Kindreds B, D, G, and I, respectively; Fig. 1 A). The disease was fully penetrant in the families studied, as all *STAT6* variant carriers were affected. None of the variants have previously been reported in population databases (Fig. 1 C; i.e., gnomAD). All the variants were private to the studied kindreds, except the p.D419G variant which was common in Kindreds A and E. Pathogenicity prediction models identify all of these variants to be pathogenic, evidenced by high pathogenic CADD (Rentzsch et al., 2021), SIFT (Sim et al., 2012), and Polyphen-2 (Adzhubei et al., 2010) scores (Tables S4 and S5). Remarkably, nine patients from six kindreds carried a variant affecting amino acid D419. Importantly, variants leading to amino acid changes at p.D419, p.D519, and p.P643 can be found in the Catalogue of Somatic Mutations in Cancer (COSMIC) database as recurrent somatic variants in lymphoma with some experimental evidence for a GOF phenotype for variants at p.D419 (Yildiz et al., 2015; Zamò et al., 2018; Fig. S1 A). The reported variants lie in different protein domains of STAT6, including the DNA-binding domain (p.E382 and p.D419), the linker domain (p.D519), and the SH2 domain (p.K595), while p.P643 lies in close proximity to the p.Y641 phosphorylation site (Fig. 1, D–E). Although the variants were located within different domains of the STAT6 protein, modeling of STAT6 interacting with DNA reveals that all the identified variants (with the exception of p.P643) lie near the protein–DNA interface and result in amino acids changes leading to increased electro-positivity at physiological pH (Fig. 1 E). Notably, E382 and D419 are located in regions responsible for protein–DNA recognition (Li et al., 2016). Changes in these variants decrease the electro-negativity of the protein near the DNA-binding interface and are predicted to enhance STAT6 binding to DNA (Fig. 1 E and Fig. S1 B). In aggregate, these data suggest that the STAT6 germline monoallelic variants identified in the patients underlie severe allergic disease by a GOF mechanism.

### Unifying clinical features of the 16 patients with severe allergic disease

**Figure 2.**
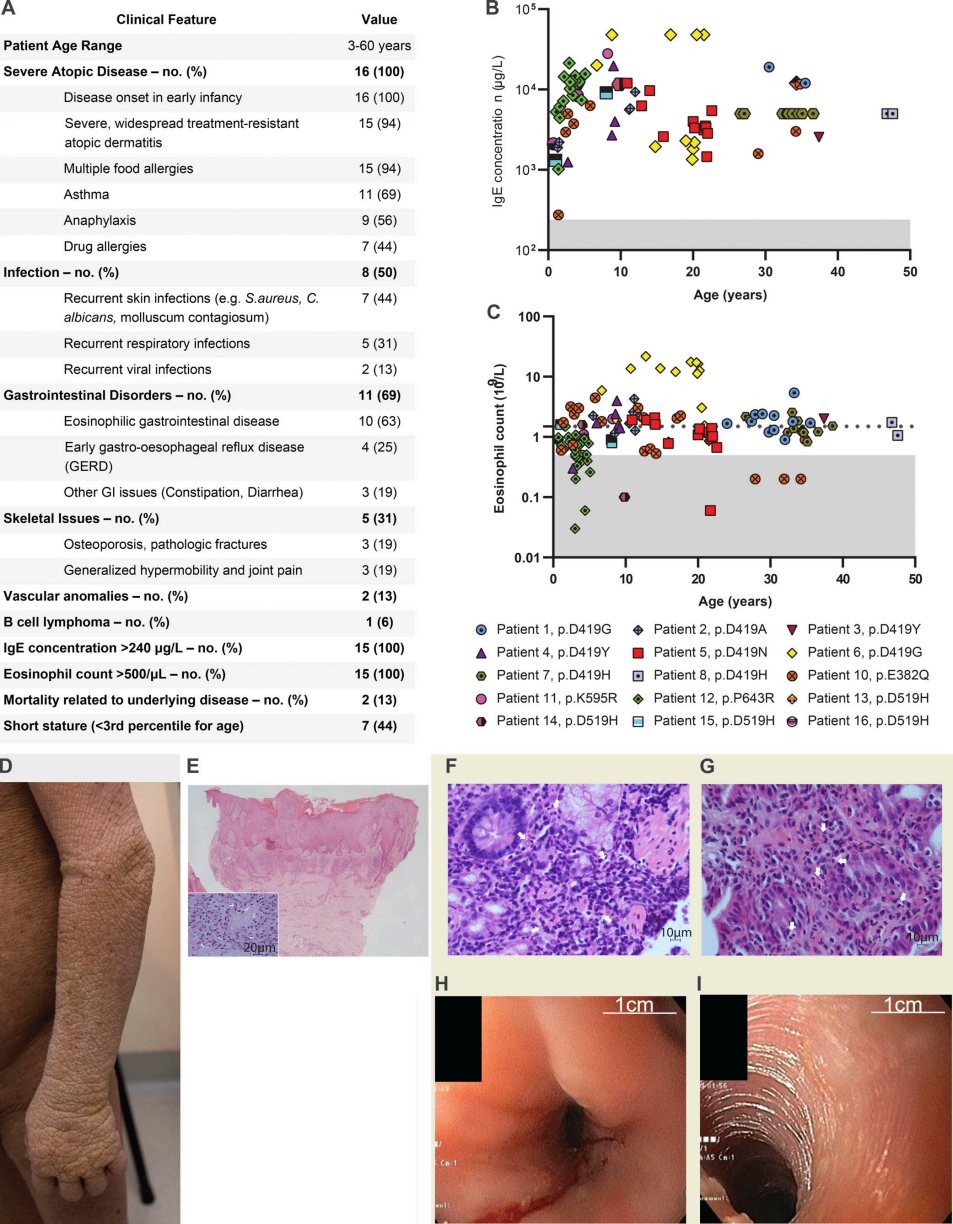
**Major clinical features of the 16 patients. (A)** Tabulation and comparison of the clinical phenotype for 16 patients. Please note blood eosinophil and IgE values were only available for 15 patients. **(B)** IgE concentration in whole blood for 15 out of the 16 patients. Shaded area represents IgE < 240 µg/liter, which is the typical upper limit of normal. **(C)** Eosinophil count in whole blood for 15 out of the 16 patients. Shaded area represents counts <0.5 × 10^9^/liter, which is the typical upper limit of normal. The horizontal broken line denotes an eosinophil count of 1.5 × 10^9^/liter, since hypereosinophilic syndrome is traditionally defined as peripheral blood eosinophilia >1.5 × 10^9^/liter persisting ≥6 mo. **(D)** Photograph of widespread and severe atopic disease. **(E)** Photomicrograph of the skin biopsy showing marked pseudoepitheliomatous hyperplasia with acanthosis, hyperkeratosis, and focal parakeratosis, suggestive of lichen simplex chronicus (H&E stain, original magnification 2×). Moderate chronic inflammation within the papillary dermis in which scattered eosinophils (white arrows) are conspicuous (inset, H&E stain; original magnification, 40×). **(F)** Photomicrograph of duodenal biopsy showing abundant eosinophils (white arrows) amongst lymphocytes (H&E stain; original magnification, 40×). **(G)** Photomicrograph of gastric antral biopsy showing abundant infiltrate of eosinophils (arrows) amongst lymphocytes and plasma cells (H&E stain; original magnification, 40×). **(H and I)** Endoscopic images showing (H) furrowing and (I) trachealization in the middle esophagus, suggestive of eosinophilic esophagitis.

**Figure S2.**
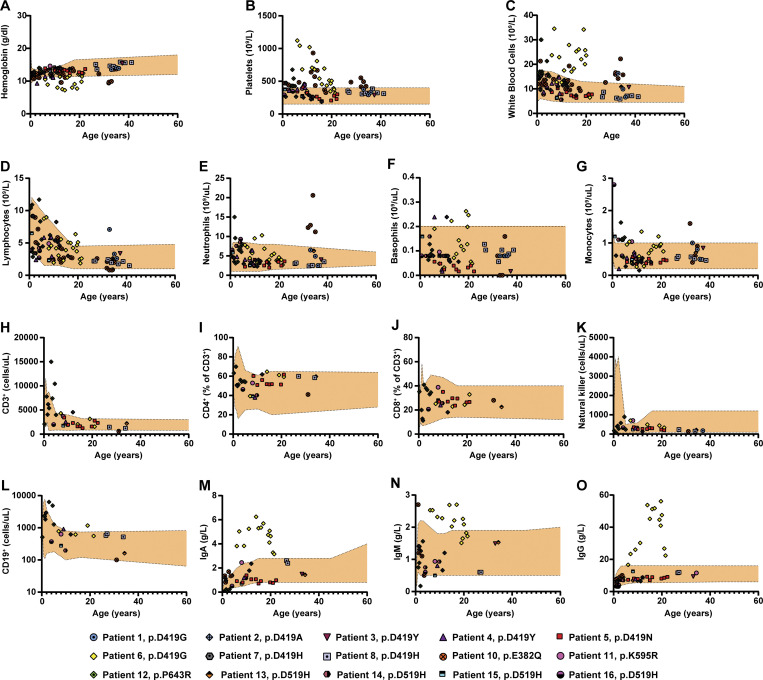
**Complete blood counts and immunological workup of patients with pathogenic STAT6 variants. (A–G)** Complete blood count for 15 out of the 16 patients and age-based references (orange-shaded area) for the following populations: (A) hemoglobin, (B) platelets, (C) white blood cells, (D) lymphocytes, (E) neutrophils, (F) basophils, and (G) monocytes. **(H–L)** Immunological workup for 15 out of the 16 patients showing age-based references (orange-shaded area) and populations quantification for: (H) CD3^+^ T cells, (I) CD4^+^ CD3^+^ T cells, (J) CD8^+^ CD3^+^ T cells, (K) NK cells, and (L) CD19^+^ B cells. **(M–O)** Immunoglobulin concentrations for 15 out of the 16 patients showing age-based references (orange-shaded area): (M) IgA, (N) IgM, and (O) IgA.

The patients in the cohort were aged from 3 to 60 yr. Full clinical narratives are provided in Data S1, and their clinical features are summarized in Fig. 2 A. All had severe allergic disease which began in their first year of life. Severe, treatment-resistant atopic dermatitis (15/16) and food allergies (15/16) were the most common clinical manifestations, followed by asthma (11/16) and eosinophilic gastrointestinal disease (10/16) and severe episodes of anaphylaxis (9/16). Clinical laboratory testing was notable for eosinophilia and markedly elevated serum IgE levels (Fig. 2, B and C). Other aspects of the clinical laboratory and immunological work up were largely unremarkable, although clinical hallmarks of chronic systemic inflammation were present in some patients (i.e., elevations in white blood cell counts, platelets, and serum immunoglobulin levels). T, B, and natural killer (NK) cell numbers were all typically in the normal range (Fig. S2). Clinical biopsies confirmed the presence of eosinophils in the skin and gastrointestinal tract (Fig. 2, E–G). Endoscopic visualization of the esophagus revealed trachealization and furrowing consistent with eosinophilic esophagitis (Bolton et al., 2018; Fig. 2, H and I).

In addition to atopic disease, half of the patients presented with recurrent skin, respiratory, and viral infections, although there were no deep-seated or fatal infections. Short stature (less than third percentile for age) was a common feature (7/16), and 5/16 had other skeletal issues such as pathologic fractures and generalized hypermobility. Finally, two of the patients died from their disease; one from anaphylaxis at the age of 20 yr and another from a cerebral aneurysm at age 35 yr. These clinical presentations highlight the severity of the multi-system disease found in this patient cohort.

### Functional analysis of the *STAT6* variants confirms their GOF activity

**Figure 3.**
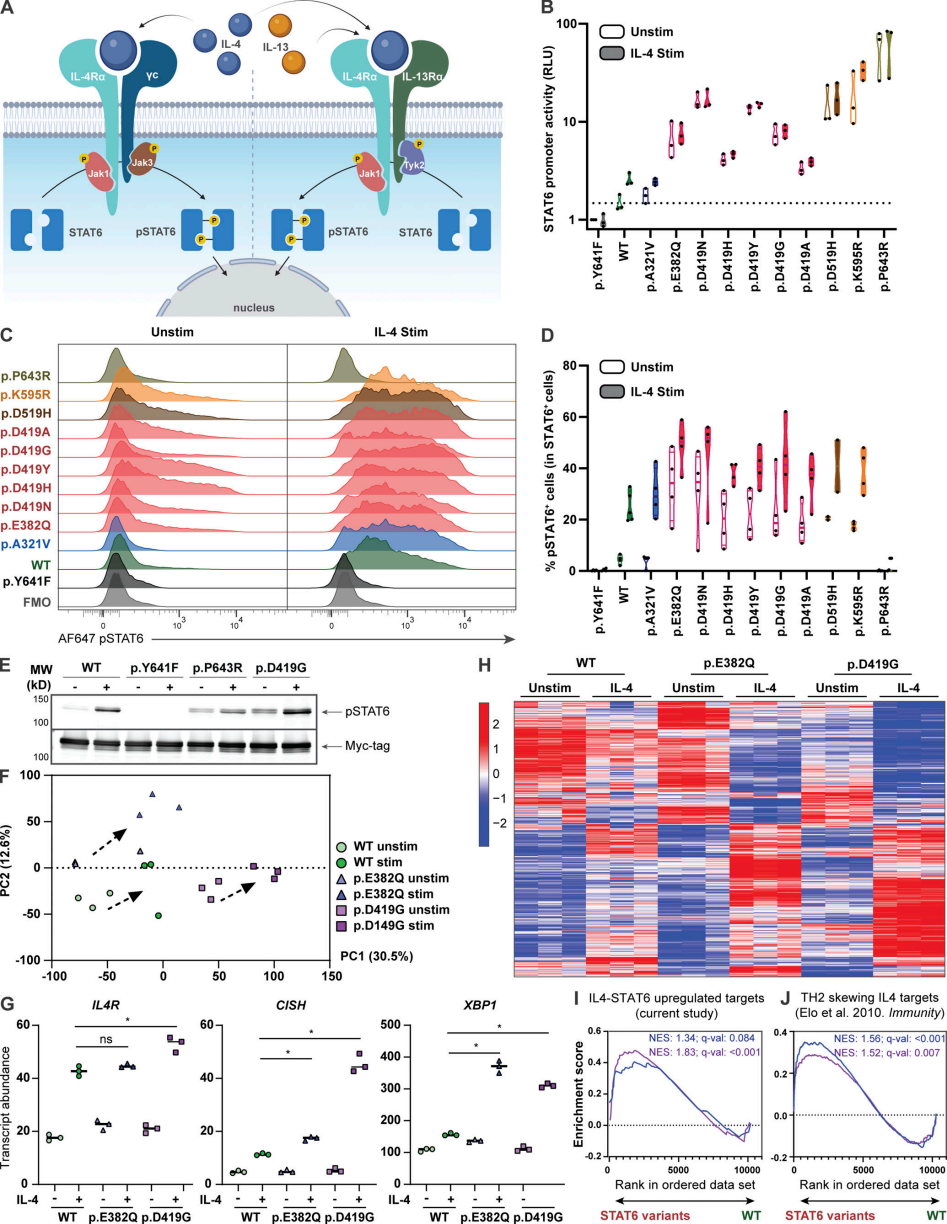
***STAT6* variants lead to increased STAT6 activity in HEK293 cells and Jurkat T cells. (A)** Schematic illustrating classical IL-4–mediated STAT6 activation, dimerization, and phosphorylation. **(B)** Luciferase assay of STAT6 activity on a plasmid containing a 4× STAT6 binding site (TTCCCAAGAA; the underlined bases represent two half-sites for STAT6-specific binding) for WT-, different *STAT6* variant–transfected HEK293 cells before and after stimulation with IL-4 (0.02 ng/ml for 4 h); *n* = 3. **(C)** Phospho-STAT6 (Y641) expression in WT- and *STAT6* variant–transfected HEK293 cells before and after treatment with IL-4 (10 ng/ml for 30 min). Gating strategy for pSTAT6^+^ cells can be found in Fig. S3 C. **(D)** Quantification of C; *n* = 4. **(E)** Immunoblot in HEK293 cells transfected with WT-, inactive- (p.Y641F), p.P643R-, and p.D419G-STAT6 variants for pSTAT6, and Myc-tag before and after treatment with IL-4 (10 ng/ml for 30 min); *n* = 3. Full-length immunoblot for this can be found in Fig. S3, D and E. **(F)** Principal component analysis (PCA) comparing unstimulated and stimulated (100 ng/ml IL-4 for 4 h) WT (green), p.E382Q (blue), and p.D419G (purple) *STAT6*-transduced Jurkat T cells. Individual symbols represent technical replicates of one transduced pool for each genotype. PC1 and PC2 contribution is shown in brackets. **(G)** Normalized counts comparing stimulated WT (green) vs. p.E382Q (blue) or p.D419G (purple), for *IL4R*, *CISH*, and *XBP1*. **(H)** Heatmap representation of normalized counts of a transcription set defined as IL-4 targets in transduced Jurkat T cells. **(I and J)** Asterisk indicates adjusted P value <0.05. GSEA plots for (I) curated STAT6 target genes comparing WT vs. either p.E382Q (blue) or p.D419G (purple) at baseline, or (J) IL-4-T_H_2 targets genes comparing WT vs. either p.E382Q (blue) or p.D419G (purple) after stimulation with IL-4. Normalized enrichment score and adjusted P value are shown. Source data are available for this figure: SourceData F3.

**Figure S3.**
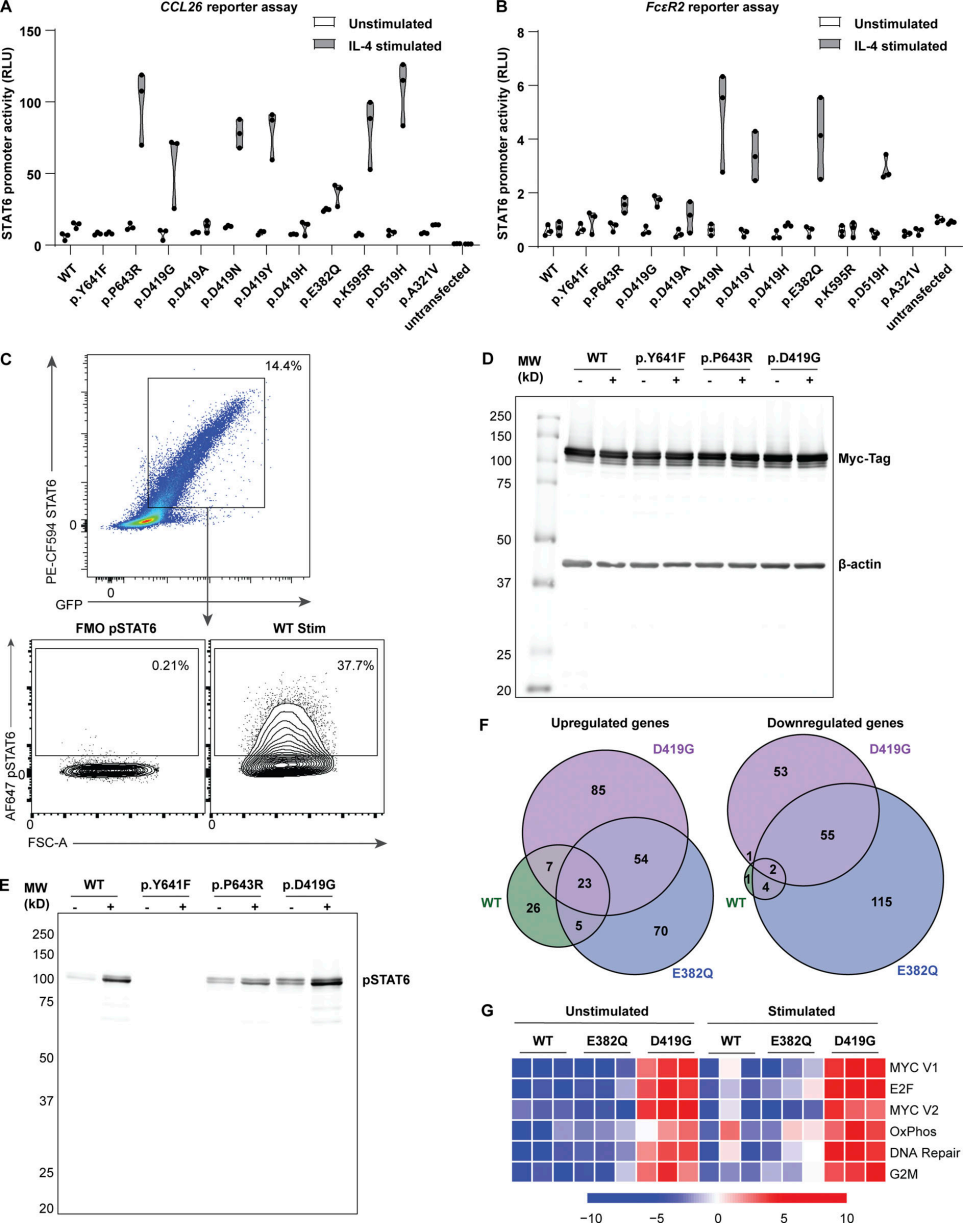
**In vitro assays demonstrate that STAT6 variants lead to increased STAT6 activity. (A and B)** Luciferase assay of STAT6 activity on a plasmid containing (A) *CCL26* promoter and (B) *FcεR2* promoter for WT-, different *STAT6*-variant transfected HEK293 cells before and after stimulation with IL-4 (100 ng/ml for 40 h), *n* = 3. **(C)** Gating strategy for determining % positive HEK293 pSTAT6 cells: dot plot for fluoresence minus one (FMO) is presented and was used for establishing pSTAT6^+^ cells. **(D and E)** Full-length immunoblots of the cropped immunoblots shown in Fig. 3 E, showing HEK293 cells transfected with WT-, inactive- (p.Y641F), p.P643R-, and p.D419G- STAT6 variants for (D) Myc-tag and β-actin, as well as (E) pSTAT6 before and after treatment with IL-4 (10 ng/ml for 30 min). **(F)** Significantly upregulated (i) and downregulated (ii) genes upon IL-4 treatment in WT (green), p.E382Q (blue), and p.D419G (purple) in Jurkat cells as shown through Venn diagram. **(G)** Sample level enrichment analyses of significantly enriched immune pathways from MSigDB Hallmark in unstimulated and IL-4–stimulated samples, comparing WT vs. either p.E382Q or p.D419G. Heatmap is normalized across the rows and shown as relative expression.

To assess the functional impact of the *STAT6* variants, we selected HEK293 cells as our model system, as these cells lack endogenous STAT6 but express other components of the IL-4R pathway (Fig. 3 A; Mikita et al., 1996). HEK293 cells were transfected with each of the 10 patient *STAT6* variants along with three different controls: WT *STAT6*, a predicted damaging *STAT6* population variant found in the gnomAD healthy population database (p.A321V), and a biochemically inactive *STAT6* variant (p.Y641F; Wick and Berton, 2000). To investigate STAT6 transcription factor activity, we conducted luciferase assays with three different reporter plasmids containing STAT6 binding sites (Li et al., 2016). While there were some difference related to the specific combinations of reporter plasmids and patient variants, there was evidence of GOF activity with all STAT6 patient variants resulting in higher promoter activity at baseline and after stimulation compared to the controls (Fig. 3 B and Fig. S3, A and B). The phosphorylation status of STAT6 variants was also quantified by flow cytometry and was confirmed to be higher at baseline and after stimulation compared to WT (Fig. 3, C and D; and Fig. S3 C). STAT6 phosphorylation was not detectable by flow cytometry for the p.P643R variant; however, increased baseline phosphorylation was confirmed by immunoblotting (Fig. 3 E and Fig. S3, D and E). This may point to a conformational change in tertiary structure of STAT6 near the phosphorylation site p.Y641 for this variant that rendered it inaccessible to the flow cytometry antibody.

We next evaluated if the increased promoter activity leads to global transcriptomic changes. As transcriptomic studies on HEK293 cells after IL-4 stimulation have been challenging to interpret (Yildiz et al., 2015), we stably expressed p.E382Q and p.D419G STAT6 by lentiviral transduction in Jurkat T cells, which express endogenous STAT6 and serve as a model of heterozygosity (Kim et al., 2012). Here, we observed that WT-, p.E382Q-, and p.D419G-transduced cells clustered separately from each other both at baseline and after stimulation with IL-4 (Fig. 3 F). Differential gene expression analysis revealed significantly increased transcript abundance of known STAT6 target genes, including *IL4R* (Goenka and Kaplan, 2011), *CISH* (Yildiz et al., 2015), and *XBP1* (Bettigole et al., 2015) in p.E382Q and p.D419G transduced cells when compared to WT transduced control (Fig. 3 G). Interestingly, p.E382Q and p.D419G had 67 and 80 uniquely increased hits, which did not overlap with WT nor with each other (Fig. 3 H, Fig. S3 F, and Table S6). This suggests that the altered activity of both p.E382Q and p.D419G is not restricted to enhanced activity of known STAT6 targets alone. Further investigation through gene set enrichment analyses (GSEA) showed increased enrichment in IL-4-STAT6 target genes at baseline (Fig. 3 I and Table S7), increased enrichment in T helper 2 (T_H_2) drivers after stimulation (Elo et al., 2010; Fig. 3 J and Table S7), and increased enrichment in proliferation pathways for p.D419G consistent with its known oncogenic activity (Ritz et al., 2009; Tate et al., 2019; Yildiz et al., 2015; Fig. S3 G).

### Patients with GOF *STAT6* variants have slower STAT6 dephosphorylation kinetics after IL-4 stimulation and an enhanced T_H_2 signature

**Figure S4.**
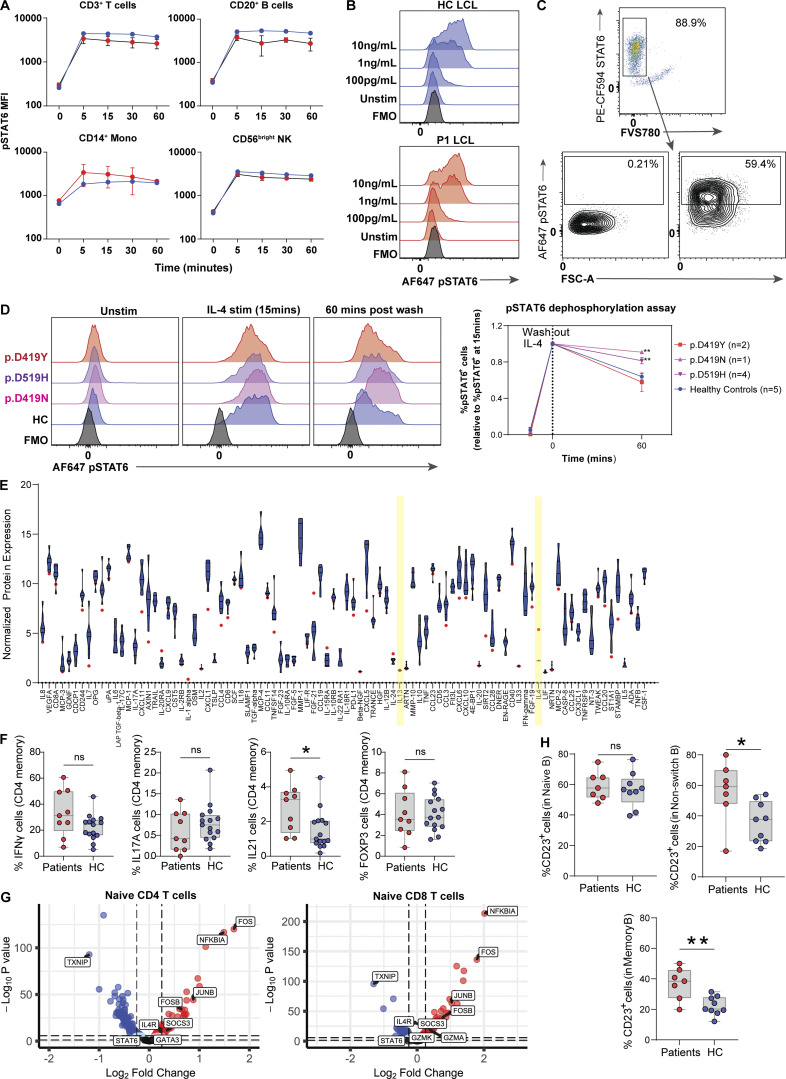
**Measure of STAT6 activity in patient primary lymphocytes. (A)** 1-h time course to measure phosphorylation of STAT6 in different populations of lymphocytes from five patients (red) and one healthy control (blue) after stimulation with IL-4 (10 ng/ml). **(B)** Dose response in LCLs of patient one (red) vs. one healthy control (blue) after stimulation of cells with various doses of IL-4 15 min. **(C)** Gating strategy to determine % pSTAT6 positive cells in LCLs: dot plot for FMO is presented and was used for establishing pSTAT6^+^ cells. **(D)** Histograms showing phosphorylation of STAT6 in healthy control (blue) and patients with genotype p.D419Y (red, *n* = 2), p.D519H (purple, *n* = 4), p.D419N (pink, *n* = 1), and healthy controls (blue, *n* = 5) in T cell blasts that were stimulated with IL-4 (10 ng/ml) for 15 min, washed with PBS, and subsequently incubated in IL-4–free media for 60 min. Quantification of pSTAT6^+^ cells is presented and normalized to max stimulation (noted at 15 min). Two-way ANOVA followed by Šídák’s multiple comparisons was conducted. **, P < 0.01. **(E)** Readout of 92 biomarkers for P5 using throughput Olink proteomics. Eight healthy control distribution are shown as a violin plot in blue. The patient is shown as a red circle. Key cytokines, IL-4 and IL-13, are highlighted in yellow. **(F)** T helper cell distribution for nine patients (red) and 15 age-matched healthy controls (blue) each. **(G)** Transcriptomic comparison of naive CD4^+^ and naive CD8^+^ T cells between P6 and one healthy control measured through scRNAseq. Red genes are enriched in patient; blue genes are enriched in healthy control. The two dotted lines are the P value and adjusted P value respectively. **(H)** Quantification of % CD23 positive cells in naive, non-class switched memory, and class-switched memory B cells between patients (red, *n* = 7) and healthy controls (blue, *n* = 9) after stimulation with IL-4 (10 ng/ml) for 20 h. Unpaired *t* test. *, P < 0.05; **, P < 0.01.

**Figure 4.**
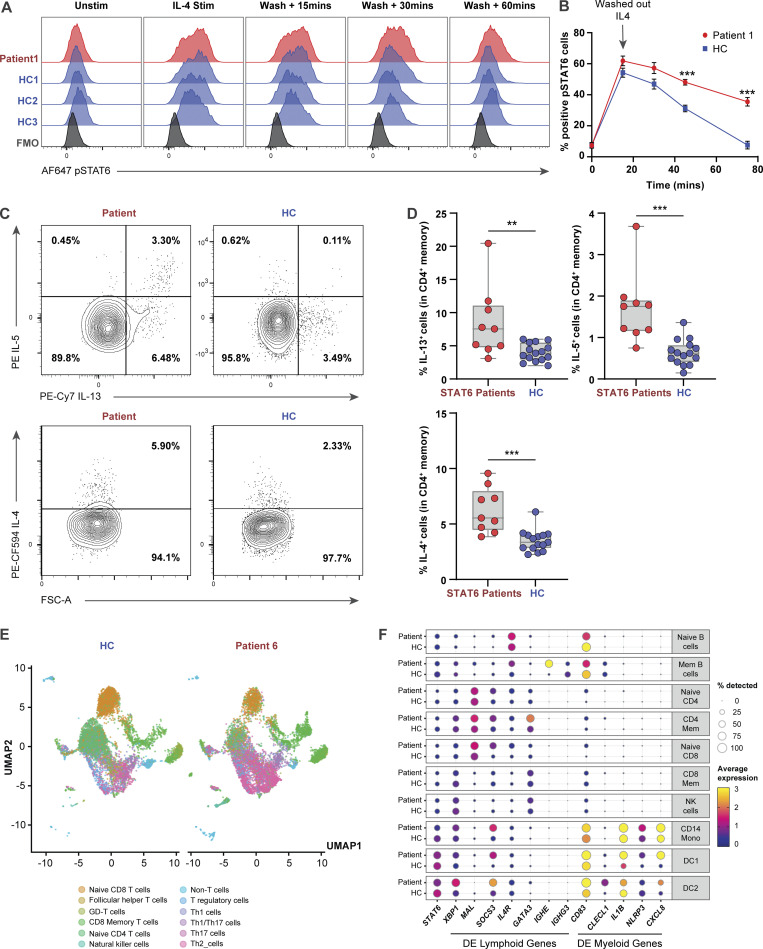
**Primary lymphocytes of STAT6 GOF patients display higher STAT6 activity and T**_**H**_**2 skewing. (A)** Histograms showing phosphorylation of STAT6 in healthy control (blue) and patient (red) LCLs that were stimulated with IL-4 (10 ng/ml) for 15 min, washed with PBS and subsequently incubated in IL-4–free media for 15, 30, and 60 min. Gating strategy for pSTAT6^+^ cells can be found in Fig. S4 C. **(B)** Quantification of pSTAT6^+^ cells from three separate experiments done in A, multiple unpaired *t* test corrected for multiple comparison using the Benjamini–Hochberg method. ***, P < 0.001. **(C)** Frequency of IL5^+^, IL13^+^, and IL4^+^ cells in memory CD4^+^ T cells of one representative patient, along with one representative healthy control. **(D)** Quantification of C showing IL5^+^, IL13^+^, and IL4^+^ cells in patients along with 15 age-matched healthy controls. **, P < 0.01; ***, P < 0.001. **(E)** Uniform manifold approximation and projection (UMAP) visualization of scRNAseq done on enriched T cells comparing one patient with one age-matched healthy control. **(F)** Dot plot showing expression of selected differentially expressed genes (adjusted P value < 0.05) observed in scRNAseq between patient and healthy control and associated with T cells, B cells, monocytes, or dendritic cells.

To further investigate the role of STAT6 GOF variants in primary cells, STAT6 phosphorylation was quantified in patient samples. Unexpectedly, we found no differences in the percentage of phospho-STAT6 positive cells between patients and healthy controls after IL-4 stimulation over a 60 min time course nor after stimulation with different doses of IL-4 (Fig. S4, A and B). However, differences emerged when we monitored the kinetics of STAT6 dephosphorylation after stimulation (Fig. 4, A and B; and Fig. S4 C). Specifically, washing out of IL-4 led to slower dephosphorylation kinetics of STAT6 in most patient cells compared to healthy controls (Fig. 4, A and B; and Fig. S4 D), supporting a GOF mechanism in patient lymphocytes. We did note that one of our kindreds did not display delayed dephosphorylation (Fig. S4 D), suggesting that this might not be the only GOF mechanism. Indeed, increased STAT6 activity without phosphorylation has previously been reported in follicular lymphoma research studying the p.D419 mutational hotspot (Yildiz et al., 2015).

Given that the STAT6 axis is critical for the differentiation of T_H_2 cells (Kaplan et al., 1996), a subset of CD4^+^ helper T cells that is a major contributor to the pathogenesis of allergic disease, we next investigated T_H_2 signatures in these patients. Patients showed skewing towards T_H_2 pathway activity compared to healthy controls based on higher levels of the T_H_2 cytokines IL-5, IL-13, and IL-4 as measured by flow cytometry (Fig. 4, C and D), or through transcriptomic signature by single-cell RNA sequencing (scRNAseq; Fig. 4 E). High throughput proteomics also validated the increased IL-4 expression, but not high IL-13 expression (Fig. S4 E). Differences in proportions of other subsets of helper T cells were restricted to higher IL-21^+^ cells in patient memory CD4^+^ T cells (Fig. S4 F). scRNAseq showed that patient B cells expressed high *IGHE* and low *IGHG3* (Fig. 4 F), reflecting patterns opposite of those seen in STAT6 knockout mice (Shimoda et al., 1996; Sulczewski et al., 2021), and CD4^+^ T cells express high GATA3. scRNAseq further demonstrated that *IL4R*, a gene encoding a key receptor that mediates STAT6 activation, was upregulated in all B and T cell subsets (Fig. 4 F and Fig. S4 G).

**Figure 5.**
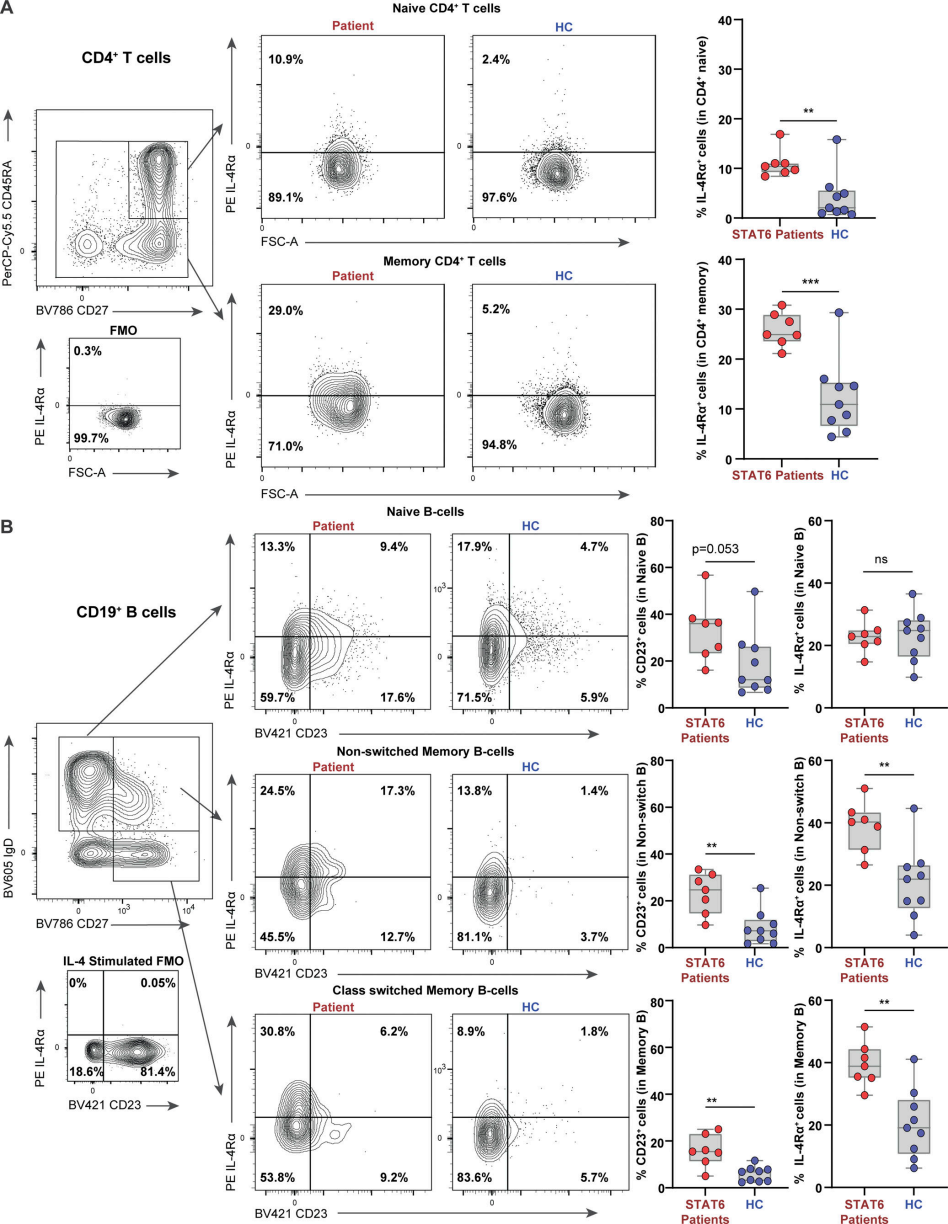
**Primary lymphocytes of STAT6 GOF patients display high expression of STAT6 target genes. (A)** Expression of IL4Rα in naive and memory CD4^+^ cells is quantified as % positive cells in primary patient cells (*n* = 7, red) and healthy controls (*n* = 9, blue). Gating strategy for naive and memory and CD4^+^ is presented along with a dot plot for a fluoresence minus one (FMO) IL-4Rα sample to display IL-4Rα^+^ gating, as well as a representative dot plot for a patient and healthy control. **(B)** Expression of CD23 and IL4Ra in naive, non-class switched memory and memory B cells is quantified as % positive cells in primary patient cells (*n* = 7, red) and healthy controls (*n* = 9, blue). Gating strategy for B cell subsets is presented along with a dot plot for an FMO IL-4Rα sample to display IL-4Rα^+^ gating, as well as a representative dot plot for a patient and healthy control. Unpaired *t* test. **, P < 0.01; ***, P < 0.001.

Using flow cytometry, we confirmed that IL-4Rα expression was significantly higher on both naive and memory CD4^+^ T cells of seven patients from three different kindreds compared to nine healthy controls (Fig. 5 A). IL-4Rα expression was also found to be higher in non-class switched memory and class switched memory B cells of unstimulated patient primary cells (Fig. 5 B). These findings suggest higher baseline activity of STAT6 in patient cells driving IL-4Rα expression similar to that seen in our Jurkat model (Fig. 3, G–J; and Tables S6 and S7). Finally, we measured the expression of CD23 (the low-affinity IgE receptor, FcεRII) which is known to be upregulated by STAT6 (Fig. S3 B; Kneitz et al., 2000) and we found higher expression of CD23 on all subsets of patient B cells at baseline (Fig. 5 B) and following stimulation with IL-4 (Fig. S4 H). Taken together, these experiments conducted using primary patient cells further confirm the STAT6 GOF phenotype.

### JAK inhibitors and IL-4Rα monoclonal antibody can be used as potential therapeutics for patients with *STAT6* GOF variants

Due to the severity of the multi-system allergic disease experienced by the patients in our cohort, various treatment approaches were used over the years. Corticosteroids, administered topically and systemically, were the mainstay of treatment for most patients. Unfortunately, even high doses of corticosteroids were unable to control the allergic inflammation and they were responsible for many side-effects including cataracts and osteoporosis. Other treatments used in this cohort that proved ineffective included topical tacrolimus, oral methotrexate, and mepolizumab (an anti–IL-5 monoclonal antibody).

**Figure 6.**
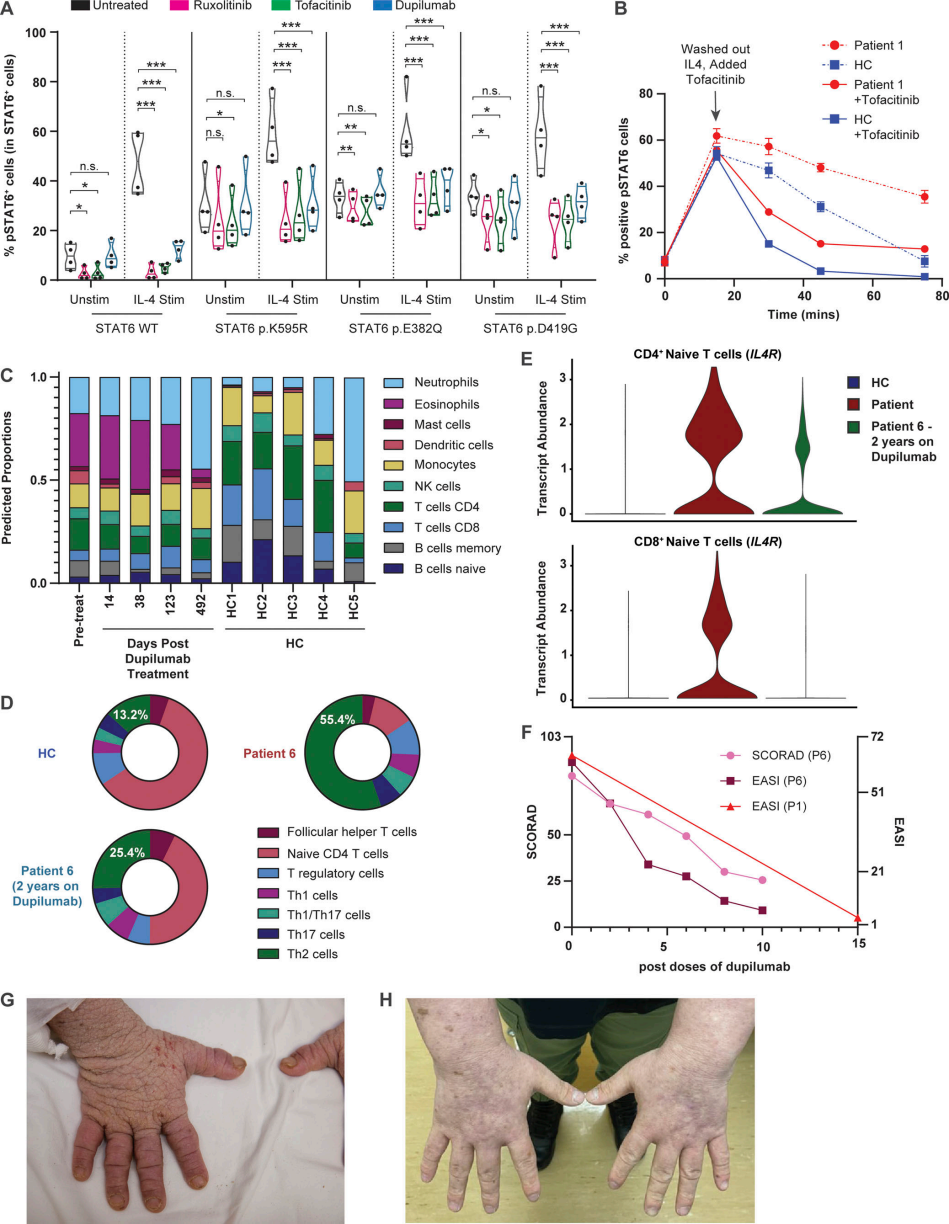
**JAK inhibitors and IL-4Rα antibody can be used as potential therapeutics for patients with GOF STAT6 variants. (A)** Quantification of phospho-STAT6 expression in transfected HEK293 cells left untreated (black) pre-treated with ruxolitinib (10 μM, 1 h; pink), tofacitinib (10 μM, 1 h; green), or dupilumab (10 nM, 1 h; blue), before and after stimulation with IL-4 (10 ng/ml, 30 min). Individual points represent separate transfections for each genotype (*n* = 4). Gating strategy for pSTAT6^+^ cells can be found in Fig. S3 C. One-way ANOVA and Tukey’s post-hoc test. *, P < 0.05; **, P < 0.01; ***, P < 0.001. **(B)** Quantification of pSTAT6^+^ cells in patient (red) and healthy control (blue) LCLs stimulated with IL-4 (10 ng/ml for 15 min), washed and incubated in tofacitinib (10 μM) for 15, 30, and 60 min. Dotted translucent lines are indicative of no tofacitinib treatment (Fig. 4 B); *n* = 1. Gating strategy for pSTAT6^+^ cells can be found in Fig. S4 C. **(C)** Cell type proportion gene signature as determined by the software Cibersort, in a patient undergoing dupilumab treatment for 2 yr and five healthy controls. Cell labels are listed on the right. **(D)** Donut plot showing frequencies of CD4^+^ T helper subsets in one patient, an age-matched healthy control (Fig. 4 E), and a 2-yr post-dupilumab treatment patient sample as measured by scRNAseq on enriched T cells. Frequency of T_H_2 cells is quantified in the donut plots of the different samples. **(E)** Violin plots showing expression of *IL4R* in the patient (red), healthy control (blue), and a 2-yr post-dupilumab sample (green). **(F)** Eczema scoring, EASI and SCORAD, for two patients after treatment with multiple doses of dupilumab. **(G and H)** Photographs of hands showing (G) the severity of atopic dermatitis before and (H) the improvement after dupilumab treatment.

**Figure S5.**
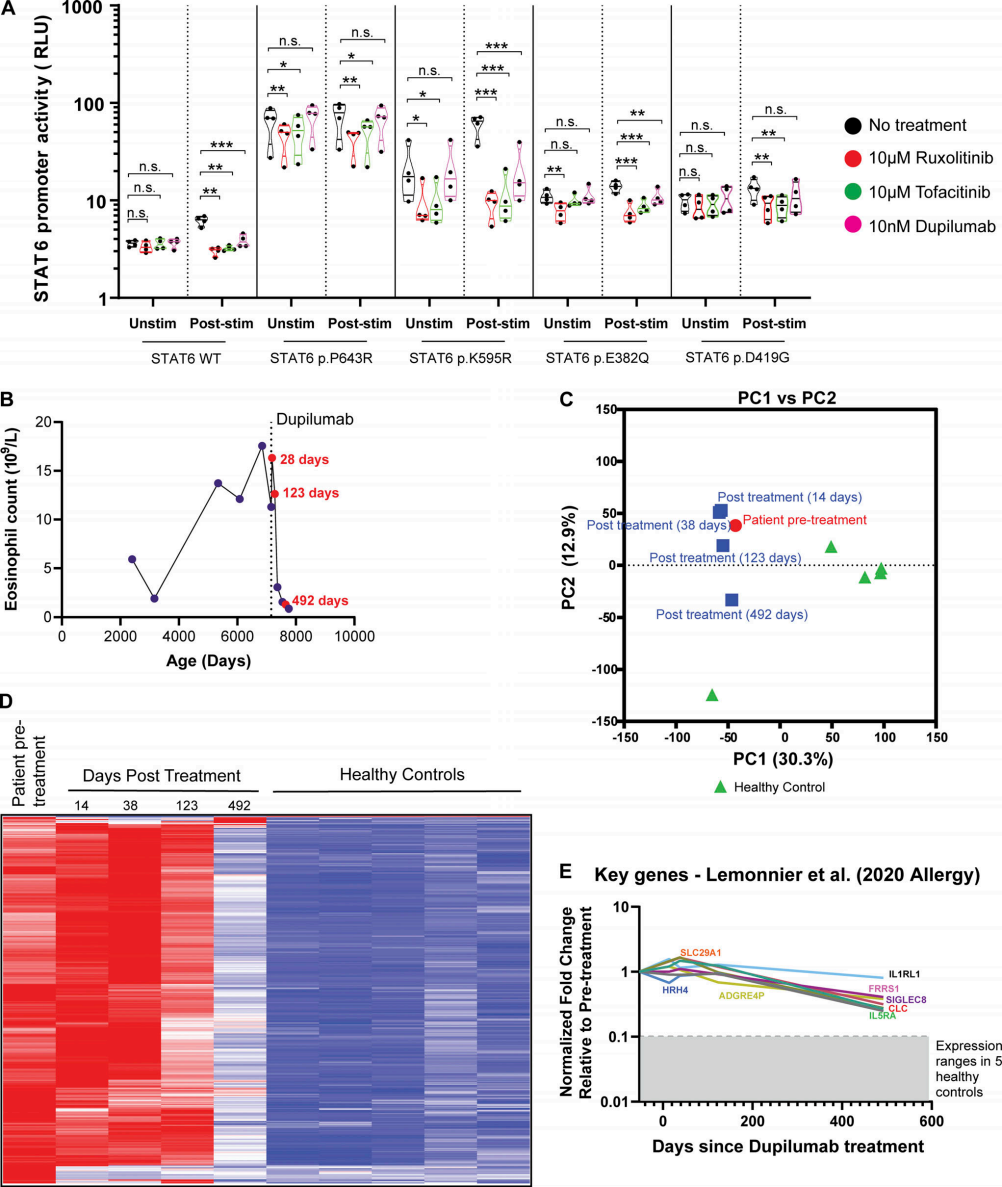
**STAT6 activity can be therapeutically targeted and can resolve clinical disease severity. (A)** Quantification of luciferase assay in HEK293 transfected cells pre-treated with ruxolitinib (10 μM, 1 h), tofacitinib (10 μM, 1 h), or dupilumab (10 nM, 1 h), before and after stimulation with IL-4 (0.02 ng/ml, 4 h). *n* = 4. One-way ANOVA and Tukey’s post-hoc test. *, P < 0.05; **, P < 0.01; ***, P < 0.001. **(B)** Eosinophil counts before and following initiation of treatment with dupilumab are presented. Dots in red corresponds to transcriptomic data from this patient presented in Fig. 6 C. **(C)** PCA comparing whole blood bulk RNAseq of P6 before treatment with dupilumab and four time points after treatment, alongside five healthy controls. **(D)** Heatmap signatures of differentially expressed genes comparing pre-treatment patient samples against five healthy controls. Genes are row normalized. **(E)** Key genes, previously described to be biomarkers for allergic disease (Lemonnier et al., 2020) in whole blood RNA are presented for the patient samples. Gray shaded area is the range for the expression of these genes in five healthy controls.

Having demonstrated that heterozygous *STAT6* variants lead to higher STAT6 activity and T_H_2 immunological skewing, we tested in vitro whether targeting the STAT6 pathway could be clinically beneficial. Based on our findings of higher phosphorylation of STAT6, and higher expression of IL-4Rα, we selected the JAK inhibitors, ruxolitinib and tofacitinib, and the anti–IL-4Rα antibody, dupilumab, as drugs to test in vitro as they are all used clinically for treatment of allergic disease (Bacharier et al., 2021; Beck et al., 2014; Bissonnette et al., 2016; Kim et al., 2020). We demonstrated that both ruxolitinib and tofacitinib effectively decreased the patient-specific enhanced STAT6 phosphorylation/activation in HEK293 cells at baseline and after IL-4 stimulation, whereas dupilumab inhibited IL-4 mediated increase in STAT6 activity (Fig. 6 A and Fig. S5 A). We further confirmed in patient primary cells that tofacitinib accelerated STAT6 dephosphorylation following IL-4 stimulation (Fig. 6 B). These data suggest the potential clinical benefit of directly targeting the IL-4/STAT6 pathway in patients with *STAT6* GOF variants.

Once their STAT6 GOF variant was identified, three of the patients were started on dupilumab and all showed significant clinical improvement. P6, who has been on treatment with dupilumab for over 2 yr, serves as an illustrative example. Mirroring peripheral blood eosinophil counts (Fig. S5 B), repeated whole blood RNAseq showed global transcriptomic changes that were suggestive of mildly increased eosinophilic and allergic gene signatures after 38 d, followed by a shift of the transcriptome towards healthy controls after 123 and 492 d, respectively (Fig. 6 C and Fig. S5, C–E). scRNAseq confirmed a decrease in T_H_2 gene signatures 2 yr following initiation of dupilumab, accompanied by a decrease in the expression of IL-4R on both naive CD4^+^ and CD8^+^ T cells (Fig. 6, D–E). Clinically, these changes were accompanied by dramatic increase in growth velocity, improved skin condition as quantified by SCORAD (SCORing atopic dermatitis) and EASI (eczema area and severity index) scores, and the ability to wean from oral corticosteroids (Fig. 6, F–H). Similarly, P1 experienced remarkable clinical benefits with dupilumab with improved skin inflammation (Fig. 6 D), resolution of pruritus, and the ability to discontinue oral daily high dose corticosteroids. In addition to resolution of skin inflammation with dupilumab, P2 was able to discontinue swallowed budesonide without a flare in the severity of her eosinophilic esophagitis. Our preclinical data also suggested that JAK inhibitors may be beneficial (Fig. 6, A and B), and P4 had received tofacitinib (5 mg/d) for 2 mo at the time this manuscript was finalized. His initial response to tofacitinib was encouraging with less dysphagia, less esophageal food impaction, improved endoscopic appearance of the esophagus, and a marked reduction in the number of intraepithelial eosinophils.

## Discussion

We present a combination of clinical, genetic, molecular, and transcriptional evidence of a new human disorder caused by germline AD GOF *STAT6* variants in 16 patients with life-long severe allergic disease. These variants led to sustained STAT6 phosphorylation, increased STAT6 target gene expression, and T_H_2 skewed transcriptional profile. Importantly, we demonstrate in three patients that dupilumab treatment is a highly effective targeted therapeutic option, improving both clinical manifestations of disease and immunological biomarkers.

Although the full phenotype(s) of individuals with GOF *STAT6* variants will only be uncovered through the identification of additional affected individuals, we propose to classify human germline AD GOF STAT6 syndrome as a PAD (Lyons and Milner, 2018; Milner, 2020; Vaseghi-Shanjani et al., 2021). Based on our study, possible clinical “red flags” for this new disorder include: (i) early life onset; (ii) peripheral blood eosinophilia; (iii) elevated serum IgE; (iii) widespread, treatment-resistant atopic dermatitis; (iv) multiple food and drug allergies; (v) severe (and even fatal) anaphylaxis; (vi) recurrent skin and respiratory infections; (vii) eosinophilic gastrointestinal disorder, including eosinophilic esophagitis; (viii) asthma; (ix) allergic rhinoconjunctivitis; (x) short stature; and possibly (xi) vascular malformations of the brain.

STAT6 is intimately linked to the biology of allergic inflammation. The central and most studied role of STAT6 is in mediating the biological effects of IL-4, a cytokine necessary for T_H_2 differentiation, B cell survival, proliferation, and class switching to IgE (Elo et al., 2010; Takeda et al., 1996), as well as in driving M2 macrophage polarization (Ginhoux et al., 2016). In T cells, STAT6 activation induces the expression of GATA3, the master regulator of T_H_2 differentiation, which in turn enhances expression of IL-4, IL-5, and IL-13, cytokines necessary for promoting allergic responses by activating mast cells and eosinophils (Sloka et al., 2011). The presence of greater T_H_2 cell populations, or T_H_2 cells producing copious amounts of IL-4/IL-5/IL-13, could be a driver of the observed allergic phenotype presented in our patients. Elevated IgE in partnership with mast cells is important for both acute and chronic manifestations of allergic disorders and can be an additional driver of the allergic diathesis (Galli and Tsai, 2012). STAT6 hyperactivation in airway epithelial cells and resident dendritic cells can further create an environment favoring asthma and chronic lung disease, as this would induce production of chemokines that promote T_H_2 cells and eosinophil recruitment (Matsukura et al., 2001; Medoff et al., 2009). Population genetics provide further support for the central role that STAT6 plays in the development of human allergic disease. Multiple independent genome-wide association studies (GWAS) have found that polymorphisms in *STAT6* associate with many allergic conditions (Table 1). Our study expands this appreciation of the role of STAT6 in human disease by establishing that heterozygous GOF variants cause a monogenic form of severe allergic disease.

**Table 1.** Number of published GWAS studies linking polymorphisms (SNPs) in *STAT6* to common allergic diseases in the population

Phenotype	Number of published associations	References
Asthma^a^	14	Daya et al., 2019; Demenais et al., 2018; Ferreira et al., 2019; Han et al., 2020; Johansson et al., 2019; Olafsdottir et al., 2020; Pickrell et al., 2016; Pividori et al., 2019; Sakaue et al., 2021; Shrine et al., 2019; Valette et al., 2021; Zhu et al., 2020; Zhu et al., 2018; Zhu et al., 2019
Eosinophil count	7	Astle et al., 2016; Chen et al., 2020; Höglund et al., 2022; Kachuri et al., 2021; Kichaev et al., 2019; Sakaue et al., 2021; Vuckovic et al., 2020
Allergic disease	3	Ferreira et al., 2017; Ferreira et al., 2020; Zhu et al., 2018
Atopic dermatitis/eczema	3	Johansson et al., 2019; Kichaev et al., 2019; Tanaka et al., 2021
Serum IgE level	2	Daya et al., 2021; Granada et al., 2012
Allergic sensitization	2	Bønnelykke et al., 2013; Waage et al., 2018
Allergic rhinitis	1	Johansson et al., 2019
Eosinophilic gastrointestinal disorders	1	Sleiman et al., 2014

Significant genome-wide associations (P < 5 × 10^−8^) between STAT6 SNPs and all relevant allergic traits were obtained through the National Human Genome Research Institute–European Bioinformatics Institute GWAS Catalog (https://www.ebi.ac.uk/gwas/).

a Includes asthma, childhood-onset asthma, adult-onset asthma, and atopic asthma.

The fatal cerebral aneurysm in P10 (p.E382Q) was not clinically anticipated, but it is possible that the *STAT6* GOF variant also increased the risk of developing cerebral aneurysms. Indeed, P1 (p.D419G) also had multiple rare anatomical variants in the arteries of the Circle of Willis. Intracranial aneurysms have been reported in patients with both STAT3 loss-of-function (LOF) and STAT1 GOF (Chandesris et al., 2012; Dadak et al., 2017; Toubiana et al., 2016). Increased activation of other STAT family members, including STAT2, STAT3, and STAT5 have also been observed in human abdominal aortic aneurysms (STAT6 was not evaluated), although it is not clear whether enhanced STAT phosphorylation contributes to aneurysms or is the result of inflammation caused by aneurysms (Liao et al., 2012). As more individuals with *STAT6* GOF variants are identified, the possibility of cerebral vascular anomalies warrants investigation.

It is noteworthy that the oldest patient in this cohort, P7 (p.D419H), experienced recurrent B cell lymphoma—follicular lymphoma at 49 yr and diffuse large B cell lymphoma at age 60 yr. Activating somatic mutations in *STAT6* are well documented in B cell lymphoma with amino acid D419 being a particular mutational hotspot (Ritz et al., 2009; Tate et al., 2019; Yildiz et al., 2015). The patient’s p.D419H variant has been reported multiple times as a somatic mutation in COSMIC, as have other variants found in our patient cohort (i.e., p.D419, p.D519, and p.P643). More patients will need to be identified and followed to fully define the phenotype caused by germline *STAT6* GOF variants, but it is biologically plausible that these patients may be at higher risk of developing B cell malignancies warranting enhanced clinical vigilance.

A GOF STAT6 model (designated STAT6VT) has previously been described in vitro (Daniel et al., 2000) and has been used to study chronic atopic dermatitis in mouse models (Bruns et al., 2003; DaSilva-Arnold et al., 2018). STAT6VT has the substitution of two amino acid residues, at positions 547 and 548, in the SH2 domain resulting in a STAT6 mutant that is constitutively active in an IL-4–independent manner and is unresponsive to IL-4 stimulation (Daniel et al., 2000). The humans we identified with STAT6 GOF variants and STAT6VT mice share a number of key features of the allergic diathesis, including elevated serum IgE and chronic atopic dermatitis. Very recently, a report was published describing a father and his two sons with severe allergic disease who were all heterozygous for the GOF *STAT6* variant p.E377K (Suratannon et al., 2022). This new family shares many of the features we report in our cohort of 10 families (Fig. 2 A), further emphasizing that patients with early onset severe allergic disease should be assessed for underlying monogenic gene defects, including STAT6.

There is now a growing list of human single gene defects that cause the classic hyper-IgE phenotypic triad of eczema, recurrent skin and lung infections, and elevated serum IgE (Freeman and Milner, 2020; Vaseghi-Shanjani et al., 2021; Zhang et al., 2018). AD hyper-IgE syndrome caused by dominant negative variants in *STAT3* (i.e., Job’s syndrome or STAT3 LOF) is the best characterized of these conditions, but this list of disorders also includes other AD (*IL6ST*; Beziat et al., 2020) and autosomal recessive (AR; *ZNF341* [Béziat et al., 2018], *IL6ST* [Shahin et al., 2019]) disorders (Bergerson and Freeman, 2019; Minegishi, 2021). Notably, the patients we identified with *STAT6* GOF variants did exhibit some of the extra-immunological features typical of STAT3 LOF, specifically hyperextensible joints, pathologic fractures, and vascular anomalies (Bergerson and Freeman, 2019).

Beyond defining the phenotype of STAT6 GOF, we also present laboratory and clinical evidence supporting the effectiveness of dupilumab and tofacitinib treatment in these patients. It was notable that the three patients (P1, P2, and P6) who received dupilumab have experienced dramatic improved atopic dermatitis and could be weaned off systemic corticosteroids. P6, who had short stature and delayed bone age before starting the biologic agent, experienced rapid height and weight gain following initiation of dupilumab. In addition to the documented clinical benefits of dupilumab therapy in patients with STAT6 GOF, we also present early data suggesting that the JAK inhibitors, ruxolitinib or tocafitinib, may be effective in this patient population.

While this study has many strengths, notably the extreme allergic phenotype of the 16 patients combined with in-depth functional assessment of their *STAT6* variants; because of the global nature of our cohort, the study does have limitations. First, patients were identified by their local expert clinicians as candidates for genetic assessment based on their extreme allergic phenotype and, in some cases, their family history. As a result, we do not have prospectively defined inclusion criteria. Second, the global nature of the cohort and variation in local access to medications such as dupilumab limited our ability to run the same assays on pre-treatment primary cells from all patients. Despite these limitations, our study does identify GOF variants in *STAT6* as a novel monogenic allergic disorder. We also present clinical and single cell evidence of the effectiveness of dupilumab in STAT6 GOF patients. We anticipate that this discovery will facilitate the recognition and targeted treatment of more affected individuals and, with time, a full definition of the human genotype-phenotype relationship caused by germline human *STAT6* GOF variants will emerge, including understanding the risk of lymphoma.

Based on our findings reported in this study, we suggest that heterozygous GOF variants in *STAT6* be added to the list of AD causes of the hyper-IgE phenotype. While each of the conditions known to cause a hyper-IgE phenotype has some specific clinical features (e.g., viral skin infections are a defining feature of DOCK8 deficiency; Biggs et al., 2017; Chu et al., 2012), there is considerable clinical overlap and clinically approved testing of these pathways is rarely available. Therefore, we recommend genetic testing as the most efficient initial diagnostic approach to patients who experience severe allergic disease beginning very early in life. Finally, we demonstrate that dupilumab and JAK inhibition may be an effective targeted treatment options for patients with GOF *STAT6* variants.

## Materials and methods

### Ethical considerations

All procedures performed in the study were in accordance with the ethical standards of the institutional research committee and with the 1964 Helsinki declaration and its later amendments or comparable ethical standards. All study participants and/or their parents/guardians provided written informed consent. Research study protocols were approved by local institutions, specifically: The University of British Columbia Clinical Research Ethics Board (H09-01228, H15-00641), University College London Research Ethics Committee (04/Q0501/119, 06/Q0508/16), University of Hong Kong Institutional Review Board (UW 08-301), National Institutes of Health Institutional Review Board (NCT01164241), Children’s Hospital of Philadelphia Institutional Review Board (19-016617), Children’s Hospital Bambino Gesù Ethical Committee (1702_OPBG_2018).

### Identification of STAT6 variant via next-generation sequencing

Based on local availability, research or clinical next-generation sequencing of the genomic DNA was performed using either whole exome or a targeted panel approach (as described previously; Béziat et al., 2021; Campbell et al., 2022; Chovanec et al., 2022; Hebert et al., 2022; Murrell et al., 2022; Tarailo-Graovac et al., 2016). After bioinformatic analysis, de novo and inherited *STAT6* variants that were predicted to be damaging and that segregated with disease were identified in each family (Tables S4 and S5).

### Generation and expression of STAT6 variant plasmids

*STAT6* variants described in this study were generated through site-directed mutagenesis (SDM) for transfection purposes. Expression of WT STAT6 or STAT6 variants were induced transiently in HEK293 cells using lipofectamine, or stably in Jurkat T cells using a lenti-viral approach. See supplemental methods at the end of the PDF for details.

### Luciferase reporter assays

Luciferase reporter plasmids encoding a 4× STAT6 binding site (TTCCCAAGAA; the underlined bases represent the two half-sites for STAT6-specific binding), encoding the promoter site for *CCL26*, and encoding the promoter site for *FCER2* were used to assess WT and variant STAT6 promoter activity (Li et al., 2016; Yildiz et al., 2015). See supplemental methods at the end of the PDF for details.

### Flow cytometry

(a) Phospho-flow cytometry: STAT6 phosphorylation was determined via phospho-flow cytometry for STAT6-transfected HEK293 cells, peripheral blood mononuclear cells (PBMCs), T cell blasts, and EBV-transformed lymphoblastoid B cell lines (LCLs). (b) Intracellular cytokine staining: Intracellular cytokine staining was conducted on nine patient PBMCs, alongside 15 healthy controls, to study CD4^+^ T helper subsets as previously described (Sharma et al., 2022). (c) CD23 and IL-4Rα expression was studied on seven patient PBMCs and nine healthy control PBMCs. See supplemental methods at the end of the PDF for details.

### Immunoblotting

Immunoblotting was conducted as previously described (Sharma et al., 2022) to assess the phosphorylation status of p.P643R STAT6 variant, as phosphorylation of this variant could not be detected via flow cytometry, using an antibody against the tyrosine 641 phosphorylation site. See supplemental methods at the end of the PDF for details.

### RNAseq

(a) Jurkat cells: To model transcriptomics changes caused by STAT6 variants, Jurkat T cells were transduced with either c.1144G>C, p.E382Q, c.1256A>G, p.D419G, or WT STAT6. The cells were either left unstimulated or stimulated with 100 ng/ml of IL-4 for 4 h and subsequently used for RNA extraction and sequencing. (b) Whole blood: Bulk RNAseq was done on 10 samples: one patient sample before dupilumab treatment initiation, four patient samples after dupilumab treatment initiation, and five healthy controls. (c) scRNAseq: Performed on PBMCs and enriched T cells from the patient sample before and 2 yr after dupilumab treatment, along with one age-matched healthy control. See supplemental methods at the end of the PDF for details.

### Histology

Formalin-fixed, paraffin-embedded gastric, duodenal, and skin tissue were sectioned and subjected to H&E staining.

### Structural modeling

The effects of the STAT6 variants on the protein function and structure were analyzed using three-dimensional models. SWISS-MODEL (Waterhouse et al., 2018) was used to model the variants based on a template structure of the human STAT6 transcription factor solved as a homodimer and in complex with DNA (PDB: 4Y5W, resolution: 3.1 Å, chains A, C, M, and N; Li et al., 2016). Structures were visualized with UCSF Chimera (Pettersen et al., 2004).

### Online supplemental material

Clinical narratives for each patient are presented as Data S1. Fig. S1 is a detailed structural model showing the DNA and STAT6 variant interface. Fig. S2 shows complete blood count and the immunological workup of all the patients. Fig. S3 shows additional in vitro data confirming the GOF nature of the STAT6 variants. Further workup of the primary patient cells is shown in Fig. S4. Additional IL4Rα antibody and JAK inhibitor treatment data of cells and patients is presented in Fig. S5. Table S1 lists primers used for site-directed mutagenesis. Table S2 lists antibodies used for phospho-flow on different immune subsets. Table S3 lists antibodies used for T_H_ phenotyping in patient PBMCs. Pathogenicity prediction of the variants are presented in Tables S4 and S5. Tables S6 and S7 are gene lists from GSEA analysis shown in Fig. 3. Supplemental methods are listed at the end of the PDF.

## Supplementary Material

Table S1 lists primers used for site-directed mutagenesis.

Table S2 lists antibodies used for phospho-flow on different immune subsets.

Table S3 lists antibodies used for T_H_ phenotyping in patient PBMCs.

Table S4 shows variant annotation and pathogenicity prediction of the variants reported within the DNA-binding domain of STAT6 for 10 patients.

Table S5 shows variant annotation and pathogenicity prediction of the variants reported outside of the DNA-binding domain of STAT6 for six patients.

Table S6 lists genes upregulated (gray) and downregulated (red) in transduced WT-, p.E382Q-, and p.D419G-transduced Jurkats that meet the cutoff of fold-change (FC) > 1.25 and adjusted P value <0.05.

Table S7 lists genes in the leading edge driving the enrichment of two pathways (IL-4/STAT6 pathway and TH2 pathway) between transduced WT- vs. p.E382Q- and WT- vs. p.D419G- Jurkats

Data S1 provides clinical narratives for each patient.

SourceData F3 contains original blots for Fig. 3.

## References

[bib1] Adzhubei, I.A., S. Schmidt, L. Peshkin, V.E. Ramensky, A. Gerasimova, P. Bork, A.S. Kondrashov, and S.R. Sunyaev. 2010. A method and server for predicting damaging missense mutations. Nat. Methods. 7:248–249. 10.1038/nmeth0410-248 20354512 PMC2855889

[bib2] Aran, D., A.P. Looney, L. Liu, E. Wu, V. Fong, A. Hsu, S. Chak, R.P. Naikawadi, P.J. Wolters, A.R. Abate, . 2019. Reference-based analysis of lung single-cell sequencing reveals a transitional profibrotic macrophage. Nat. Immunol. 20:163–172. 10.1038/s41590-018-0276-y 30643263 PMC6340744

[bib3] Astle, W.J., H. Elding, T. Jiang, D. Allen, D. Ruklisa, A.L. Mann, D. Mead, H. Bouman, F. Riveros-Mckay, M.A. Kostadima, . 2016. The allelic landscape of human blood cell trait variation and links to common complex disease. Cell. 167:1415–1429.e19. 10.1016/j.cell.2016.10.042 27863252 PMC5300907

[bib4] Bacharier, L.B., J.F. Maspero, C.H. Katelaris, A.G. Fiocchi, R. Gagnon, I. de Mir, N. Jain, L.D. Sher, X. Mao, and D. Liu, . 2021. Dupilumab in children with uncontrolled moderate-to-severe asthma. N. Engl. J. Med. 385:2230–2240. 10.1056/NEJMoa2106567 34879449

[bib5] Beck, L.A., D. Thaçi, J.D. Hamilton, N.M. Graham, T. Bieber, R. Rocklin, J.E. Ming, H. Ren, R. Kao, E. Simpson, . 2014. Dupilumab treatment in adults with moderate-to-severe atopic dermatitis. N. Engl. J. Med. 371:130–139. 10.1056/NEJMoa1314768 25006719

[bib6] Bergerson, J.R.E., and A.F. Freeman. 2019. An update on syndromes with a hyper-IgE phenotype. Immunol. Allergy Clin. North Am. 39:49–61. 10.1016/j.iac.2018.08.007 30466772

[bib7] Bettigole, S.E., R. Lis, S. Adoro, A.H. Lee, L.A. Spencer, P.F. Weller, and L.H. Glimcher. 2015. The transcription factor XBP1 is selectively required for eosinophil differentiation. Nat. Immunol. 16:829–837. 10.1038/ni.3225 26147683 PMC4577297

[bib8] Béziat, V., J. Li, J.X. Lin, C.S. Ma, P. Li, A. Bousfiha, I. Pellier, S. Zoghi, S. Baris, S. Keles, . 2018. A recessive form of hyper-IgE syndrome by disruption of ZNF341-dependent STAT3 transcription and activity. Sci. Immunol. 3:3. 10.1126/sciimmunol.aat4956 PMC6141026 29907691

[bib9] Béziat, V., F. Rapaport, J. Hu, M. Titeux, M. Bonnet des Claustres, M. Bourgey, H. Griffin, É. Bandet, C.S. Ma, R. Sherkat, . 2021. Humans with inherited T cell CD28 deficiency are susceptible to skin papillomaviruses but are otherwise healthy. Cell. 184:3812–3828.e30. 10.1016/j.cell.2021.06.004 34214472 PMC8329841

[bib10] Beziat, V., S.J. Tavernier, Y.H. Chen, C.S. Ma, M. Materna, A. Laurence, J. Staal, D. Aschenbrenner, L. Roels, L. Worley, . 2020. Dominant-negative mutations in human IL6ST underlie hyper-IgE syndrome. J. Exp. Med. 217:e20191804. 10.1084/jem.20191804 32207811 PMC7971136

[bib11] Biggs, C.M., S. Keles, and T.A. Chatila. 2017. DOCK8 deficiency: Insights into pathophysiology, clinical features and management. Clin. Immunol. 181:75–82. 10.1016/j.clim.2017.06.003 28625885 PMC5555255

[bib12] Bissonnette, R., K.A. Papp, Y. Poulin, M. Gooderham, M. Raman, L. Mallbris, C. Wang, V. Purohit, C. Mamolo, J. Papacharalambous, and W.C. Ports. 2016. Topical tofacitinib for atopic dermatitis: A phase IIa randomized trial. Br. J. Dermatol. 175:902–911. 10.1111/bjd.14871 27423107

[bib13] Bolton, S.M., A.F. Kagalwalla, and J.B. Wechsler. 2018. Eosinophilic esophagitis in children: Endoscopic findings at diagnosis and post-intervention. Curr. Gastroenterol. Rep. 20:4. 10.1007/s11894-018-0607-z 29492720 PMC6448395

[bib14] Bønnelykke, K., M.C. Matheson, T.H. Pers, R. Granell, D.P. Strachan, A.C. Alves, A. Linneberg, J.A. Curtin, N.M. Warrington, and M. Standl, . 2013. Meta-analysis of genome-wide association studies identifies ten loci influencing allergic sensitization. Nat. Genet. 45:902–906. 10.1038/ng.2694 23817571 PMC4922420

[bib15] Bruns, H.A., U. Schindler, and M.H. Kaplan. 2003. Expression of a constitutively active Stat6 in vivo alters lymphocyte homeostasis with distinct effects in T and B cells. J. Immunol. 170:3478–3487. 10.4049/jimmunol.170.7.3478 12646608

[bib16] Campbell, T.M., Z. Liu, Q. Zhang, M. Moncada-Velez, L.E. Covill, P. Zhang, I. Alavi Darazam, P. Bastard, L. Bizien, G. Bucciol, . 2022. Respiratory viral infections in otherwise healthy humans with inherited IRF7 deficiency. J. Exp. Med. 219:e20220202 35670811 10.1084/jem.20220202 PMC9178406

[bib17] Chandesris, M.O., A. Azarine, K.T. Ong, S. Taleb, P. Boutouyrie, E. Mousseaux, M. Romain, E. Bozec, S. Laurent, N. Boddaert, . 2012. Frequent and widespread vascular abnormalities in human signal transducer and activator of transcription 3 deficiency. Circ. Cardiovasc. Genet. 5:25–34. 10.1161/CIRCGENETICS.111.961235 22084479

[bib18] Chen, M.-H., L.M. Raffield, A. Mousas, S. Sakaue, J.E. Huffman, A. Moscati, B. Trivedi, T. Jiang, P. Akbari, and D. Vuckovic, . 2020. Trans-ethnic and ancestry-specific blood-cell genetics in 746,667 individuals from 5 global populations. Cell. 182:1198–1213.e14. 10.1016/j.cell.2020.06.045 32888493 PMC7480402

[bib19] Chen, Y., A.T. Lun, and G.K. Smyth. 2016. From reads to genes to pathways: Differential expression analysis of RNA-seq experiments using rsubread and the edgeR quasi-likelihood pipeline. F1000 Res. 5:1438 10.12688/f1000research.8987.1 PMC4934518 27508061

[bib20] Chovanec, J., I. Tunc, J. Hughes, J. Halstead, A. Mateja, Y. Liu, M. O’Connell, J. Kim, Y.H. Park, Q. Wang, . 2022. Genetically determining individualized clinical reference ranges for the biomarker tryptase can limit unnecessary procedures and unmask myeloid neoplasms. Blood Adv.:bloodadvances.2022007936. 10.1182/bloodadvances.2022007936 36170795 PMC10164828

[bib21] Chu, E.Y., A.F. Freeman, H. Jing, E.W. Cowen, J. Davis, H.C. Su, S.M. Holland, and M.L. Turner. 2012. Cutaneous manifestations of DOCK8 deficiency syndrome. Arch. Dermatol. 148:79–84. 10.1001/archdermatol.2011.262 21931011 PMC4103903

[bib22] Dadak, M., R. Jacobs, J. Skuljec, A.C. Jirmo, Ö. Yildiz, F. Donnerstag, N.T. Baerlecken, R.E. Schmidt, H. Lanfermann, T. Skripuletz, . 2017. Gain-of-function STAT1 mutations are associated with intracranial aneurysms. Clin. Immunol. 178:79–85. 10.1016/j.clim.2017.01.012 28161409

[bib23] Daniel, C., A. Salvekar, and U. Schindler. 2000. A gain-of-function mutation in STAT6. J. Biol. Chem. 275:14255–14259. 10.1074/jbc.C000129200 10747856

[bib24] DaSilva-Arnold, S.C., A. Thyagarajan, L.J. Seymour, Q. Yi, J.R. Bradish, M. Al-Hassani, H. Zhou, N.J. Perdue, V. Nemeth, A. Krbanjevic, . 2018. Phenotyping acute and chronic atopic dermatitis-like lesions in Stat6VT mice identifies a role for IL-33 in disease pathogenesis. Arch. Dermatol. Res. 310:197–207. 10.1007/s00403-018-1807-y 29368135 PMC6198812

[bib25] Daya, M., C. Cox, N. Acevedo, M.P. Boorgula, M. Campbell, S. Chavan, M.H. Cho, G.L. David, P. Kachroo, and J. Lasky-Su, . 2021. Multiethnic genome-wide and HLA association study of total serum IgE level. J. Allergy Clin. Immunol. 148:1589–1595. 10.1016/j.jaci.2021.09.011 34536413 PMC8665111

[bib26] Daya, M., N. Rafaels, T.M. Brunetti, S. Chavan, A.M. Levin, A. Shetty, C.R. Gignoux, M.P. Boorgula, G. Wojcik, and M. Campbell, . 2019. Association study in African-admixed populations across the Americas recapitulates asthma risk loci in non-African populations. Nat. Commun. 10:880. 10.1038/s41467-019-08469-7 30787307 PMC6382865

[bib27] Demenais, F., P. Margaritte-Jeannin, K.C. Barnes, W.O.C. Cookson, J. Altmüller, W. Ang, R.G. Barr, T.H. Beaty, A.B. Becker, and J. Beilby, . 2018. Multiancestry association study identifies new asthma risk loci that colocalize with immune-cell enhancer marks. Nat. Genet. 50:42–53. 10.1038/s41588-017-0014-7 29273806 PMC5901974

[bib28] Dierick, B.J.H., T. van der Molen, B.M.J. Flokstra-de Blok, A. Muraro, M.J. Postma, J.W.H. Kocks, and J.F.M. van Boven. 2020. Burden and socioeconomics of asthma, allergic rhinitis, atopic dermatitis and food allergy. Expert Rev. Pharmacoecon. Outcomes Res. 20:437–453. 10.1080/14737167.2020.1819793 32902346

[bib29] Elo, L.L., H. Järvenpää, S. Tuomela, S. Raghav, H. Ahlfors, K. Laurila, B. Gupta, R.J. Lund, J. Tahvanainen, R.D. Hawkins, . 2010. Genome-wide profiling of interleukin-4 and STAT6 transcription factor regulation of human Th2 cell programming. Immunity. 32:852–862. 10.1016/j.immuni.2010.06.011 20620947

[bib30] Ferreira, M.A., J.M. Vonk, H. Baurecht, I. Marenholz, C. Tian, J.D. Hoffman, Q. Helmer, A. Tillander, V. Ullemar, and J. van Dongen, . 2017. Shared genetic origin of asthma, hay fever and eczema elucidates allergic disease biology. Nat. Genet. 49:1752–1757. 10.1038/ng.3985 29083406 PMC5989923

[bib31] Ferreira, M.A.R., R. Mathur, J.M. Vonk, A. Szwajda, B. Brumpton, R. Granell, B.K. Brew, V. Ullemar, Y. Lu, and Y. Jiang, . 2019. Genetic architectures of childhood- and adult-onset asthma are partly distinct. Am. J. Hum. Genet. 104:665–684. 10.1016/j.ajhg.2019.02.022 30929738 PMC6451732

[bib32] Ferreira, M.A.R., J.M. Vonk, H. Baurecht, I. Marenholz, C. Tian, J.D. Hoffman, Q. Helmer, A. Tillander, V. Ullemar, and Y. Lu, . 2020. Age-of-onset information helps identify 76 genetic variants associated with allergic disease. PLoS Genet. 16:e1008725. 10.1371/journal.pgen.1008725 32603359 PMC7367489

[bib33] Freeman, A.F., and J.D. Milner. 2020. The child with elevated IgE and infection susceptibility. Curr. Allergy Asthma Rep. 20:65. 10.1007/s11882-020-00964-y 32830295

[bib34] Fung, S.Y., H.Y. Lu, M. Sharma, A.A. Sharma, A. Saferali, A. Jia, L. Abraham, T. Klein, M.R. Gold, L.D. Noterangelo, . 2021. MALT1-Dependent cleavage of HOIL1 modulates canonical NF-κB signaling and inflammatory responsiveness. Front. Immunol. 12:749794. 10.3389/fimmu.2021.749794 34721419 PMC8552041

[bib35] Galli, S.J., and M. Tsai. 2012. IgE and mast cells in allergic disease. Nat. Med. 18:693–704. 10.1038/nm.2755 22561833 PMC3597223

[bib36] Ginhoux, F., J.L. Schultze, P.J. Murray, J. Ochando, and S.K. Biswas. 2016. New insights into the multidimensional concept of macrophage ontogeny, activation and function. Nat. Immunol. 17:34–40. 10.1038/ni.3324 26681460

[bib37] Goenka, S., and M.H. Kaplan. 2011. Transcriptional regulation by STAT6. Immunol. Res. 50:87–96. 10.1007/s12026-011-8205-2 21442426 PMC3107597

[bib38] Gowthaman, U., J.S. Chen, B. Zhang, W.F. Flynn, Y. Lu, W. Song, J. Joseph, J.A. Gertie, L. Xu, M.A. Collet, . 2019. Identification of a T follicular helper cell subset that drives anaphylactic IgE. Science. 365:365. 10.1126/science.aaw6433 PMC6901029 31371561

[bib39] Granada, M., J.B. Wilk, M. Tuzova, D.P. Strachan, S. Weidinger, E. Albrecht, C. Gieger, J. Heinrich, B.E. Himes, G.M. Hunninghake, . 2012. A genome-wide association study of plasma total IgE concentrations in the Framingham Heart Study. J. Allergy Clin. Immunol. 129:840–845.e21. 10.1016/j.jaci.2011.09.029 22075330 PMC3293994

[bib40] Hafemeister, C., and R. Satija. 2019. Normalization and variance stabilization of single-cell RNA-seq data using regularized negative binomial regression. Genome Biol. 20:296. 10.1186/s13059-019-1874-1 31870423 PMC6927181

[bib41] Han, Y., Q. Jia, P.S. Jahani, B.P. Hurrell, C. Pan, P. Huang, J. Gukasyan, N.C. Woodward, E. Eskin, F.D. Gilliland, . 2020. Genome-wide analysis highlights contribution of immune system pathways to the genetic architecture of asthma. Nat. Commun. 11:1776. 10.1038/s41467-020-15649-3 32296059 PMC7160128

[bib42] Hebert, A., A. Simons, J.H.M. Schuurs-Hoeijmakers, H.J.P.M. Koenen, E. Zonneveld-Huijssoon, S.S.V. Henriet, E.J.H. Schatorjé, E.P.A.H. Hoppenreijs, E.K.S.M. Leenders, E.J.M. Janssen, . 2022. Trio-based whole exome sequencing in patients with suspected sporadic inborn errors of immunity: A retrospective cohort study. Elife. 11:e78469. 10.7554/eLife.78469 36250618 PMC9635875

[bib43] Höglund, J., F. Hadizadeh, W.E. Ek, T. Karlsson, and Å. Johansson. 2022. Gene-based variant analysis of whole-exome sequencing in relation to eosinophil count. Front. Immunol. 13:862255. 10.3389/fimmu.2022.862255 35935937 PMC9355086

[bib44] Johansson, Å., M. Rask-Andersen, T. Karlsson, and W.E. Ek. 2019. Genome-wide association analysis of 350 000 Caucasians from the UK Biobank identifies novel loci for asthma, hay fever and eczema. Hum. Mol. Genet. 28:4022–4041. 10.1093/hmg/ddz175 31361310 PMC6969355

[bib45] Kachuri, L., S. Jeon, A.T. DeWan, C. Metayer, X. Ma, J.S. Witte, C.W.K. Chiang, J.L. Wiemels, and A.J. de Smith. 2021. Genetic determinants of blood-cell traits influence susceptibility to childhood acute lymphoblastic leukemia. Am. J. Hum. Genet. 108:1823–1835. 10.1016/j.ajhg.2021.08.004 34469753 PMC8546033

[bib46] Kaplan, M.H., U. Schindler, S.T. Smiley, and M.J. Grusby. 1996. Stat6 is required for mediating responses to IL-4 and for development of Th2 cells. Immunity. 4:313–319. 10.1016/S1074-7613(00)80439-2 8624821

[bib47] Kichaev, G., G. Bhatia, P.-R. Loh, S. Gazal, K. Burch, M.K. Freund, A. Schoech, B. Pasaniuc, and A.L. Price. 2019. Leveraging polygenic functional enrichment to improve GWAS power. Am. J. Hum. Genet. 104:65–75. 10.1016/j.ajhg.2018.11.008 30595370 PMC6323418

[bib48] Kim, B.S., M.D. Howell, K. Sun, K. Papp, A. Nasir, M.E. Kuligowski, I.S. Investigators, and INCB 18424-206 Study Investigators. 2020. Treatment of atopic dermatitis with ruxolitinib cream (JAK1/JAK2 inhibitor) or triamcinolone cream. J. Allergy Clin. Immunol. 145:572–582. 10.1016/j.jaci.2019.08.042 31629805

[bib49] Kim, Y., E.K. Kwon, J.H. Jeon, I. So, I.G. Kim, M.S. Choi, I.S. Kim, J.K. Choi, J.U. Jung, and N.H. Cho. 2012. Activation of the STAT6 transcription factor in Jurkat T-cells by the herpesvirus saimiri Tip protein. J. Gen. Virol. 93:330–340. 10.1099/vir.0.036087-0 22012462 PMC3352339

[bib50] Kneitz, C., M. Goller, R. Seggewiss, A. Yaman, E. Serfling, and H.P. Tony. 2000. STAT6 and the regulation of CD23 expression in B-chronic lymphocytic leukemia. Leuk. Res. 24:331–337. 10.1016/S0145-2126(99)00191-5 10713330

[bib51] Kulpa, D.A., A. Talla, J.H. Brehm, S.P. Ribeiro, S. Yuan, A.G. Bebin-Blackwell, M. Miller, R. Barnard, S.G. Deeks, D. Hazuda, . 2019. Differentiation into an effector memory phenotype potentiates HIV-1 latency reversal in CD4^+^ T cells. J. Virol. 93:93. 10.1128/JVI.00969-19 PMC6880164 31578289

[bib52] Kutner, R.H., X.Y. Zhang, and J. Reiser. 2009. Production, concentration and titration of pseudotyped HIV-1-based lentiviral vectors. Nat. Protoc. 4:495–505. 10.1038/nprot.2009.22 19300443

[bib105] Lemonnier, N., E. Melén, Y. Jiang, S. Joly, C. Ménard, D. Aguilar, E. Acosta-Perez, A. Bergström, N. Boutaoui, M. Bustamante, . 2020. A novel whole blood gene expression signature for asthma, dermatitis, and rhinitis multimorbidity in children and adolescents. Allergy. 75:3248–3260. 10.1111/all.14314 32277847 PMC9302020

[bib53] Li, J., J.P. Rodriguez, F. Niu, M. Pu, J. Wang, L.W. Hung, Q. Shao, Y. Zhu, W. Ding, Y. Liu, . 2016. Structural basis for DNA recognition by STAT6. Proc. Natl. Acad. Sci. USA. 113:13015–13020. 10.1073/pnas.1611228113 27803324 PMC5135355

[bib54] Liao, M., J. Xu, A.J. Clair, B. Ehrman, L.M. Graham, and M.J. Eagleton. 2012. Local and systemic alterations in signal transducers and activators of transcription (STAT) associated with human abdominal aortic aneurysms. J. Surg. Res. 176:321–328. 10.1016/j.jss.2011.05.041 21764069 PMC3197955

[bib55] Lu, H.Y., M. Sharma, A.A. Sharma, A. Lacson, A. Szpurko, J. Luider, P. Dharmani-Khan, A. Shameli, P.A. Bell, G.M.T. Guilcher, . 2021. Mechanistic understanding of the combined immunodeficiency in complete human CARD11 deficiency. J. Allergy Clin. Immunol. 148:1559–1574.e13. 10.1016/j.jaci.2021.04.006 33872653

[bib56] Lundberg, M., A. Eriksson, B. Tran, E. Assarsson, and S. Fredriksson. 2011. Homogeneous antibody-based proximity extension assays provide sensitive and specific detection of low-abundant proteins in human blood. Nucleic Acids Res. 39:e102. 10.1093/nar/gkr424 21646338 PMC3159481

[bib57] Lyons, J.J., and J.D. Milner. 2018. Primary atopic disorders. J. Exp. Med. 215:1009–1022. 10.1084/jem.20172306 29549114 PMC5881472

[bib58] Matsukura, S., C. Stellato, S.N. Georas, V. Casolaro, J.R. Plitt, K. Miura, S. Kurosawa, U. Schindler, and R.P. Schleimer. 2001. Interleukin-13 upregulates eotaxin expression in airway epithelial cells by a STAT6-dependent mechanism. Am. J. Respir. Cell Mol. Biol. 24:755–761. 10.1165/ajrcmb.24.6.4351 11415942

[bib59] Medoff, B.D., E. Seung, S. Hong, S.Y. Thomas, B.P. Sandall, J.S. Duffield, D.A. Kuperman, D.J. Erle, and A.D. Luster. 2009. CD11b^+^ myeloid cells are the key mediators of Th2 cell homing into the airway in allergic inflammation. J. Immunol. 182:623–635. 10.4049/jimmunol.182.1.623 19109196 PMC2718444

[bib60] Mikita, T., D. Campbell, P. Wu, K. Williamson, and U. Schindler. 1996. Requirements for interleukin-4-induced gene expression and functional characterization of Stat6. Mol. Cell. Biol. 16:5811–5820. 10.1128/MCB.16.10.5811 8816495 PMC231582

[bib61] Milner, J.D. 2020. Primary atopic disorders. Annu. Rev. Immunol. 38:785–808. 10.1146/annurev-immunol-042718-041553 32126183

[bib62] Minegishi, Y. 2021. Hyper-IgE syndrome, 2021 update. Allergol. Int. 70:407–414. 10.1016/j.alit.2021.07.007 34419355

[bib63] Monaco, G., B. Lee, W. Xu, S. Mustafah, Y.Y. Hwang, C. Carre, N. Burdin, L. Visan, M. Ceccarelli, M. Poidinger, . 2019. RNA-seq signatures normalized by mRNA abundance allow absolute deconvolution of human immune cell types. Cell Rep. 26:1627–1640 e1627. 10.1016/j.celrep.2019.01.041 30726743 PMC6367568

[bib64] Murrell, J.R., A.M.I. Nesbitt, S.W. Baker, K.B. Pechter, J. Balciuniene, X. Zhao, E.H. Denenberg, E.T. DeChene, C. Wu, P. Jayaraman, . 2022. Molecular diagnostic outcomes from 700 cases: What can we learn from a retrospective analysis of clinical exome sequencing? J. Mol. Diagn. 24:274–286. 10.1016/j.jmoldx.2021.12.002 35065284 PMC9904168

[bib65] Newman, A.M., C.L. Liu, M.R. Green, A.J. Gentles, W. Feng, Y. Xu, C.D. Hoang, M. Diehn, and A.A. Alizadeh. 2015. Robust enumeration of cell subsets from tissue expression profiles. Nat. Methods. 12:453–457. 10.1038/nmeth.3337 25822800 PMC4739640

[bib66] Novershtern, N., A. Subramanian, L.N. Lawton, R.H. Mak, W.N. Haining, M.E. McConkey, N. Habib, N. Yosef, C.Y. Chang, T. Shay, . 2011. Densely interconnected transcriptional circuits control cell states in human hematopoiesis. Cell. 144:296–309. 10.1016/j.cell.2011.01.004 21241896 PMC3049864

[bib67] Olafsdottir, T.A., F. Theodors, K. Bjarnadottir, U.S. Bjornsdottir, A.B. Agustsdottir, O.A. Stefansson, E.V. Ivarsdottir, J.K. Sigurdsson, S. Benonisdottir, G.I. Eyjolfsson, . 2020. Eighty-eight variants highlight the role of T cell regulation and airway remodeling in asthma pathogenesis. Nat. Commun. 11:393. 10.1038/s41467-019-14144-8 31959851 PMC6971247

[bib68] Pettersen, E.F., T.D. Goddard, C.C. Huang, G.S. Couch, D.M. Greenblatt, E.C. Meng, and T.E. Ferrin. 2004. UCSF Chimera--a visualization system for exploratory research and analysis. J. Comput. Chem. 25:1605–1612. 10.1002/jcc.20084 15264254

[bib69] Pickrell, J.K., T. Berisa, J.Z. Liu, L. Ségurel, J.Y. Tung, and D.A. Hinds. 2016. Detection and interpretation of shared genetic influences on 42 human traits. Nat. Genet. 48:709–717. 10.1038/ng.3570 27182965 PMC5207801

[bib70] Pividori, M., N. Schoettler, D.L. Nicolae, C. Ober, and H.K. Im. 2019. Shared and distinct genetic risk factors for childhood-onset and adult-onset asthma: Genome-wide and transcriptome-wide studies. Lancet Respir. Med. 7:509–522. 10.1016/S2213-2600(19)30055-4 31036433 PMC6534440

[bib71] Rapaport, F., B. Boisson, A. Gregor, V. Béziat, S. Boisson-Dupuis, J. Bustamante, E. Jouanguy, A. Puel, J. Rosain, Q. Zhang, . 2021. Negative selection on human genes underlying inborn errors depends on disease outcome and both the mode and mechanism of inheritance. Proc. Natl. Acad. Sci. USA. 118:118. 10.1073/pnas.2001248118 PMC7826345 33408250

[bib72] Rentzsch, P., M. Schubach, J. Shendure, and M. Kircher. 2021. CADD-Splice-improving genome-wide variant effect prediction using deep learning-derived splice scores. Genome Med. 13:31. 10.1186/s13073-021-00835-9 33618777 PMC7901104

[bib73] Ritchie, M.E., B. Phipson, D. Wu, Y. Hu, C.W. Law, W. Shi, and G.K. Smyth. 2015. Limma powers differential expression analyses for RNA-sequencing and microarray studies. Nucleic Acids Res. 43:e47. 10.1093/nar/gkv007 25605792 PMC4402510

[bib74] Ritz, O., C. Guiter, F. Castellano, K. Dorsch, J. Melzner, J.P. Jais, G. Dubois, P. Gaulard, P. Möller, and K. Leroy. 2009. Recurrent mutations of the STAT6 DNA binding domain in primary mediastinal B-cell lymphoma. Blood. 114:1236–1242. 10.1182/blood-2009-03-209759 19423726 PMC2824656

[bib75] Robinson, M.D., and G.K. Smyth. 2007. Moderated statistical tests for assessing differences in tag abundance. Bioinformatics. 23:2881–2887. 10.1093/bioinformatics/btm453 17881408

[bib76] Sakaue, S., M. Kanai, Y. Tanigawa, J. Karjalainen, M. Kurki, S. Koshiba, A. Narita, T. Konuma, K. Yamamoto, and M. Akiyama, . 2021. A cross-population atlas of genetic associations for 220 human phenotypes. Nat. Genet. 53:1415–1424. 10.1038/s41588-021-00931-x 34594039 PMC12208603

[bib77] Shahin, T., D. Aschenbrenner, D. Cagdas, S.K. Bal, C.D. Conde, W. Garncarz, D. Medgyesi, T. Schwerd, B. Karaatmaca, P.G. Cetinkaya, . 2019. Selective loss of function variants in cause Hyper-IgE syndrome with distinct impairments of T-cell phenotype and function. Haematologica. 104:609–621. 10.3324/haematol.2018.194233 30309848 PMC6395342

[bib78] Sharma, M., M.P. Fu, H.Y. Lu, A.A. Sharma, B.P. Modi, C. Michalski, S. Lin, J. Dalmann, A. Salman, K.L. Del Bel, . 2022. Human complete NFAT1 deficiency causes a triad of joint contractures, osteochondromas, and B-cell malignancy. Blood. 140:1858–1874. 10.1182/blood.2022015674 35789258

[bib79] Shimoda, K., J. van Deursen, M.Y. Sangster, S.R. Sarawar, R.T. Carson, R.A. Tripp, C. Chu, F.W. Quelle, T. Nosaka, D.A. Vignali, . 1996. Lack of IL-4-induced Th2 response and IgE class switching in mice with disrupted Stat6 gene. Nature. 380:630–633. 10.1038/380630a0 8602264

[bib80] Shrine, N., M.A. Portelli, C. John, M. Soler Artigas, N. Bennett, R. Hall, J. Lewis, A.P. Henry, C.K. Billington, A. Ahmad, . 2019. Moderate-to-severe asthma in individuals of European ancestry: A genome-wide association study. Lancet Respir. Med. 7:20–34. 10.1016/S2213-2600(18)30389-8 30552067 PMC6314966

[bib81] Sim, N.L., P. Kumar, J. Hu, S. Henikoff, G. Schneider, and P.C. Ng. 2012. SIFT web server: Predicting effects of amino acid substitutions on proteins. Nucleic Acids Res. 40:W452–W457. 10.1093/nar/gks539 22689647 PMC3394338

[bib82] Sleiman, P.M.A., M.-L. Wang, A. Cianferoni, S. Aceves, N. Gonsalves, K. Nadeau, A.J. Bredenoord, G.T. Furuta, J.M. Spergel, and H. Hakonarson. 2014. GWAS identifies four novel eosinophilic esophagitis loci. Nat. Commun. 5:5593. 10.1038/ncomms6593 25407941 PMC4238044

[bib83] Sloka, S., C. Silva, J. Wang, and V.W. Yong. 2011. Predominance of Th2 polarization by vitamin D through a STAT6-dependent mechanism. J. Neuroinflammation. 8:56. 10.1186/1742-2094-8-56 21605467 PMC3118349

[bib84] Sulczewski, F.B., L.A. Martino, B. da Silva Almeida, M.M. Yamamoto, D.S. Rosa, and S.B. Boscardin. 2021. STAT6 signaling pathway controls germinal center responses promoted after antigen targeting to conventional type 2 dendritic cells. Curr. Res. Immunol. 2:120–131. 10.1016/j.crimmu.2021.08.001 35492396 PMC9040147

[bib85] Suratannon, N., C. Ittiwut, W.A. Dik, R. Ittiwut, K. Meesilpavikkai, N. Israsena, P. Ingrungruanglert, V.A.S.H. Dalm, P.L.A. van Daele, A. Sanpavat, . 2022. A germline STAT6 gain-of-function variant is associated with early-onset allergies. J. Allergy Clin. Immunol. 151:565–571.e9 36216080 10.1016/j.jaci.2022.09.028

[bib86] Takeda, K., T. Tanaka, W. Shi, M. Matsumoto, M. Minami, S. Kashiwamura, K. Nakanishi, N. Yoshida, T. Kishimoto, and S. Akira. 1996. Essential role of Stat6 in IL-4 signalling. Nature. 380:627–630. 10.1038/380627a0 8602263

[bib87] Tanaka, N., M. Koido, A. Suzuki, N. Otomo, H. Suetsugu, Y. Kochi, K. Tomizuka, Y. Momozawa, Y. Kamatani, and S. Ikegawa, . 2021. Eight novel susceptibility loci and putative causal variants in atopic dermatitis. J. Allergy Clin. Immunol. 148:1293–1306. 10.1016/j.jaci.2021.04.019 34116867

[bib88] Tarailo-Graovac, M., C. Shyr, C.J. Ross, G.A. Horvath, R. Salvarinova, X.C. Ye, L.H. Zhang, A.P. Bhavsar, J.J. Lee, B.I. Drögemöller, . 2016. Exome sequencing and the management of neurometabolic disorders. N. Engl. J. Med. 374:2246–2255. 10.1056/NEJMoa1515792 27276562 PMC4983272

[bib89] Tate, J.G., S. Bamford, H.C. Jubb, Z. Sondka, D.M. Beare, N. Bindal, H. Boutselakis, C.G. Cole, C. Creatore, E. Dawson, . 2019. COSMIC: The Catalogue of somatic mutations in cancer. Nucleic Acids Res. 47:D941–D947. 10.1093/nar/gky1015 30371878 PMC6323903

[bib90] Toubiana, J., S. Okada, J. Hiller, M. Oleastro, M. Lagos Gomez, J.C. Aldave Becerra, M. Ouachée-Chardin, F. Fouyssac, K.M. Girisha, and A. Etzioni, . 2016. Heterozygous STAT1 gain-of-function mutations underlie an unexpectedly broad clinical phenotype. Blood. 127:3154–3164. 10.1182/blood-2015-11-679902 27114460 PMC4920021

[bib91] Valette, K., Z. Li, V. Bon-Baret, A. Chignon, J.-C. Bérubé, A. Eslami, J. Lamothe, N. Gaudreault, P. Joubert, M. Obeidat, . 2021. Prioritization of candidate causal genes for asthma in susceptibility loci derived from UK Biobank. Commun. Biol. 4:700. 10.1038/s42003-021-02227-6 34103634 PMC8187656

[bib92] Vaseghi-Shanjani, M., K.L. Smith, R.J. Sara, B.P. Modi, A. Branch, M. Sharma, H.Y. Lu, E.L. James, K.J. Hildebrand, C.M. Biggs, and S.E. Turvey. 2021. Inborn errors of immunity manifesting as atopic disorders. J. Allergy Clin. Immunol. 148:1130–1139. 10.1016/j.jaci.2021.08.008 34428518

[bib93] Villarino, A.V., M. Gadina, J.J. O'Shea, and Y. Kanno. 2020. SnapShot: Jak-STAT signaling II. Cell. 181:1696–1696.e1691. 10.1016/j.cell.2020.04.052 32589961 PMC11533724

[bib94] Villarino, A.V., Y. Kanno, and J.J. O’Shea. 2017. Mechanisms and consequences of Jak-STAT signaling in the immune system. Nat. Immunol. 18:374–384. 10.1038/ni.3691 28323260 PMC11565648

[bib95] Vuckovic, D., E.L. Bao, P. Akbari, C.A. Lareau, A. Mousas, T. Jiang, M.-H. Chen, L.M. Raffield, M. Tardaguila, and J.E. Huffman, . 2020. The polygenic and monogenic basis of blood traits and diseases. Cell. 182:1214–1231.e11. 10.1016/j.cell.2020.08.008 32888494 PMC7482360

[bib96] Waage, J., M. Standl, J.A. Curtin, L.E. Jessen, J. Thorsen, C. Tian, N. Schoettler, C. Flores, A. Abdellaoui, and T.S. Ahluwalia, . 2018. Genome-wide association and HLA fine-mapping studies identify risk loci and genetic pathways underlying allergic rhinitis. Nat. Genet. 50:1072–1080. 10.1038/s41588-018-0157-1 30013184 PMC7068780

[bib97] Waterhouse, A., M. Bertoni, S. Bienert, G. Studer, G. Tauriello, R. Gumienny, F.T. Heer, T.A.P. de Beer, C. Rempfer, L. Bordoli, . 2018. SWISS-MODEL: Homology modelling of protein structures and complexes. Nucleic Acids Res. 46:W296–W303. 10.1093/nar/gky427 29788355 PMC6030848

[bib98] Wick, K.R., and M.T. Berton. 2000. IL-4 induces serine phosphorylation of the STAT6 transactivation domain in B lymphocytes. Mol. Immunol. 37:641–652. 10.1016/S0161-5890(00)00088-2 11164892

[bib99] Yildiz, M., H. Li, D. Bernard, N.A. Amin, P. Ouillette, S. Jones, K. Saiya-Cork, B. Parkin, K. Jacobi, K. Shedden, . 2015. Activating STAT6 mutations in follicular lymphoma. Blood. 125:668–679. 10.1182/blood-2014-06-582650 25428220 PMC4729538

[bib100] Zamò, A., J. Pischimarov, H. Horn, G. Ott, A. Rosenwald, and E. Leich. 2018. The exomic landscape of t(14;18)-negative diffuse follicular lymphoma with 1p36 deletion. Br. J. Haematol. 180:391–394. 10.1111/bjh.15041 29193015

[bib101] Zhang, Q., B. Boisson, V. Béziat, A. Puel, and J.L. Casanova. 2018. Human hyper-IgE syndrome: Singular or plural? Mamm. Genome. 29:603–617. 10.1007/s00335-018-9767-2 30094507 PMC6317873

[bib102] Zhu, Z., Y. Guo, H. Shi, C.-L. Liu, R.A. Panganiban, W. Chung, L.J. O’Connor, B.E. Himes, S. Gazal, K. Hasegawa, . 2020. Shared genetic and experimental links between obesity-related traits and asthma subtypes in UK Biobank. J. Allergy Clin. Immunol. 145:537–549. 10.1016/j.jaci.2019.09.035 31669095 PMC7010560

[bib103] Zhu, Z., P.H. Lee, M.D. Chaffin, W. Chung, P.-R. Loh, Q. Lu, D.C. Christiani, and L. Liang. 2018. A genome-wide cross-trait analysis from UK Biobank highlights the shared genetic architecture of asthma and allergic diseases. Nat. Genet. 50:857–864. 10.1038/s41588-018-0121-0 29785011 PMC5980765

[bib104] Zhu, Z., X. Zhu, C.-L. Liu, H. Shi, S. Shen, Y. Yang, K. Hasegawa, C.A. Camargo Jr, and L. Liang. 2019. Shared genetics of asthma and mental health disorders: A large-scale genome-wide cross-trait analysis. Eur. Respir. J. 54:1901507. 10.1183/13993003.01507-2019 31619474

