# Supplementary material for: Human germline heterozygous gain-of-function STAT6 variants cause severe allergic disease
Source: J Exp Med. 2023 Mar 8;220(5):e20221755. doi: 10.1084/jem.20221755 (PMC10037107; doi:10.1084/jem.20221755)
Supplement: Table S1 — lists primers used for site-directed mutagenesis. [file JEM_20221755_TableS1.docx]

**Table S1**. List of primers used for site-directed mutagenesis

| Type | Variant | Forward primer (5′-3′) | Reverse primer (5′-3′) |
| --- | --- | --- | --- |
| P1/P6 | c.1256A>G; p.D419G | GGCAACCAAGGCAACAATGCC | ATGGACGATGACCACCAG |
| P2 | c.1256A>C;  p.D419A | GGCAACCAAGCCAACAATGCC | ATGGACGATGACCACCAG |
| P3/P4 | c.1255G>T  ; p.D419Y | TGGCAACCAATACAACAATGCC | TGGACGATGACCACCAGG |
| P5 | c.1255G>A; p.D419N | TGGCAACCAAAACAACAATGC | TGGACGATGACCACCAGG |
| P7/P8/P9 | c.1255G>C; p.D419H | TGGCAACCAACACAACAATGCC | TGGACGATGACCACCAGG |
| P10 | c.1144G>C; p.E382Q | TGTCACAGAGCAGAAGTGCGC | GACTCAGTGCCCTTCCGC |
| P11 | c.1784A>G;  p.K595R | TTCTCTGCCAGAGACCTGTCC | TGGCTGGATGTTCTCTATC |
| P12 | c.1928C>G; p.P643R | GGTTATGTCCGAGCTACCATC | CCTGCCATCCTTACCCAT |
| P13/P14/P15/P16 | c.1555C>G; p.D519H | GCAGTGGTTTCATGGTGTCCT | CAAAAGGTGAAGCCACGG |
| Population variant | c.962C>T;  p.A321V | GAGAAGCAGGTGCGGGAGCTG | TGTCACCATGTCGGCCCTG |
| Negative control | c.1922A>T;  p.Y641F | GGCAGGGGTTTTGTCCCAGCT | ATCCTTACCCATCTGTTCAGG |
