# Supplementary material for: Human germline heterozygous gain-of-function STAT6 variants cause severe allergic disease
Source: J Exp Med. 2023 Mar 8;220(5):e20221755. doi: 10.1084/jem.20221755 (PMC10037107; doi:10.1084/jem.20221755)
Supplement: Table S2 — lists antibodies used for phospho-flow on different immune subsets. [file JEM_20221755_TableS2.docx]

**Table S2**. List of antibodies used for phospho-flow on different immune subsets

| *Marker* | *Fluorophore* | *Clone* | *Catalog #:* |
| --- | --- | --- | --- |
| Anti-CD56 | PE-cy7 | N901 | A21692 (Beckman Coulter) |
| Anti-CD20 | BV421 | 2H7 | 302302 (BioLegend) |
| Anti-CD14 | FITC | RMO52 | B36297 (Beckman Coulter) |
| Anti-CD3 | APC | UCHT1 | 555335 (BD) |
| Anti-pSTAT6 (p.Y641) | PE | 18/P-Stat6 | 562078 (BD) |
