# Supplementary material for: Human germline heterozygous gain-of-function STAT6 variants cause severe allergic disease
Source: J Exp Med. 2023 Mar 8;220(5):e20221755. doi: 10.1084/jem.20221755 (PMC10037107; doi:10.1084/jem.20221755)
Supplement: Table S3 — lists antibodies used for TH phenotyping in patient PBMCs. [file JEM_20221755_TableS3.docx]

**Table S3.** List of antibodies used for T_H_ phenotyping in patient PBMCs

| *Marker* | *Fluorophore* | *Clone* | *Catalog #:* |
| --- | --- | --- | --- |
| Cell surface markers | | | |
| Anti-CD3 | BV510 | UCHT1 | 563109 (BD) |
| Anti-CD8 | BV570 | HIT8a | 563550 (BD) |
| Anti-CD4 | BUV563 | SK3 | 563550 (BD) |
| Anti-CD27 | BV605 | L128 | 562655 (BD) |
| Anti-CD45RA | BV421 | HI100 | 562885 (BD) |
| Anti-CD25 | BUV805 | M-A251 | 742011 (BD) |
| Intracellular markers | | | |
| Anti-FoxP3 | AF488 | 259D/C7 | 560047 (BD) |
| Anti-TNF | R718 | Mab11 | 566957 (BD) |
| Anti-IL-5 | PE | TRFK5 | 504303 (BioLegend) |
| Anti-IL-13 | Pe-Cy7 | JES10-5A2 | 501913 (BioLegend) |
| Anti-IFNγ | BV711 | 4S.B3 | 564793 (BD) |
| Anti-IL-4 | PeCF594 | MP4-25D2 | 565161 (BD) |
| Anti-IL-17A | PerCP-Cy5.5 | N49-653 | 560799 (BD) |
| Anti-IL-21 | AF647 | 3A3-N2.1 | 560493 (BD) |
| Fixable viability stain | AF780 |  | 565388 (BD) |
