# Supplementary material for: Human germline heterozygous gain-of-function STAT6 variants cause severe allergic disease
Source: J Exp Med. 2023 Mar 8;220(5):e20221755. doi: 10.1084/jem.20221755 (PMC10037107; doi:10.1084/jem.20221755)
Supplement: Table S4 — shows variant annotation and pathogenicity prediction of the variants reported within the DNA-binding domain of STAT6 for 10 patients. [file JEM_20221755_TableS4.docx]

**Table S4.** Variant annotation and pathogenicity prediction of the variants reported within the DNA-binding domain of STAT6 for 10 patients

|  | **P1/P6** | **P2** | **P3/P4** | **P5** | **P7/P8/P9** | **P10** |
| --- | --- | --- | --- | --- | --- | --- |
| Chromosome | 12 | 12 | 12 | 12 | 12 | 12 |
| Genomic position (GRCh37) | 57496661 | 57496661 | 57496662 | 57496662 | 57496662 | 57498315 |
| cDNA position (NM_001178079.2) | 1256 | 1256 | 1255 | 1255 | 1255 | 1144 |
| Nucleotide reference | A | A | G | G | G | G |
| Nucleotide variant | G | C | T | A | C | C |
| Protein variant (NP_001171550.) | p.Asp419Gly (p.D419G) | p.Asp419Ala (p.D419A) | p.Asp419Tyr (p.D419Y) | p.Asp419Asn (p.D419N) | p.Asp419His (p.D419H) | p.Glu382Gln (p.E382Q) |
| WT amino acid → variant amino acid | Negatively charged **→**  Non-polar | Negatively charged **→**  Non-polar | Negatively charged **→**  Non-polar | Negatively charged **→**  Polar | Negatively charged **→** Positively charged | Negatively charged **→**  Polar |
| Protein domain | DBD | DBD | DBD | DBD | DBD | DBD |
| Zygosity | Heterozygous | Heterozygous | Heterozygous | Heterozygous | Heterozygous | Heterozygous |
| Inheritance | de novo | de novo | Maternally inherited for P4 | de novo | Maternally inherited for P8/P9 | de novo |
| dbSNP153 | No entry | No entry | No entry | rs11172102 | No entry | No entry |
| gnomAD (v3.1.1) | No entry | No entry | No entry | No entry | No entry | No entry |
| COSMIC (v95) | Somatic reported, 26 entries (COSV55668829) | Somatic reported, 3 entries (COSV55672176) | Somatic reported, 2 entries (COSV55670334) | Somatic reported, 9 entries (COSV55668904) | Somatic reported, 9 entries (COSV55672315) | Somatic not reported |
| In silico pathogenicity prediction models | | | | | | |
| CADD (v1.6) | 29.3 | 29.3 | 34.0 | 34.0 | 33.0 | 27.7 |
| SIFT | Deleterious (0.001) | Damaging (0.004) | Damaging (0.000) | Damaging (0.001) | Damaging (0.001) | Deleterious (0.026) |
| PolyPhen-2 | Probably damaging (1) | Probably damaging (1) | Probably damaging (1) | Probably damaging (1) | Probably damaging (1) | Probably damaging (1) |
| LRT | Deleterious (0) | Deleterious (0) | Deleterious (0) | Deleterious (0) | Deleterious (0) | Deleterious (0) |
| MutationTaster | Disease causing (1) | Disease causing (1) | Disease causing (1) | Disease causing (1) | Disease causing (1) | Disease causing (1) |
| PROVEAN | Damaging (−4.83) | Damaging (−4.74) | Damaging (−5.64) | Damaging (−3.02) | Damaging (−3.83) | Damaging (−2.71) |
| MetaSVM | Damaging (0.610) | Tolerable (−0.522) | Tolerable (−0.503) | Tolerable (−0.503) | Tolerable (−0.503) | Tolerable (−0.538) |
| M-CAP | Possibly pathogenic (0.294) | Damaging (0.172) | Damaging (0.156) | Damaging (0.129) | Damaging (0.149) | Possibly pathogenic (0.106) |
| FATHMM MKL coding | Deleterious (0.701) | Damaging (0.900) | Damaging (0.962) | Damaging (0.959) | Damaging (0.963) | Deleterious (0.986) |
