# Supplementary material for: Human germline heterozygous gain-of-function STAT6 variants cause severe allergic disease
Source: J Exp Med. 2023 Mar 8;220(5):e20221755. doi: 10.1084/jem.20221755 (PMC10037107; doi:10.1084/jem.20221755)
Supplement: Table S5 — shows variant annotation and pathogenicity prediction of the variants reported outside of the DNA-binding domain of STAT6 for six patients. [file JEM_20221755_TableS5.docx]

**Table S5.** Variant annotation and pathogenicity prediction of the variants reported outside of the DNA-binding domain of STAT6 for six patients

|  | **P11** | **P12** | **P13/P14/P15/P16** |
| --- | --- | --- | --- |
| Chromosome | 12 | 12 | 12 |
| Genomic Position (GRCh37) | 57493184 | 57492825 | 57493831 |
| cDNA position (NM_001178079.2) | 1784 | 1928 | 1555 |
| Nucleotide reference | A | C | G |
| Nucleotide variant | G | G | C |
| Protein variant (NP_001171550.) | p.Lys595Arg  (p.K595R) | p.Pro643Arg  (p.P643R) | p.Asp519His  (p.D519H) |
| WT amino acid → variant amino acid | Positively charged **→**  Positively charged | Polar **→** Positively charged | Negatively charged **→** Positively charged |
| Protein domain | SH2 | TAD | LD |
| Zygosity | Heterozygous | Heterozygous | Heterozygous |
| Inheritance | de novo | de novo | *??* |
| dbSNP153 | No entry | No entry | No entry |
| gnomAD (v3.1.1) | No entry | No entry | No entry |
| COSMIC (v95) | Somatic not reported | Somatic reported, 4 entries (COSV105175958) | Somatic reported, 9 entries (COSV55666936) |
| In silico pathogenicity prediction models | | | |
| CADD (v1.6) | 23.2 | 24.3 | 24.9 |
| SIFT | Tolerable (0.52) | Tolerable (0.132) | Tolerable (0.213) |
| PolyPhen-2 (HDIV) | Possibly damaging (0.905) | Possibly damaging (0.954) | Probably damaging (1) |
| LRT | Deleterious (0) | Deleterious (0) | Deleterious (0) |
| MutationTaster | Disease causing (1) | Disease causing (1) | Disease causing (1) |
| PROVEAN | Tolerable (−0.76) | Tolerable (−1.67) | Tolerable (−0.42) |
| MetaSVM | Damaging (0.449) | Damaging (0.697) | Tolerable (−0.624) |
| M-CAP | Damaging (0.056) | Damaging (0.095) | Damaging (0.032) |
| FATHMM MKL coding | Damaging (0.847) | Damaging (0.933) | Damaging (0.61) |
