# Supplementary material for: Human germline heterozygous gain-of-function STAT6 variants cause severe allergic disease
Source: J Exp Med. 2023 Mar 8;220(5):e20221755. doi: 10.1084/jem.20221755 (PMC10037107; doi:10.1084/jem.20221755)
Supplement: Table S6 — lists genes upregulated (gray) and downregulated (red) in transduced WT-, p.E382Q-, and p.D419G-transduced Jurkats that meet the cutoff of fold-change (FC) > 1.25 and adjusted P value <0.05. [file JEM_20221755_TableS6.docx]

**Table S6.** List of genes upregulated (gray) and downregulated (red) in transduced WT-, p.E382Q-, and p.D419G-transduced Jurkats that meet the cutoff of fold-change (FC) > 1.25 and adjusted P value < 0.05

| **IL4-WT-STAT6 targets in Jurkats** | | | | **IL4-p.E382Q-STAT6 targets in Jurkats** | | | | **IL4-p.D419G-STAT6 targets in Jurkats** | | | |
| --- | --- | --- | --- | --- | --- | --- | --- | --- | --- | --- | --- |
| *SOCS1* | *STK17B* | *IRF2BP2* | *IDI1* | *SOCS1* | *ANKRD12* | *CNPY3* | *DPYSL2* | *SOCS1* | *HHIP* | *EIF3A* | *BTN3A1* |
| *MAL* | *SLC39A8* | *UBE2B* | *CHST2* | *CISH* | *PI4K2B* | *TBC1D16* | *CD79A* | *CISH* | *PTGER4* | *CWF19L1* | *KIAA1551* |
| *FAM171A1* | *MZT1* | *TEX30* | *RPF2* | *SNTB1* | *PRKCQ-AS1* | *RANBP10* | *ADAMTS17* | *SNTB1* | *PAFAH2* | *ETS1* | *NAA16* |
| *CD244* | *MBNL1* | *ABCD3* | *LYPLA1* | *FAM171A1* | *SLC39A8* | *TGIF2* | *TNFRSF1A* | *MAL* | *ABCD3* | *ZNF197* | *E2F2* |
| *BCL6* | *ETS2* | *SCOC* | *CCNC* | *TRIB1* | *TEX30* | *SRC* | *RXRA* | *CD244* | *SMARCA2* | *SRSF8* | *TGIF2* |
| *IL4R* | *PMAIP1* | *NDUFA5* | *CENPH* | *EVI2B* | *TTC9C* | *RHOU* | *ARHGEF10* | *FAM171A1* | *CDC42BPA* | *ATRX* | *CNN2* |
| *CISH* | *ENDOD1* | *SNX10* | *CBX3* | *CD244* | *DOCK10* | *AKT2* | *PCBP3* | *BCL6* | *ST8SIA4* | *PSMA6* | *PVRL1* |
| *RAB11FIP1* | *TMED7* | *LZTFL1* | *HINT3* | *EVI2A* | *ABCD3* | *SSBP3* | *PRKD2* | *EVI2B* | *UGP2* | *PTDSS1* | *NLRC3* |
| *TREML2* | *ST8SIA4* | *COX20* | *XIAP* | *XBP1* | *CASP3* | *ICAM2* | *SLA2* | *GAB2* | *FAM120AOS* | *IPP* | *RGS14* |
| *SNTB1* | *PRKCQ-AS1* | *GRK6* | *CD164* | *RORA* | *EEF2K* | *GLUL* | *TMEM173* | *XBP1* | *SPATA5* | *CLPB* | *GRAMD4* |
| *PECAM1* | *TSNAX* | *LINC00493* | *STK17A* | *ARHGAP25* | *CYLD* | *ARHGAP1* | *FGFR1* | *TTC39B* | *ETS2* | *IRF2BP2* | *LIME1* |
| *GCSAM* | *NDUFB5* | *DCK* | *DSTN* | *MAL* | *CHD1* | *RPS6KA1* | *SLC7A5* | *IL4R* | *CTDP1* | *MRPS28* | *SMIM3* |
| *RASGRP1* | *XBP1* | *STAMBPL1* | *ARPC5* | *GCNT4* | *PLCB1* | *FMNL1* | *NOTCH1* | *RAB11FIP1* | *XPNPEP3* | *GNL3L* | *AEBP1* |
| *GATSL3* | *RFK* | *SGCB* | *RAP1GAP* | *BCL6* | *OIP5-AS1* | *LOC100507600* | *FAM78A* | *PECAM1* | *SMCO4* | *SLC25A15* | *ATP9A* |
| *FAM120AOS* | *TESPA1* | *DENR* | *CLSTN3* | *NT5E* | *PDP2* | *GIMAP6* | *DUSP2* | *ZNF799* | *PLEKHA2* | *HNRNPA2B1* | *SNHG1* |
|  |  |  |  | *STK17B* | *TESPA1* | *PTPN7* | *SLC6A6* | *ENDOD1* | *LRRC28* | *STK17A* | *NKD2* |
|  |  |  |  | *LOC101927482* | *ZNF302* | *CXCR4* | *GAS7* | *EPAS1* | *TNFAIP8* | *TTC33* | *HES4* |
|  |  |  |  | *PECAM1* | *SOCS7* | *LAIR1* | *KLF13* | *ARHGAP25* | *CEP128* | *PRKCQ-AS1* | *NSUN5P2* |
|  |  |  |  | *TTC39B* | *CENPE* | *LRP5* | *PPP1R16B* | *TREML2* | *BCAT1* | *VMP1* | *SEMA7A* |
|  |  |  |  | *IL4R* | *ST3GAL5* | *SLIT1* | *CD5* | *STK17B* | *LRMP* | *RPL9* | *DTX1* |
|  |  |  |  | *GCSAM* | *RDX* | *BCL7A* | *ARHGAP23* | *GCNT4* | *SLC37A3* | *TSHR* | *RASGRP2* |
|  |  |  |  | *RNF125* | *PDCD4* | *LDOC1L* | *SBK1* | *GATSL3* | *CADM1* | *GLRX* | *SIT1* |
|  |  |  |  | *BATF* | *BRCA2* | *ADCY7* | *TMC8* | *PRKCE* | *DNAJC9* | *GLE1* | *CD82* |
|  |  |  |  | *ENDOD1* | *BIVM* | *ARHGEF2* | *SLC29A4* | *PTCHD2* | *CEP97* | *ADAM10* | *PIK3C2B* |
|  |  |  |  | *PMAIP1* | *ELOVL4* | *DHCR7* | *ELF4* | *PEX5L* | *TWSG1* | *MGAT1* | *NOTCH3* |
|  |  |  |  | *RGPD3* | *DSTN* | *SSH2* | *TET3* | *GAB3* | *FAM20B* | *AP2A2* | *CHPF2* |
|  |  |  |  | *STAMBPL1* | *GNPDA1* | *BCL9* | *PMEPA1* | *PMAIP1* | *RB1* | *SLC6A6* | *FAM53B* |
|  |  |  |  | *CD69* | *ITGA4* | *ATG4D* | *CHRNA3* | *S1PR1* | *ITPR2* | *SSBP3* | *FAM73B* |
|  |  |  |  | *SLC4A4* | *VAV3* | *ATP8B2* | *FXYD2* | *RORA* | *IGF2R* | *ZNF33B* | *TRIM24* |
|  |  |  |  | *TNFRSF14* | *RCBTB2* | *C19orf47* | *BLK* | *FLT3LG* | *PLEKHB2* | *CD28* | *DOK2* |
|  |  |  |  | *TMX3* | *DNAJC9* | *CDR2L* | *ERGIC1* | *ZNF443* | *EPB41L5* | *RNF44* | *CHRNA3* |
|  |  |  |  | *HHIP-AS1* | *PGRMC2* | *LCK* | *PLXND1* | *ARSB* | *ZNF101* | *ITGA6* | *C1orf159* |
|  |  |  |  | *LINC01215* | *ITGAV* | *DNAJC5* | *KCNH2* | *ADGRG1* | *ERLIN2* | *MAT2A* | *ARHGAP9* |
|  |  |  |  | *PPM1K* | *FAM120AOS* | *ELK1* | *CNN2* | *RASGRP1* | *CHD1* | *AGAP2* | *CDKN2D* |
|  |  |  |  | *RASGRP1* | *CD2* | *CTDSP2* | *GIMAP4* | *KRT1* | *DOCK10* | *TAZ* | *SIX6* |
|  |  |  |  | *RAB11FIP1* | *NFE2L3* | *ACSF3* | *ITGAL* | *STAMBPL1* | *CEP85L* | *SPG7* | *RAP1GAP* |
|  |  |  |  | *S1PR1* | *RPS10* | *PSD4* | *PIK3C2B* | *AUTS2* | *GRAP2* | *RANBP6* | *TPST2* |
|  |  |  |  | *GAB3* | *RNF11* | *SCAMP4* | *KIAA0125* | *RNF125* | *CASP3* | *KIF21B* | *PTCH1* |
|  |  |  |  | *ENTPD4* | *IKBIP* | *LASP1* | *TPST2* | *GCSAM* | *MYB* | *NOTCH1* | *LENG8* |
|  |  |  |  | *TWSG1* | *EXTL2* | *ARF3* | *USP20* | *AFF1* | *KCTD17* | *POLM* | *CLK1* |
|  |  |  |  | *EPAS1* | *IFT80* | *CCNI* | *CD6* | *GRK5* | *ALDH1A2* | *KCNH2* | *HDAC7* |
|  |  |  |  | *LINS* | *SPINT2* | *ITGA5* | *SPSB1* | *SLC4A4* | *TGFBR1* | *FAM102A* | *TBC1D10C* |
|  |  |  |  | *B3GALNT2* | *BNIP2* | *CD7* | *CECR1* | *RRBP1* | *OIP5-AS1* | *LRRC8A* | *BZRAP1-AS1* |
|  |  |  |  | *PEX5L* | *HIVEP3* | *SLC35D1* | *SMIM3* | *LINC01215* | *CPPED1* | *TRABD* | *SH3TC1* |
|  |  |  |  | *SCAI* | *MFN1* | *AGO1* | *E2F2* | *PPM1K* | *CNOT6L* | *ACSF3* | *OSBPL7* |
|  |  |  |  | *ARAP2* | *DBT* | *ATP2A3* | *BCL9L* | *SPINT2* | *ARNTL2* | *CHTF18* | *SLC5A3* |
|  |  |  |  | *PPAP2A* | *CTDP1* | *PTPN6* | *MOB3A* | *SOCS7* | *SCD5* | *LEF1* | *PMEPA1* |
|  |  |  |  | *GATSL3* | *TFAP2C* | *RCSD1* | *PI16* | *LZTS1* | *TMEM87B* | *SWI5* | *BAHCC1* |
|  |  |  |  | *CEP85L* | *GOLGA4* | *KIAA1549* | *IL9R* | *TESPA1* | *MPPED2* | *RASAL3* | *USP20* |
|  |  |  |  | *SCN3A* | *IDI1* | *ARHGAP9* | *SIT1* | *APOL6* | *IGFBP2* | *SASH3* | *TMC8* |
|  |  |  |  | *SLC12A2* | *CBWD2* | *VAV1* | *CDKN2D* | *CYP20A1* | *IKZF1* | *RCSD1* | *LAT* |
|  |  |  |  | *TREML2* | *SDCBP* | *EMP3* | *ZKSCAN8* | *BAG2* | *BMPR1A* | *MYO1G* | *CBFA2T3* |
|  |  |  |  | *CHST2* | *LZTFL1* | *SWI5* | *LAT* | *CHST2* | *TMX3* | *RALGDS* | *DDIT4* |
|  |  |  |  | *PLCL1* | *MYB* | *MOCS1* | *NOTCH3* | *RRAS2* | *CD2* | *HMHA1* | *SOX11* |
|  |  |  |  | *TBC1D4* | *PTPRC* | *RPL29* | *ATP9A* | *B3GALNT2* | *KCNK5* | *ANKRD13B* | *TUBGCP6* |
|  |  |  |  | *AFF1* | *TNFRSF21* | *HDAC5* | *FAM53B* | *FAM63B* | *TAF15* | *ITGAL* | *RHOU* |
|  |  |  |  | *SMARCA2* | *SNRPF* | *PLAGL2* | *SASH3* | *GNPDA1* | *ABCB10* | *DGKZ* | *PAXIP1-AS1* |
|  |  |  |  | *BIRC3* | *PRKCQ* | *HDAC7* | *PTCH1* | *TTC9C* | *FAM101B* | *LOC100507600* | *PI16* |
|  |  |  |  | *FLT3LG* | *CEP78* | *LIMD2* | *DTX1* | *RPS3A* | *TBC1D4* | *MOB3A* | *VAMP1* |
|  |  |  |  | *HHIP* | *ADAM17* | *TMEM63A* | *CD82* | *HIVEP3* | *FAM120A* | *S1PR3* | *RAP1GAP2* |
|  |  |  |  | *RRBP1* | *SYPL1* | *PTCRA* | *STAT5A* | *SLC39A10* | *FAM46C* | *CD6* | *SNHG3* |
|  |  |  |  | *UGP2* | *HNRNPD* | *ACTN1* | *FCMR* | *MBNL1* | *SEC11C* | *PTPN6* | *STAT5A* |
|  |  |  |  | *LRMP* | *RPIA* | *CORO1A* | *RAP1GAP* | *SLC39A8* | *RAB21* | *TMEM173* | *IQGAP3* |
|  |  |  |  | *GRK5* | *ZNF101* | *PTPN3* | *DDIT4* | *PRKCQ* | *ASB7* | *SLA* | *RORC* |
|  |  |  |  | *TGFBR1* | *VHL* | *AKNA* | *SH3TC1* | *SCN3A* | *JUND* | *SH3BP5* | *ID3* |
|  |  |  |  | *STK17A* | *GGH* | *LAMP3* | *CBFA2T3* | *EEF2K* | *ARID5B* | *TCF7* | *ADCYAP1* |
|  |  |  |  | *AUTS2* | *SNHG8* | *RAG1* | *HES1* | *P2RY8* | *WASF2* | *TMEM69* | *HES1* |
|  |  |  |  | *EFR3A* | *SRPK2* | *AGAP2* | *AFF3* |  |  |  |  |
|  |  |  |  | *PCDH9* | *LRRC8A* | *SEMA7A* | *RAP1GAP2* | |  |  |  |
|  |  |  |  | *MPPED2* | *PRMT1* | *MYO1G* | *PLEKHF1* |  |  |  |  |
|  |  |  |  | *BAG2* | *SELPLG* | *ABLIM1* | *SLC43A2* |  |  |  |  |
|  |  |  |  | *TNFAIP8* | *NXN* | *SLC35F6* | *PLCH1* |  |  |  |  |
|  |  |  |  | *PAFAH2* | *TUBB* | *FAM102A* | *PVRL1* |  |  |  |  |
|  |  |  |  | *RCN1* | *ZDHHC5* | *KIF21B* | *ID3* |  |  |  |  |
|  |  |  |  | *NCOA7* | *CYB561A3* | *NLRC3* | *GPR68* |  |  |  |  |
|  |  |  |  | *CSGALNACT2* | *SLA* | *TNFSF4* | *RORC* |  |  |  |  |
|  |  |  |  | *CMAHP* | *APH1A* | *MKNK2* |  |  |  |  |  |
