# Supplementary material for: Human germline heterozygous gain-of-function STAT6 variants cause severe allergic disease
Source: J Exp Med. 2023 Mar 8;220(5):e20221755. doi: 10.1084/jem.20221755 (PMC10037107; doi:10.1084/jem.20221755)
Supplement: Table S7 — lists genes in the leading edge driving the enrichment of two pathways (IL-4/STAT6 pathway and TH2 pathway) between transduced WT- vs. p.E382Q- and WT- vs. p.D419G- Jurkats [file JEM_20221755_TableS7.docx]

**Table S7.** List of genes in the leading edge driving the enrichment of two pathways (IL-4/STAT6 pathway and TH2 pathway) between transduced WT- vs. p.E382Q- and WT- vs. p.D419G- Jurkats.

| **IL-4/STAT6 targets in unstim** | | **TH2 targets in IL-4 stim** | |
| --- | --- | --- | --- |
| **E382Q vs. WT** | **D419G vs. WT** | **E382Q vs. WT** | **D419G vs. WT** |
| *GCSAM* | *CD244* | *TRIB1* | *CISH* |
| *SNTB1* | *NDUFB5* | *SLCO3A1* | *OSBPL1A* |
| *BCL6* | *RPF2* | *ARHGAP25* | *HCLS1* |
| *IL4R* | *CENPH* | *XBP1* | *EVI2B* |
| *STK17B* | *TSNAX* | *SLC18A2* | *XBP1* |
| *XBP1* | *CBX3* | *ANXA1* | *ARHGAP25* |
| *ST8SIA4* | *DENR* | *EVI2B* | *ZNF443* |
| *PRKCQ-AS1* | *TMED7* | *FLT3LG* | *CD244* |
| *NDUFB5* | *RFK* | *SNTB1* | *NKG7* |
| *NDUFA5* | *MZT1* | *OSBPL1A* | *SNTB1* |
| *CENPH* | *SCOC* | *BATF* | *CDK6* |
| *LINC00493* | *NDUFA5* | *TGFBR2* | *EPAS1* |
| *COX20* | *TEX30* | *STK17B* | *FLT3LG* |
| *ETS2* | *LZTFL1* | *RNF125* | *HPGD* |
| *MBNL1* | *LYPLA1* | *EPAS1* | *KRT1* |
| *DCK* | *COX20* | *LIMS2* | *ETS1* |
| *FAM171A1* | *UBE2B* | *CISH* | *SPINT2* |
|  | *PRKCQ-AS1* | *CDK6* | *RNF125* |
|  | *CCNC* | *PLCL1* | *AUTS2* |
|  | *XIAP* | *DAPK1* | *ZC3HAV1L* |
|  | *IDI1* | *PRKXP1* | *KCNK5* |
|  | *LINC00493* | *NKG7* | *AFF1* |
|  | *SGCB* | *AFF1* | *STK17B* |
|  | *IL4R* | *FAM69A* | *NFU1* |
|  | *HINT3* | *PDE9A* | *IKZF2* |
|  | *DCK* | *TTC9C* | *CLCN3* |
|  | *MBNL1* | *AUTS2* | *ATP6V0A2* |
|  | *SNX10* | *ATP6V0A2* | *APOL6* |
|  |  | *CHN2* | *PLEKHB2* |
|  |  | *ZC3HAV1L* | *TGFBR2* |
|  |  | *ETS1* | *CYLD* |
|  |  | *BCL2L11* | *TTC9C* |
|  |  | *CD1C* | *ARG2* |
|  |  | *SGSH* | *HIPK2* |
|  |  | *ZNF443* | *ETNK1* |
|  |  | *HEG1* | *SKAP1* |
|  |  | *STAMBPL1* | *MAPKAPK3* |
|  |  | *CD46* | *PPM1K* |
|  |  | *NFU1* | *MTDH* |
|  |  | *HIPK2* | *DPH5* |
|  |  | *HCLS1* | *AUH* |
|  |  | *ANKRD44* | *PTDSS1* |
|  |  | *FLJ37453* | *CASP3* |
|  |  | *TRAK2* | *STAMBPL1* |
|  |  | *C4orf32* | *ANKRD44* |
|  |  | *SKAP1* | *SEC11C* |
|  |  | *DZIP3* | *CD1C* |
|  |  | *KIAA0922* | *TFDP1* |
|  |  | *DDB2* | *FAM69A* |
|  |  | *IFT80* | *HEG1* |
|  |  | *GOLGA8B* | *LNPEP* |
|  |  | *MTBP* | *TROVE2* |
|  |  | *KCNK6* | *ZYG11B* |
|  |  | *CYLD* | *GFPT1* |
|  |  | *FAM46C* | *PPP3CA* |
|  |  | *ATP11A* | *MRPL35* |
|  |  | *EVL* | *PPP4R2* |
|  |  | *DYRK2* | *IL4R* |
|  |  | *LNPEP* | *PITPNB* |
|  |  | *AKAP13* | *DARS* |
|  |  | *RNF146* | *GLS* |
|  |  | *GFI1* | *VIM* |
|  |  | *SH3KBP1* | *C4orf32* |
|  |  | *PRKX* | *CCDC117* |
|  |  | *PSMB9* | *XRCC5* |
|  |  | *LCP2* | *STK39* |
|  |  |  | *CTNNAL1* |
|  |  |  | *F11R* |
|  |  |  | *FOXP1* |
|  |  |  | *RAB30* |
|  |  |  | *EEF2K* |
|  |  |  | *AKAP13* |
|  |  |  | *CD55* |
|  |  |  | *LEPROTL1* |
|  |  |  | *UFD1L* |
|  |  |  | *BCL2L11* |
|  |  |  | *KPNA4* |
|  |  |  | *IMMT* |
|  |  |  | *SOCS1* |
|  |  |  | *NSMCE1* |
|  |  |  | *HSPH1* |
|  |  |  | *ATP11A* |
|  |  |  | *PRKXP1* |
|  |  |  | *DR1* |
|  |  |  | *RNF146* |
|  |  |  | *SLC17A5* |
|  |  |  | *MAT2B* |
|  |  |  | *DGKD* |
|  |  |  | *PARVG* |
|  |  |  | *PSMB9* |
|  |  |  | *SYNE1* |
