# Supplementary material for: Human germline heterozygous gain-of-function STAT6 variants cause severe allergic disease
Source: J Exp Med. 2023 Mar 8;220(5):e20221755. doi: 10.1084/jem.20221755 (PMC10037107; doi:10.1084/jem.20221755)
Supplement: Data S1 — provides clinical narratives for each patient. [file JEM_20221755_DataS1.pdf]

CLINICAL NARRATIVES

Patient 1

Patient 1 (P1) is a female who had short stature (**Fig 7**) and life-long struggles with severe allergic disease. The patient was born to non-consanguineous parents of Middle Eastern descent and she has three younger healthy brothers. Multiple family members report food sensitivity and her father died of lung cancer at age 55 years. She presented with severe chronic, treatment-resistant atopic dermatitis since birth. She was diagnosed with IgE mediated food allergy to egg, milk, peanut and tree nuts during the first 2 years of her life. She was diagnosed with asthma as an infant. At age 1 year she had a pneumothorax requiring chest tube insertion, and at age 2 years required intubation for an asthma exacerbation. At age 4 years she was diagnosed with giant papillary conjunctivitis which has remained an ongoing challenge. Other eye manifestations included unilateral retinal detachment at age 16 years and the development of cataracts requiring surgery at age 23 years. Through her second and third decades of life she continued to suffer from eczema, asthma, recurrent bronchitis, rhino conjunctivitis, and secondary staphylococcal and candida skin infections. She had multiple dental abscess in the second and third decades of her life. In her 20s she began experiencing gastrointestinal symptoms culminating in biopsy diagnosed eosinophilic esophagitis and eosinophilic gastroenteritis. Serial blood testing over the years confirmed eosinophilia and high serum IgE levels. Brain MRI at age 35 years revealed multiple anatomical variants including anomalous vasculature in the circle of Willis with hypoplastic vertebrobasilar arteries, a persistent left-sided congenital trigeminal artery with tortuosity, and a hypoplastic A1 segment of the right anterior carotid artery. Multiple therapies were trialed over the years with corticosteroids offering the most obvious benefits. At age 34 years she started dupilumab and after 3 months she began to experience benefit. With continued treatment, her skin improved dramatically (Eczema Area and Severity Index (EASI) score fell from 65 (very severe) to 3.5 (mild)), she was able to wean off oral corticosteroids (required 20mg/day prednisone before dupilumab), and she was able to discontinue daily antihistamine which was used for pruritus. To seek a diagnosis for this spectrum of severe allergic manifestations, whole exome sequencing (WES) was performed on the patient, her mother and one of her brothers and a de novo heterozygous variant for *STAT6* (NM_001178079.2) at c.1256A>G, p.D419G was identified.

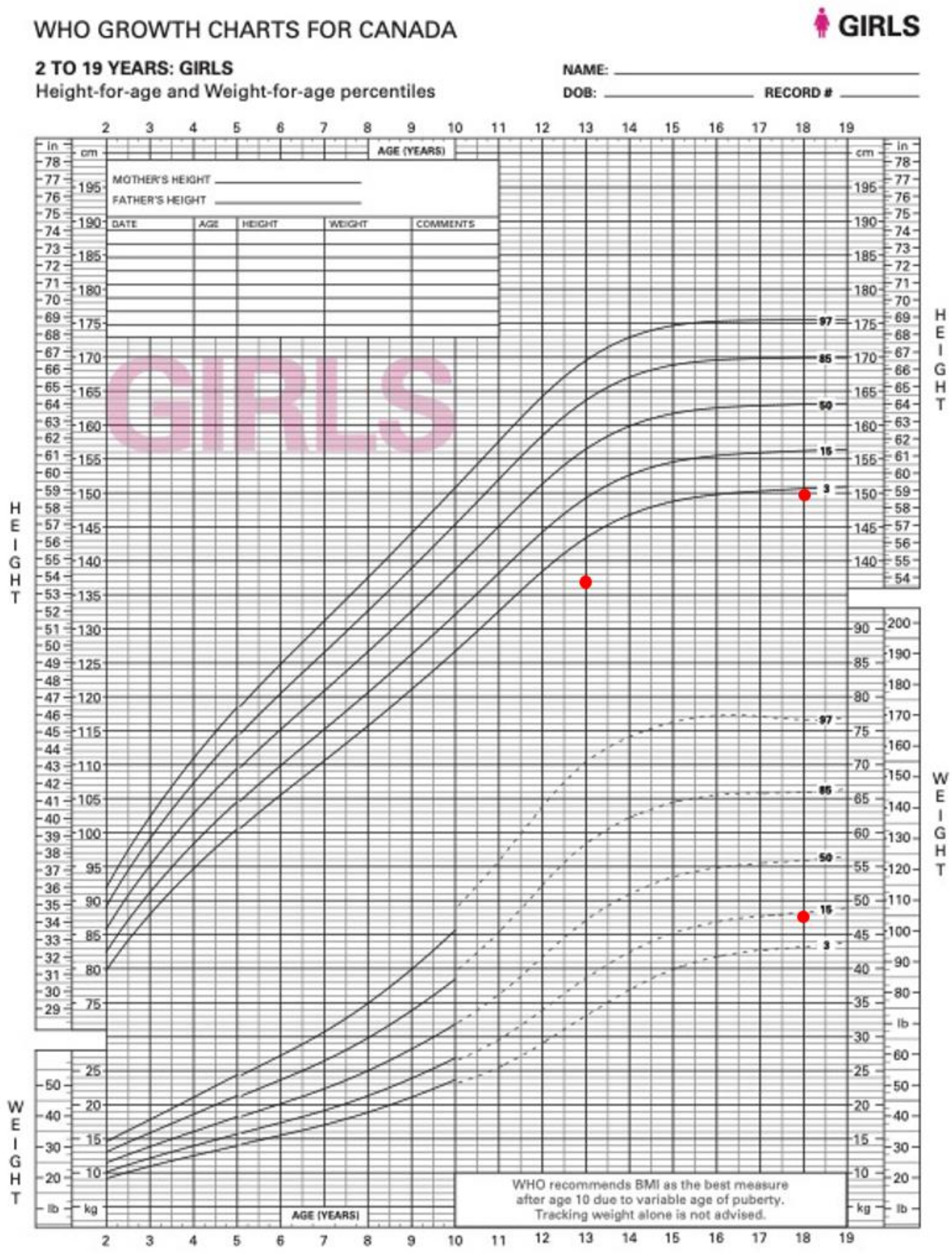

Figure 7: Height and weight of P1 marked in red dots on a World Health Organization (WHO) adapted growth charts for Canada using the 3rd, 15th, 50th, 85th, and 97th percentiles for girls aged 2-19 years.

Patient 2

Patient 2 (P2) is a 16-year-old female who has had struggles with severe atopic disease, recurrent infection, and gastrointestinal disease from a very young age. P2 also has a growth chart that is consistent with her short stature (**Fig 8**). She was born to non-consanguineous parents of Hispanic decent. She has no full siblings, but does have 4 paternal half siblings and one maternal half-sibling. Her father and 2 paternal half-siblings have asthma; one paternal half sibling has eczema. She presented with severe reflux leading to hospitalization within the first month of life and was diagnosed with pyloric stenosis at that time. At the same age she was first noted to have atopic dermatitis, as well as concern for IgE-mediated food allergy. At 5 months of age she had anaphylaxis, presumed to be from egg in breastmilk. Radioallergosorbent (RAST) testing at the time demonstrated the presence of IgE-against milk, egg, rice, turkey, wheat, soy, and peanut. Due to her multiple food allergies she was nutritionally supported with hypoallergenic amino acid-based formula. She was diagnosed with eosinophilic esophagitis at 3 years of age with histological evidence of eosinophils in the esophagus (25 eosinophils per high powered field (HPF)) and increased lamina propria of the stomach.

Her atopic dermatitis, present since the 1st month of life, was frequently associated with bacterial superinfection-driven flares of disease. Management of her eczema with mid-high potency topical steroids and topical antimicrobials initially provided some improvement. However, her atopic dermatitis worsened with age and became less responsive to topical therapies. At 10 years of age, methotrexate was added but was not tolerated due to gastrointestinal side-effects. At 13 years of age she was started on dupilumab therapy, which was associated with decreased symptoms, particularly her itchiness and over the subsequent following years, reduction in her cutaneous bacterial infections. Specifically, her Investigator's Global Assessment Scale (IGA) fell from 4/4 (severe) to 1/4 (almost clear). Dupilumab therapy was also associated with an ability to discontinue swallowed budesonide with continued control of her eosinophilic esophagitis to 5-10 eosinophils per HPF within a year of initiation of this biologic.

By 2 years of life she was diagnosed with asthma, which was managed with inhaled corticosteroids and monteleukast. However, she had recurrent flares associated with underlying infections, and innumerable hospitalizations in the first 12 years of life.

With regard to infection, she had 5 episodes of hospitalization for pneumonia/bronchitis in the first year of life, with 2 episodes requiring intubation and mechanical ventilation. She has had multiple episodes of cutaneous abscesses, including perianal boils. At 8 years she developed challenges with recurrent HSV with ocular involvement and she has been sporadically treated for non-disseminated flat warts. Given the severity of the disease, WES was conducted and a *de novo* heterozygous variant for *STAT6* (NM_001178079.2) at c.1256A>C, p.D419A was identified.

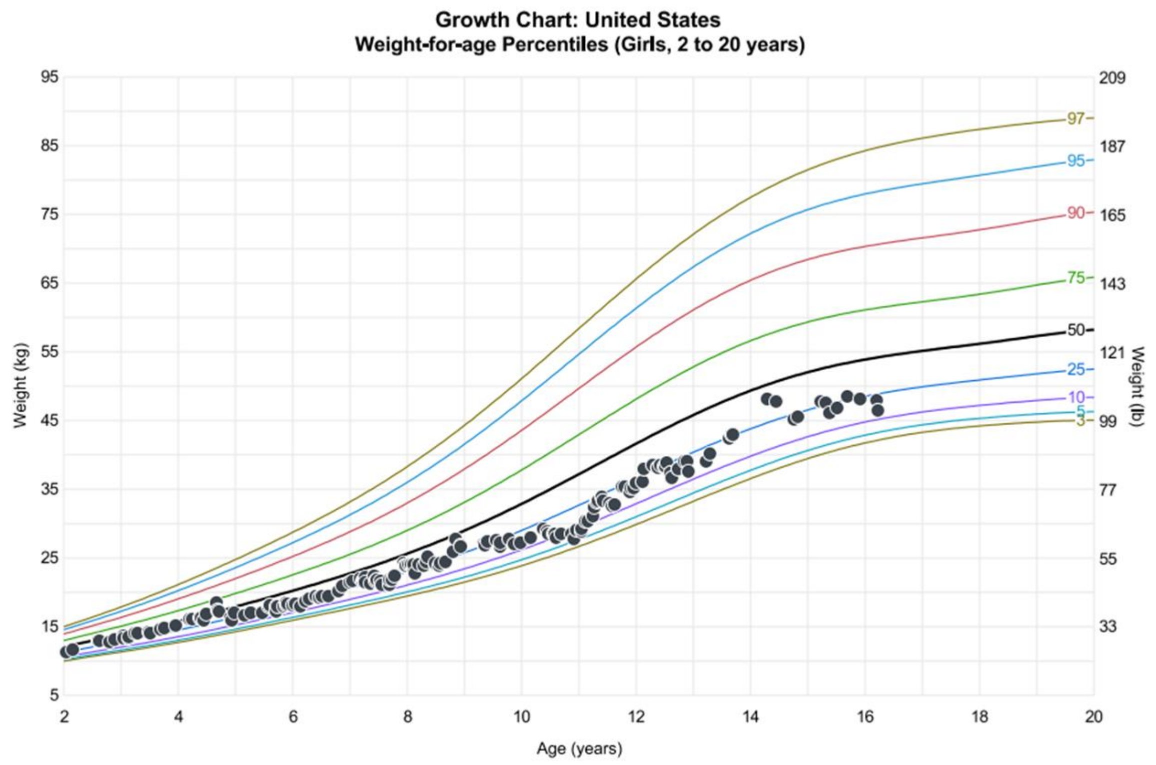
Growth Chart: United States
Weight-for-age Percentiles (Girls, 2 to 20 years)
Weight (kg)
Weight (lb)
Age (years)
97
95
90
75
50
25
10
5
3

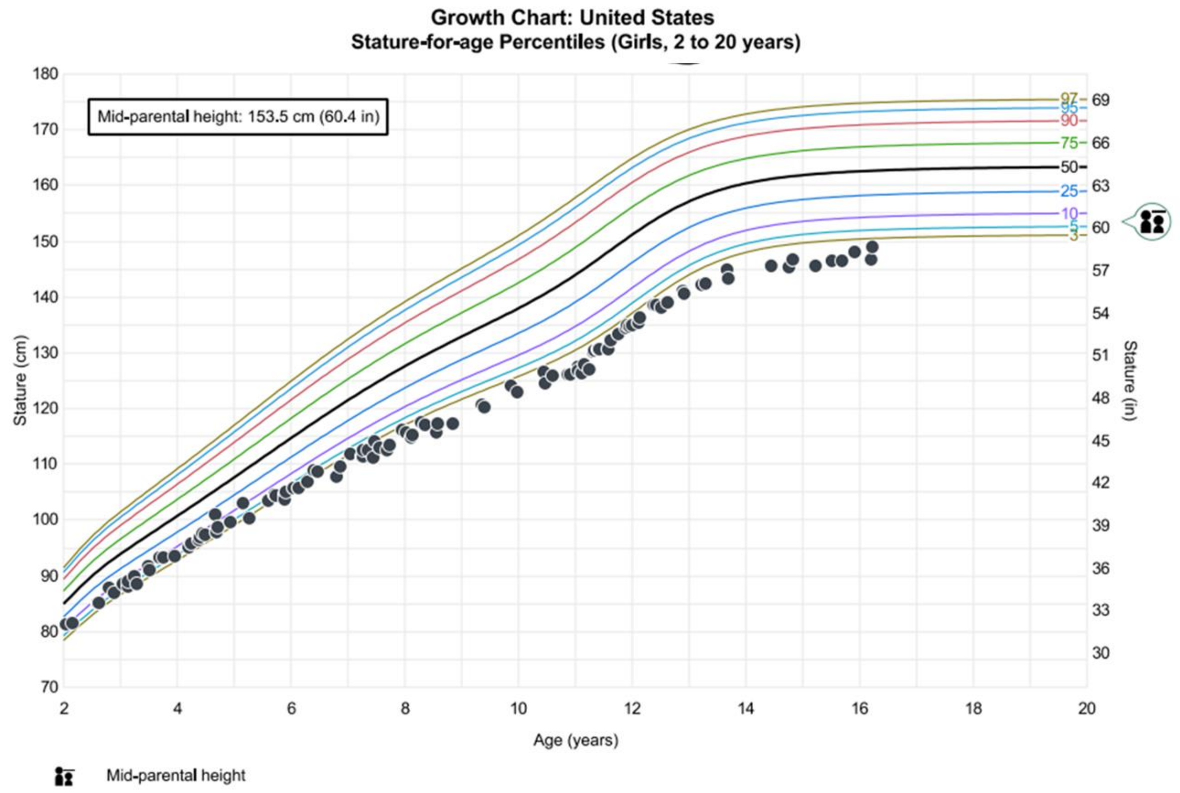
Growth Chart: United States
Stature-for-age Percentiles (Girls, 2 to 20 years)
Mid-parental height: 153.5 cm (60.4 in)
Stature (cm)
Stature (in)
Age (years)
97
95
90
75
50
25
10
5
3
Mid-parental height

Figure 8: Height and weight of P2 marked in black dots on United States specific growth charts for girls aged 2-20 years. Percentiles 3rd, 5th, 10th, 25th, 50th, 75th, 90th, 95th, 97th are shown for reference.

Patient 3

Patient 3 (P3) is a 37-year-old female of Middle Eastern origin who is the mother of Patient 4. She was born to unrelated parents, and she has six siblings. Neither the parents nor her siblings suffered with allergic disease. She developed severe atopic dermatitis during childhood. She was admitted to hospital twice at 2 and 14 years when her skin condition became so severe that she required inpatient atopic dermatitis management. She had no neurologic or skeletal features. Currently, she has chronic xerosis, cutaneous itching, and some food allergies. Blood testing revealed eosinophilia and elevated serum IgE levels.

Patient 4

Patient 4 (P4) is a 10-year-old male of Middle Eastern origin who is the son of P3. His parents are unrelated, and both his father and younger female sibling are healthy with no significant allergic disease. He presented at 1.5 months of age with moderately severe atopic dermatitis that persisted throughout childhood. He also presented with low growth velocity that has carried on throughout his life (**Fig 9**). Furthermore, he developed recurrent upper and lower respiratory infections (mostly viral) since early infancy. He had no diagnosis of asthma and received no inhalational therapies. Monthly intravenous immunoglobulin started at age 7 years and ameliorated respiratory infections. He has clinical IgE-mediated food allergy with episodes of anaphylaxis occurring after eating peanut and pistachio during the second year of life. Allergen skin prick test is positive for peanuts, tree nuts, eggs, and fish. Microarray component resolved diagnosis (CRD) analysis confirmed high specific IgE titers to several food and aeroallergens. Beginning at age 2 years, he experienced dysphagia and esophageal food impaction. Endoscopy visualized stenosis of the esophagus and biopsies confirmed the diagnosis of eosinophilic esophagitis. The six-food elimination diet designed for eosinophilic oesophagitis and swallowed fluticasone were of limited efficacy. He is currently managed with a combination of oral prednisolone (unable to wean below 5 mg per day), a proton pump inhibitor and he undergoes balloon dilatation of the esophagus every month. He is on regular emollient and topical mometasone to control atopic dermatitis. P4 experiences recurrent oropharyngeal and esophageal candidiasis which responds to fluconazole therapy. P4 has some skeletal features including delay in closure of anterior fontanelle until age 3 years, fracture of right ankle bones at age 4 years, and ectopic eruption of an upper incisor tooth at age 7 years. He has mild coarse facies and no joint hyperlaxity. Bone densitometry shows no osteopenia. Magnetic resonance

imaging (MRI) and angiography (MRA) of the brain are unremarkable. Serial blood testing is notable for eosinophilia and elevated serum IgE levels. P4 had received tofacitinib (5mg/day) for 2 months at the time this manuscript was finalized. His initial response to tofacitinib was encouraging with less dysphagia, less esophageal food impaction, and improved endoscopic appearance of the esophagus.

Genetic testing, via WES, revealed a heterozygous variant for STAT6 (NM_001178079.2) at location c.1255G>T, p.D419Y in P4, that was maternally inherited from P3.

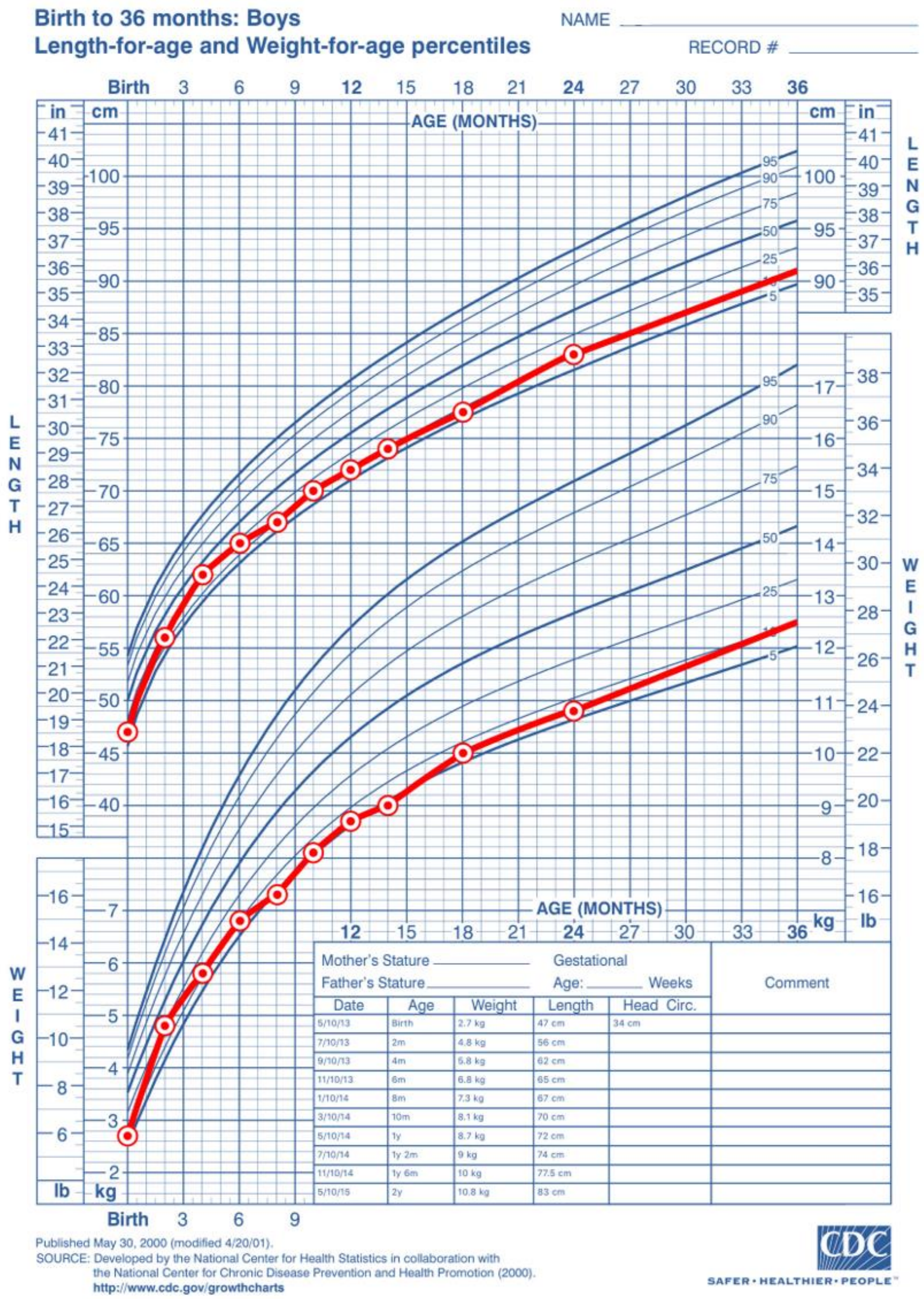
Birth to 36 months: Boys
Length-for-age and Weight-for-age percentiles
NAME
RECORD #
AGE (MONTHS)
LENGTH
WEIGHT
Mother's Stature
Father's Stature
Gestational Age: Weeks
Comment
Date Age Weight Length Head Circ.
5/10/13 Birth 2.7 kg 47 cm 34 cm
7/10/13 2m 4.8 kg 56 cm
9/10/13 4m 5.8 kg 62 cm
11/10/13 6m 6.8 kg 65 cm
1/10/14 8m 7.3 kg 67 cm
3/10/14 10m 8.1 kg 70 cm
5/10/14 1y 8.7 kg 72 cm
7/10/14 1y 2m 9 kg 74 cm
11/10/14 1y 6m 10 kg 77.5 cm
5/10/15 2y 10.8 kg 83 cm
Published May 30, 2000 (modified 4/20/01).
SOURCE: Developed by the National Center for Health Statistics in collaboration with the National Center for Chronic Disease Prevention and Health Promotion (2000).
http://www.cdc.gov/growthcharts
SAFER · HEALTHIER · PEOPLE

Figure 9: Height and weight of P4 marked in red circular dots on Center for Disease Control (CDC, US) specific growth charts for boys from birth to 3 years of age. Percentiles 5th, 10th, 25th, 50th, 75th, 90th, 95th are shown for reference.

Patient 5

Patient 5 (P5) is a 22-year-old female of European descent born at term after an uneventful pregnancy to healthy, non-consanguineous parents. She has two younger monozygotic twin brothers who are healthy without allergic disease. In the first year of life she developed severe widespread atopic dermatitis refractory to topical therapies and she experienced several episodes of anaphylactic shock due to multiple food allergies. Her sensitization to food as confirmed by high levels of specific IgE has grown over time resulting in a very restricted diet. She has documented IgE sensitization by radioallergosorbent (RAST) test or immuno-solid phase allergen chip (ISAC) test to many allergens (milk, eggs, shellfish, fish, blueberry, dried fruits, legumes, soy). She has also experienced allergic reactions to some drugs.

As a child she experienced recurrent respiratory infections, including two episodes of bacterial pneumonia. At the age of 2 years she was diagnosed with severe asthma which persists today and is managed with a combination of oral antihistamines, inhaled corticosteroids, and inhaled long-acting beta 2-agonists (LABAs). Asthma flares are managed with courses of oral corticosteroids and inhaled short-acting beta 2-agonists (SABAs), and recurrent infections are managed with antibiotics. A chest CT scan, performed at 22 years, showed widespread bronchiectasis and signs of chronic and recurrent infections/inflammation of the parenchyma. No invasive fungal infections have ever been detected, but she experienced recurrent vaginal candidiasis. Chronic low-copies EBV blood replication with complete seroconversion has also been documented.

From the age of 15 years, she experienced irregular bowel habits with abdominal pain, constipation and diarrhoea. The endoscopic biopsy, performed at the age of 20 years while under the exclusion diet, showed a non-specific infiltrate with an eosinophilia (3-5 per high power field (HPF) in the esophageal mucosa and 10-33 per HPF in the large intestine.

Addition features include: (a) she presented at the age of 3 years with HPV-negative oral and laryngeal papillary hyperplasia; (b) she has features of hyperextensible joints; (c) a homozygous mutation in the *MTHFR* gene was detected, associated with hyperomocysteinemia that improved with folic acid therapy; (d) brain MRI performed at the age of 22 years which was unremarkable; (e) and normal growth velocity over the course of her life (**Fig 10**).

Serial blood testing was notable for eosinophilia and persistently elevated serum IgE values. She was noted to have a reduced frequency of peripheral Th17 cells as well as a reduction

overtime of the frequency and absolute values of CD19 B-cells with a normal pattern of B cell maturation. Next generation sequencing (NGS) panel was nagtive for known IEI genes, however clinical exome sequencing identified a de novo heterozygous variant in *STAT6* (NM_001178079.2) at location c.1255G>A; p.D419N.

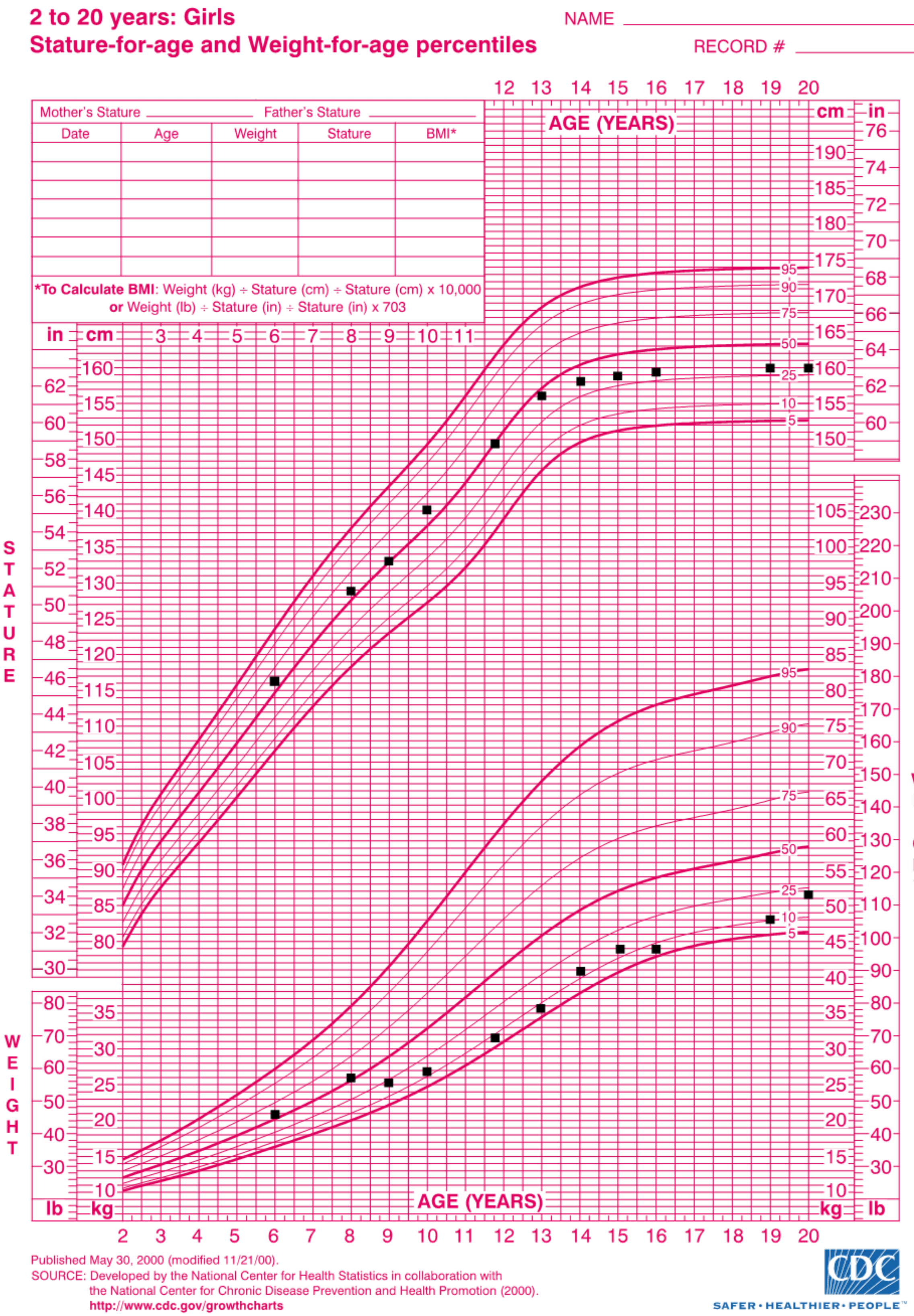
2 to 20 years: Girls
Stature-for-age and Weight-for-age percentiles
NAME
RECORD #
Mother's Stature
Father's Stature
Date
Age
Weight
Stature
BMI*
*To Calculate BMI: Weight (kg) ÷ Stature (cm) ÷ Stature (cm) x 10,000
or Weight (lb) ÷ Stature (in) ÷ Stature (in) x 703
AGE (YEARS)
STATURE
WEIGHT
in
cm
lb
kg
95
90
75
50
25
10
5
Published May 30, 2000 (modified 11/21/00).
SOURCE: Developed by the National Center for Health Statistics in collaboration with
the National Center for Chronic Disease Prevention and Health Promotion (2000).
http://www.cdc.gov/growthcharts
CDC
SAFER • HEALTHIER • PEOPLE™

Figure 10: Height and weight of P5 marked in black squares on CDC specific growth charts for girls from 2 to 20 years of age. Percentiles 5th, 10th, 25th, 50th, 75th, 90th, 95th are shown for reference.

Patient 6

Patient 6 (P6) is a male patient of East Asian descent with severe treatment-resistant atopic dermatitis, asthma and multiple food allergies since early days of life, and required frequent hospital admissions for management of severe atopic dermatitis. At the age of 8 years, he had an episode of bacteremia with pancytopenia due to *Staphylococcus aureus* and group G *Streptococcus*. Other notable infections include recurrent *S. aureus* cellulitis and *S. aureus* septic arthritis in hip and knee. At the age of 9 years, he was diagnosed with eosinophilic gastrointestinal disease with biopsy-proven hyper eosinophilia in stomach and duodenum. This eosinophilic gastrointestinal disease resulted in protein-losing enteropathy and multiple nutrient deficiencies. At the age of 14 years, the patient experienced pulmonary hypertension and was diagnosed with interstitial lung disease during an asthmatic attack accompanied by severe eosinophilia. The patient has short stature with his height was persistently lower than 3rd percentile since young childhood until young adulthood accompanied by delayed bone age (**Fig 11**). He was started on growth hormone replacement at age of 16 years, but did not alter his poor growth velocity. Subsequent to the diagnosis of STAT6 gain-of-function disease, he was started on dupilumab at the age of 19 years, and has enjoyed a dramatic reduction of peripheral eosinophilia, marked improvement atopic dermatitis activity as quantified by SCORAD and EASI scores, and most importantly, the sustained absence of disease flare. The patient also experienced rapid growth after initiating dupilumab treatment, with close to 20cm height gain and 30kg weight gain in the 2 years after his first dose of dupilumab. Given the severity of the disease presentation, WES was conducted and a *de novo* heterozygous variant at c.1256A>G, p.D419G for *STAT6* (NM_001178079.2) was identified.

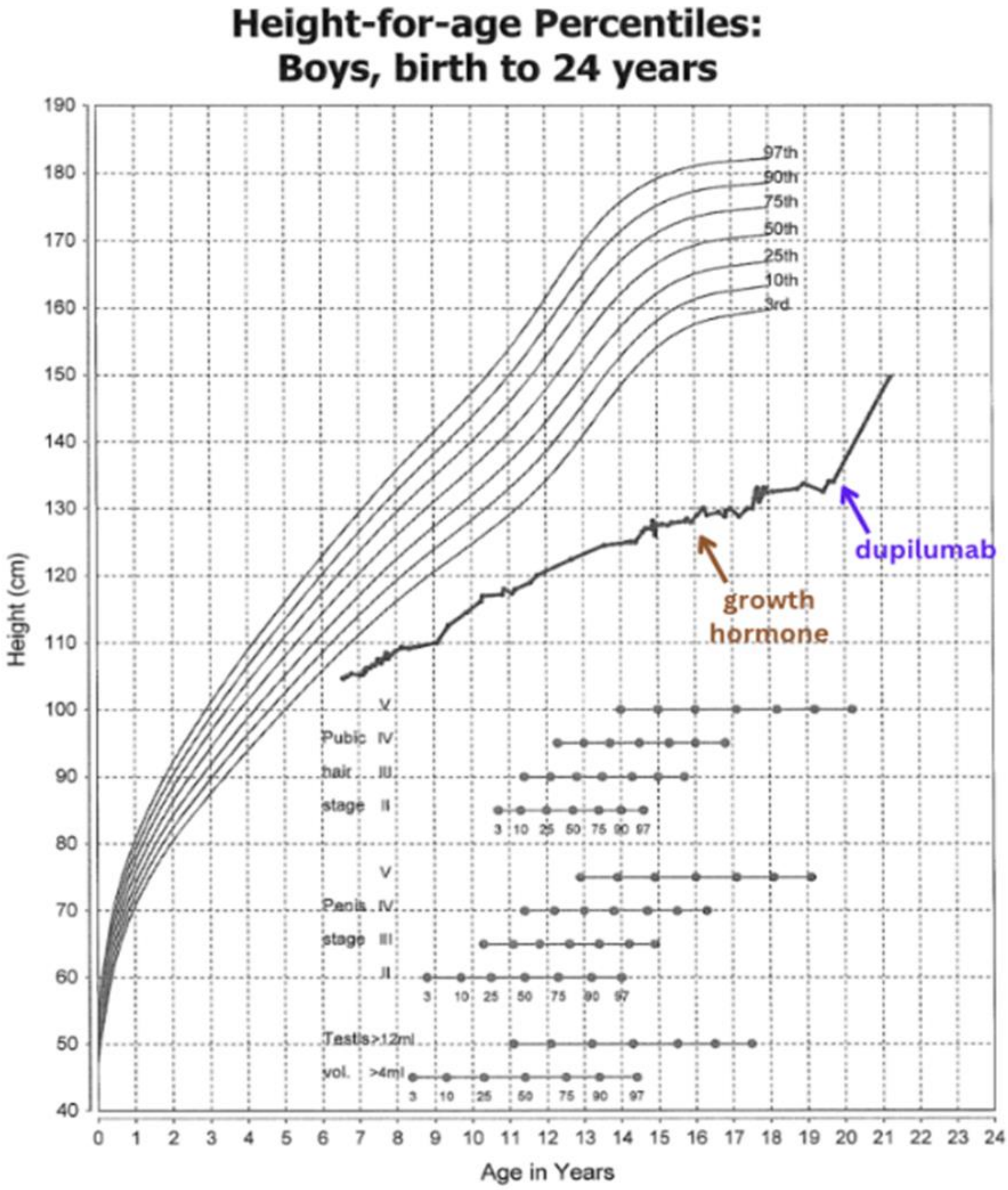
Height-for-age Percentiles:
Boys, birth to 24 years
Height (cm)
Age in Years
97th
90th
75th
50th
25th
10th
3rd
growth hormone
dupilumab
Pubic hair stage
Penis stage
Testis vol.
>12ml
>4ml

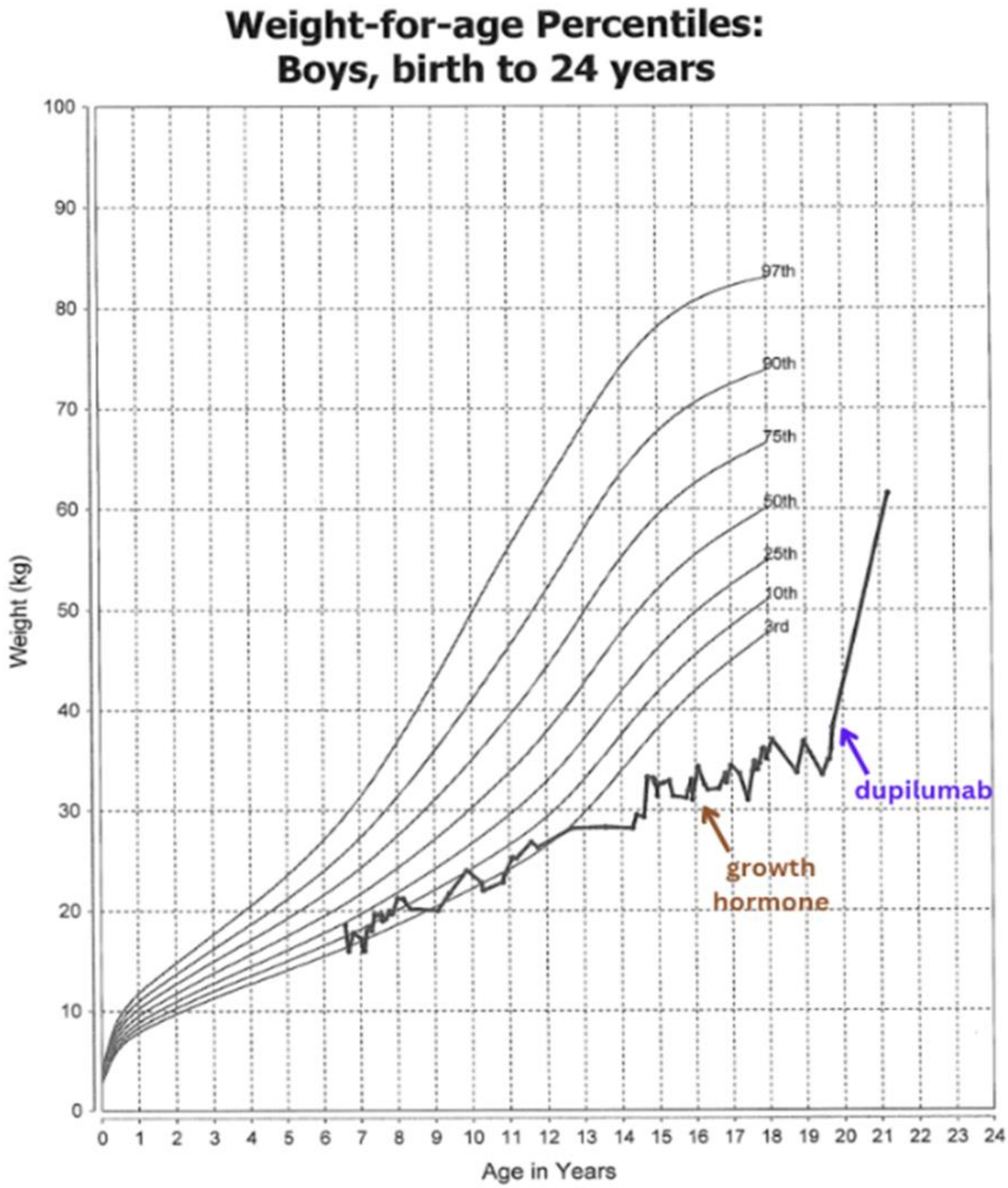

Figure 11: Height and weight growth charts for P6 (black line) in reference to the 3rd, 10th, 25th, 50th, 75th, 90th, 97th percentile of the Hong Kong population for boys from birth to 24 years of age. Initiation of growth hormone treatment and Dupilumab treatment are shown on the graphs.

Patients 7-9 are part of a family that are also described in the following preprint: "Autosomal dominant STAT6 gain of function causes severe atopy associated with lymphoma" (https://doi.org/10.21203/rs.3.rs-2116300/v1).

Patient 7

Patient 7 (P7) is a 60 year old female of European descent presented in early childhood with severe atopic dermatitis, multiple food allergies, and frequent asthma exacerbations as a child. She was also found to have high serum IgE levels and clinical food allergies to nuts, shellfish, and cow's milk. Her asthma and atopic dermatitis symptoms ameliorated throughout adolescence, although she continues to have allergic symptoms, which are managed with a combination of inhaled and topical steroids. She was diagnosed with eosinophilic esophagitis at age 26 years which was responsive to topical steroid therapy. She is the mother of 3 children, two of whom had severe allergic disease (Patients 8 and 9). At age 49 years she was diagnosed with follicular lymphoma at following presentation with bilateral axillary lymphadenopathy. She subsequently relapsed with diffuse large B cell lymphoma (DLBCL) at age 60 years and is currently receiving standard chemotherapy protocols.

Patient 8

Patient 8 (P8) is a 42 year old male and son of P7. He presented in his first month of life with severe widespread atopic dermatitis, and recurrent pneumonia at ages 6 and 12 months. At this age he had a serum IgE >5000 kU/L (over detection limit at the time). In his second year of life, he developed food to multiple allergens including: cow's milk, soy, and most tree nuts. Later in childhood is developed more food allergies to include chicken, sesame, cod, salmon, and tuna. He has had 4 episodes of anaphylaxis requiring resuscitation, all thought to be due to accidental exposure to food allergens. He was diagnosed with asthma at age 4 years and had frequent exacerbations throughout childhood, requiring multiple hospital admissions for treatment with nebulised and intravenous bronchodilators. He has seasonal allergic rhinoconjunctivitis with documented IgE-mediated sensitisation to grass and tree pollen. He was diagnosed with eosinophilic esophagitis at 36 years of age after presenting with dysphagia and was treated with oral budesonide. Currently at 42 years old, his eczema and asthma symptoms are stable. He is treated with inhaled steroids and topical emollients and requires occasional topical steroid use.

Patient 9

Patient 9 (P9) is the son of P7 and younger brother of P8. P9 died of anaphylaxis at age 20 years. Details are limited, but by family report, P9 had severe atopic disease manifestations from early childhood including atopic dermatitis, asthma with frequent exacerbations, and multiple food allergies including to cow's milk, nuts, soy, and shellfish. He died of anaphylaxis at age 20 years following ingestion of the non-steroidal anti-inflammatory drug ibuprofen.

Next generation sequencing identified a heterozygous *STAT6* (NM_001178079.2) at location c.1255G>C, p.D419H variant for P9 and P8 that was maternally from P7.

Patient 10

Patient 10 (P10) was a male patient who had life-long severe allergic disease and short stature (**Fig 12**). The patient was not dysmorphic and was born to unrelated parents. Family history was notable, with the mother having experienced mild allergies, eczema, and asthma, and the father had eczema. There is no family history of consanguinity. From 2 months of age, P10 developed widespread, treatment-resistant atopic dermatitis (eczema), gastroesophageal reflux with episodes of aspiration pneumonia, as well as chronic diarrhea. He underwent Nissen fundoplication at age 6 months (with a surgical revision at age 6 years) and gastrostomy feeding was commenced at age 15 months. Over time, he was also diagnosed with multiple food and drug allergies with anaphylactic reactions (combined with positive skin prick testing or RAST testing) documented in response to peanuts, cows milk protein, bananas, sesame seeds, fish, and eggs. He was fed with extensively hydrolyzed and elemental formulas via gastrostomy with very limited oral feeding for much of his life. He was also diagnosed with asthma. He experienced episodes of secondary bacterial skin infections from which *Staphylococcus aureus*, group A streptococci and *Candida* spp. were isolated. Widespread *molluscum contagiosum* was another notable skin finding. In his second and third decade of life, he had persisting eczema, asthma, and perennial rhinitis which worsened during pollen seasons. Repeated upper GI endoscopies confirmed the diagnosis of eosinophilic esophagitis. During this time, most of his feeding consisted of elemental formula via gastrostomy supplemented with limited oral intake (mainly rice, potatoes, and chicken). He was treated with oral steroids for many years and experienced symptom flares whenever the dose of prednisone was dropped below 10mg/day. He developed side-effects related to corticosteroids including cataracts, osteoporosis, and pathogenic fractures. He had an episode of thromboembolic ischemia of the right first and third toes with pain and cyanosis, ultimately requiring a lumbar sympathectomy to relieve the pain. At age 35 years, he was hospitalized for non-bloody diarrhea, profound weight loss, malnutrition, weakness, and vertigo. He was treated with high dose of prednisone, antibiotics, and elemental feeding. During the hospitalization, he died after developing a spontaneous subarachnoid hemorrhage with obstructive hydrocephalus, presumed secondary to an aneurysm. Blood testing over the years confirmed eosinophilia and high serum IgE levels. At age 20 years, lymphocyte phenotyping and proliferation to a panel of mitogens were unremarkable. An initial genetic assessment revealed normal sequences for STAT3 and DOCK8, prompting a more extensive genetic evaluation by whole exome sequencing (WES). Subsequently, *de novo* heterozygous variant for *STAT6* (NM_001178079.2) at c.1144G>C, p.E382Q was identified.

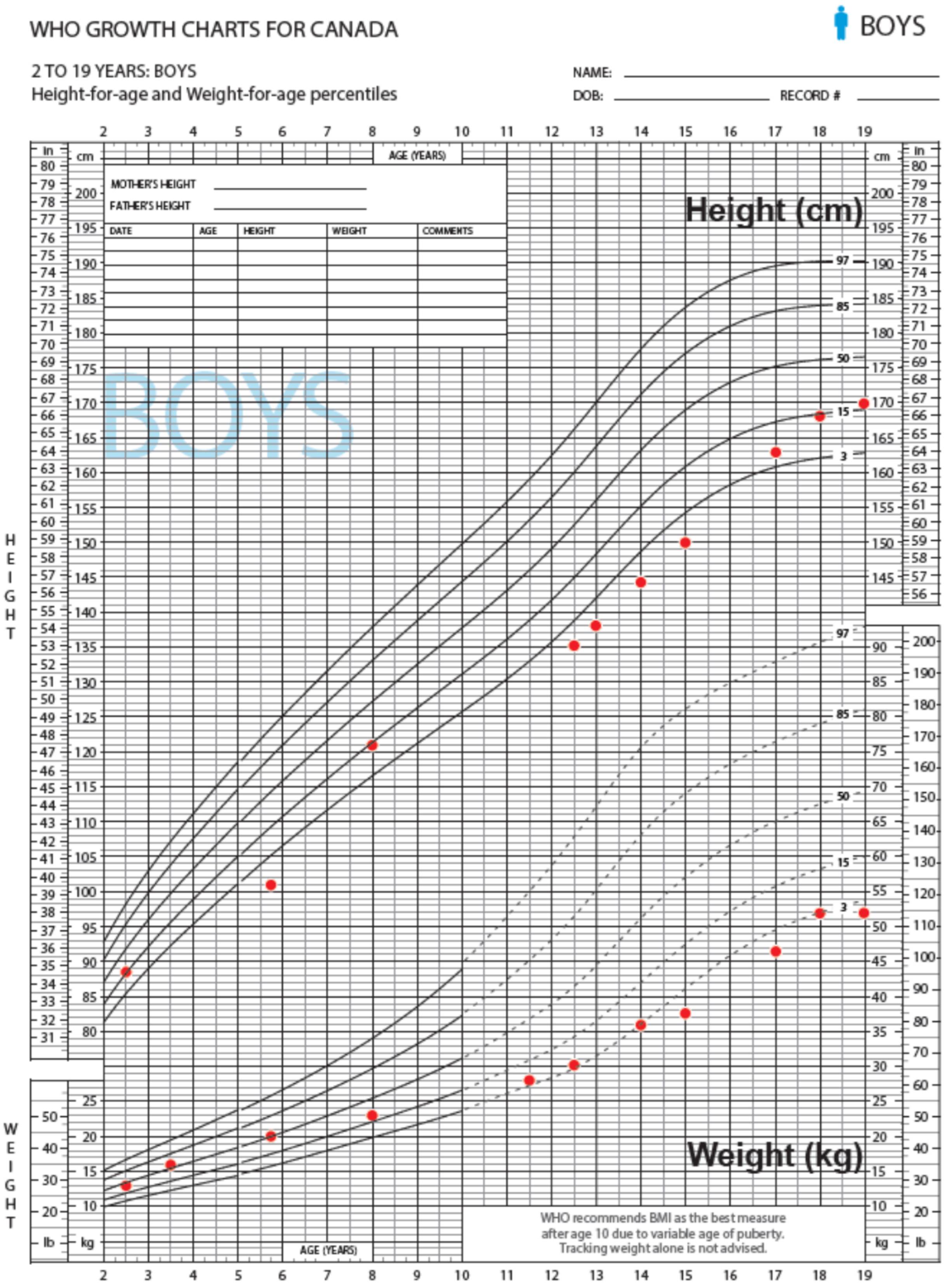
WHO GROWTH CHARTS FOR CANADA
BOYS
2 TO 19 YEARS: BOYS
Height-for-age and Weight-for-age percentiles
NAME:
DOB:
RECORD #
AGE (YEARS)
MOTHER'S HEIGHT
FATHER'S HEIGHT
DATE
AGE
HEIGHT
WEIGHT
COMMENTS
Height (cm)
Weight (kg)
HEIGHT
WEIGHT
WHO recommends BMI as the best measure after age 10 due to variable age of puberty. Tracking weight alone is not advised.

Figure 12: Height and weight of P10 marked in red dots on a WHO adapted growth charts for Canada using the 3rd, 15th, 50th, 85th, and 97th percentiles for boys aged 2-19 years.

Patient 11

Patient 11 (P11) is a 8-year-old male patient with severe atopic dermatitis beginning in early childhood complicated by recurrent bacterial skin infections as well as single episode invasive fungal skin infection. He is of South Asian descent with family history notable for a mother with mild atopic dermatitis and a maternal aunt with allergic rhinitis. He had a history of an infantile skin eruption beginning at four months of age. At age 6 years he was referred to a major pediatric academic center for treatment failure, and he was further escalated to a national referral center at age 8 years for treatment resistant atopic dermatitis. When reviewed at age 8 years, his atopic dermatitis remained severe (SCORAD=57) despite intense therapy with high potency topical corticosteroids and topical tacrolimus. When assessed at 8 years, in addition to his severe atopic dermatitis, notable findings were short stature (**Fig 13**) and laboratory evaluation revealing eosinophilia and elevated serum IgE levels. He was treated with multiple modalities including topical and oral corticosteroids, oral anti-fungal, topical and oral antibiotics and topical calcineurin inhibitors. He experienced cutaneous infections with fungal superinfection at age six years prompting treatment with oral griseofulvin, and an episode of bacterial superinfection at age six years treated with oral cephalexin Additional allergic features included allergic rhinitis with skin prick testing positive for dust mites possible drug allergy with urticaria following neomycin, bacitracin, and polymyxin ointment. WES was conducted and a de novo heterozygous variant for *STAT6* (NM_001178079.2) at c.1784A>G, p.K595R was identified.

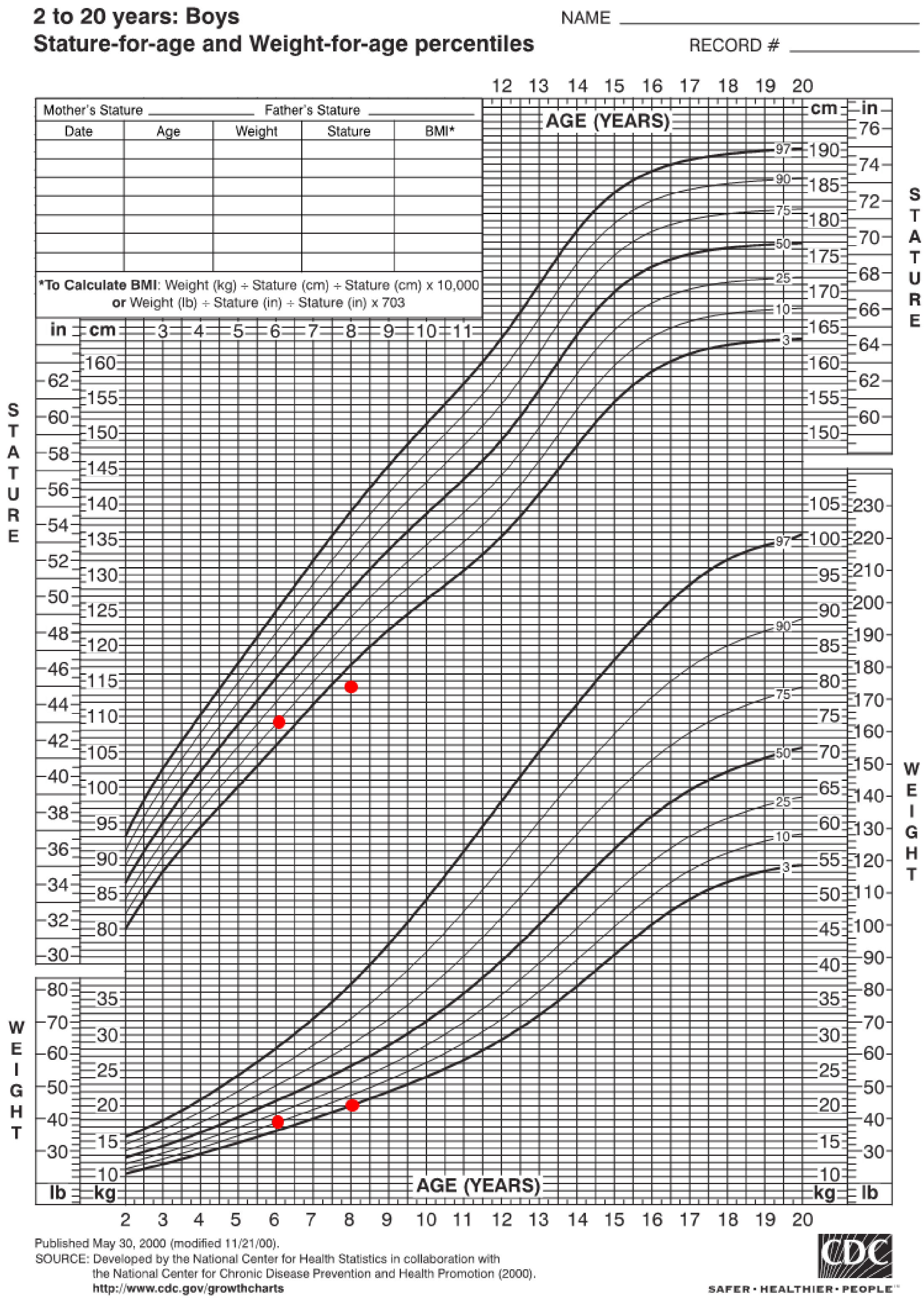
2 to 20 years: Boys
Stature-for-age and Weight-for-age percentiles
NAME
RECORD #
Mother's Stature
Father's Stature
Date
Age
Weight
Stature
BMI*
*To Calculate BMI: Weight (kg) ÷ Stature (cm) ÷ Stature (cm) x 10,000
or Weight (lb) ÷ Stature (in) ÷ Stature (in) x 703
AGE (YEARS)
STATURE
WEIGHT
in
cm
lb
kg
Published May 30, 2000 (modified 11/21/00).
SOURCE: Developed by the National Center for Health Statistics in collaboration with
the National Center for Chronic Disease Prevention and Health Promotion (2000).
http://www.cdc.gov/growthcharts
CDC
SAFER · HEALTHIER · PEOPLE

Figure 13: Height and weight of P11 marked in red dots on CDC specific growth charts for boys from 2 to 20 years of age. Percentiles 3rd, 10th, 25th, 50th, 75th, 90th, 97th are shown for reference.

Patient 12

Patient 12 (P12) is a 5-year-old female of South East Asian descent with very early-onset uncontrolled asthma. The patient was born to healthy non-consanguineous parents and she has one healthy older sister. Within the first month of life she was noted to have dyspnea and was admitted to a local hospital for 2 weeks and was suspected to have pneumonia, yet microbiology workup was negative. Throughout the first year of life, the patient was admitted almost monthly for similar episodes of wheezing and dyspnea, occasionally with fever, with negative comprehensive microbiology work-up every time. She first required mechanical ventilation for her condition at 3 months old. She was diagnosed to have asthma and was treated with a combination of intermittent oral corticosteroids plus inhaled corticosteroids and inhaled bronchodilators. Until the age of 5 years, she has been hospitalized frequently requiring intubation and mechanical ventilation 13 times for asthma attacks. Extensive imaging ruled out a structural airway abnormality. Growth chart for P12 also shows short stature (**Fig 14**). Investigations revealed persistent eosinophilia and high IgE levels in blood. Trio WES of P12 and the two parents identified a *de novo STAT6* (NM_001178079.2) variant at c.1928C>G, p.P643R was identified.

Height-for-age GIRLS

2 to 5 years (percentiles)

World Health Organization

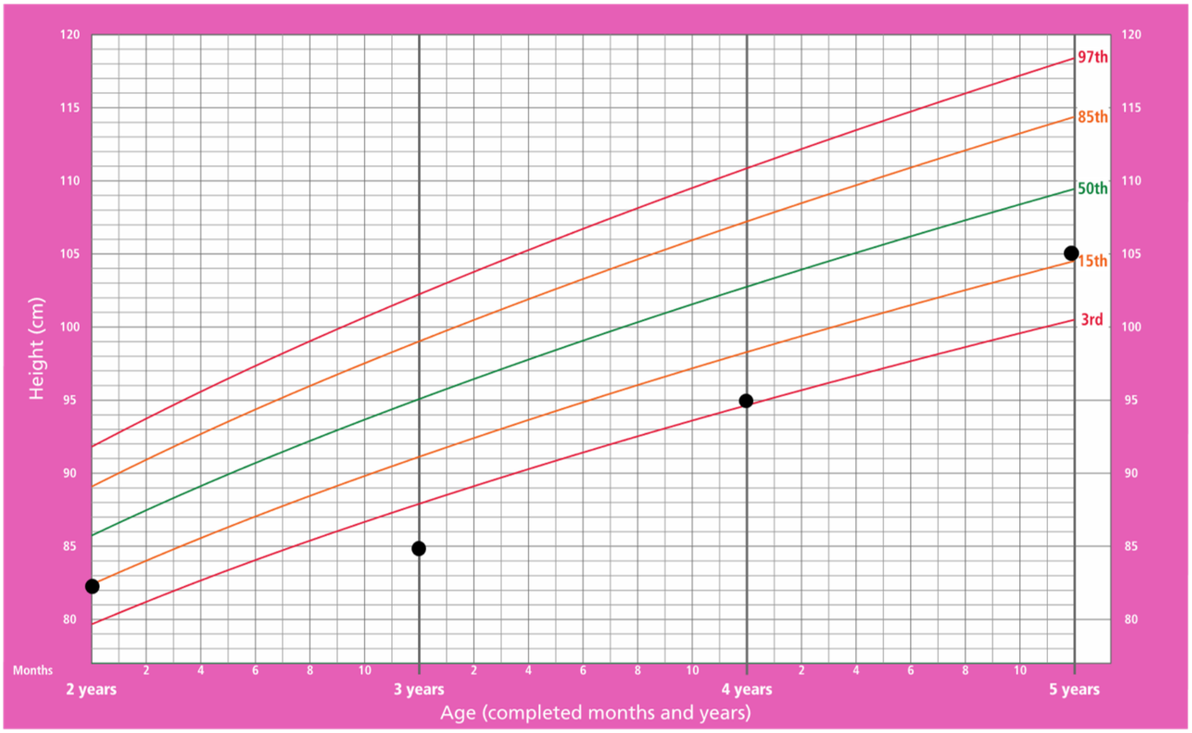

WHO Child Growth Standards

Weight-for-age GIRLS

2 to 5 years (percentiles)

World Health Organization

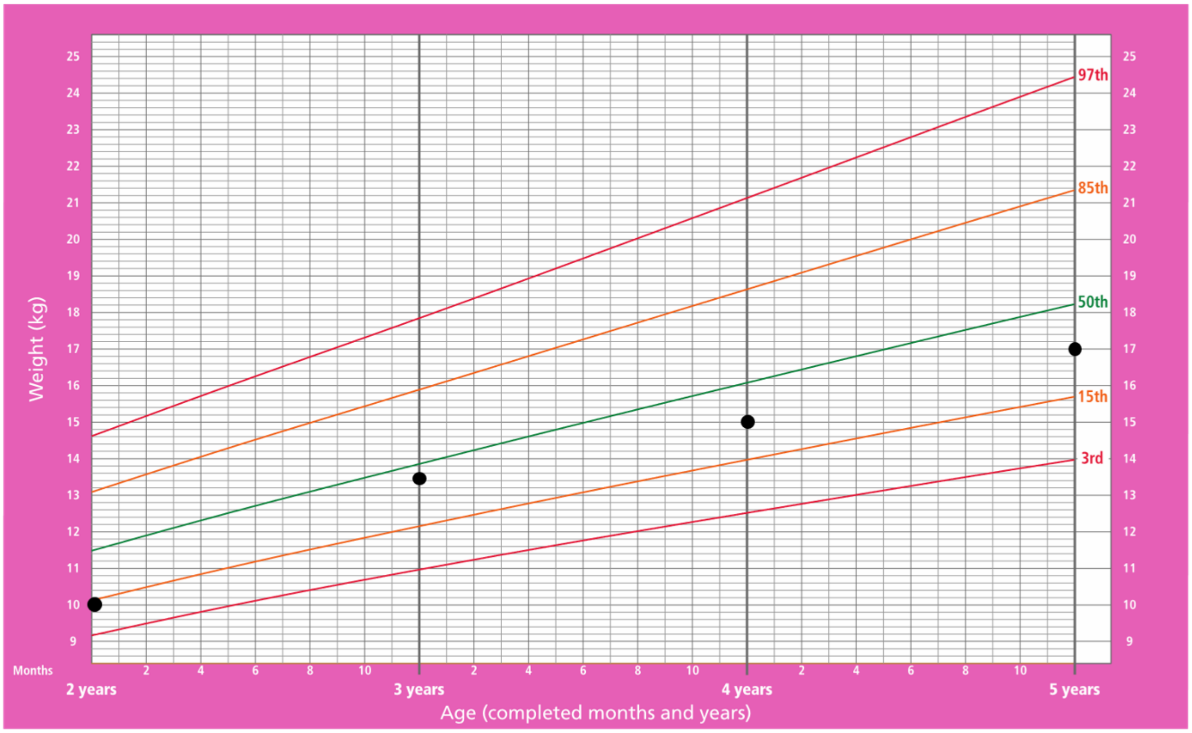

WHO Child Growth Standards

Figure 14: Height and weight of P12 marked in black dots on a WHO Child Growth charts using the 3rd, 15th, 50th, 85th, and 97th percentiles for girls aged 2-5 years.

Patient 13

Patient 13 (P13) is a 34-year-old female, born to non-consanguineous parents of Caucasian descent. Family history is notable for the fact that her father was reported to have suffered with severe atopic dermatitis, food allergies, and eczema although available details are limited. P13 is the mother of four children, three of whom have severe allergic disease (P14, P15 and P16 described below). From very early in childhood, she developed multiple IgE-mediated food allergies, asthma, and severe atopic dermatitis. She has documented food allergies to cow's milk, tuna, kiwi, tomato, egg, tree nuts and several stone fruits managed by life-long strict avoidance. Accidental exposures triggered anaphylactic reactions during childhood and again at age 34 years. Her atopic dermatitis flared around age 32 years requiring increased intensity therapy including PUVA (psoralen and UVA) treatment. At the age of 34 years she presented with a skin abscess which had been present for at least one year. The abscess was drained and the culture grew *Finegoldia magna*, a gram-positive anaerobic coccus, which responded to oral clindamycin. Serial blood testing revealed eosinophilia and a high total serum IgE level throughout her life. Next generation sequencing, mendeliome sequencing, was conducted on P13 and her four children and heterozygous variants at c.1555G>C, p.D519H in STAT6 (NM_001178079.2) were identified in the diseased individuals.

Patient 14

Patient 14 (P14), a 9-year-old male, is the second child born to a non-consanguineous couple of Caucasian descent. He was born term following a normal pregnancy and displayed no dysmorphic features. P14 has had low weight and height that has been 1 standard deviation below the average his entire life (**Fig 15**). His father died at age 27 years of septic shock with necrotizing pneumonia caused by influenza with secondary *Streptococcus pneumoniae* infection. Since the age of one month, he suffered from severe chronic atopic dermatitis. Over time, he was diagnosed with multiple IgE mediated food allergies (confirmed with positive skin prick testing or specific IgE (RAST) tests) including egg, wheat, gluten, rice, cow's milk, potato, peanut, tree nuts, codfish, stonefruit, corn, beef and chicken meat, kiwi, banana and celeriac. Upon exposure to these allergens, the patient's eczema flared and he experienced several anaphylactic reactions. Extensive dietary restrictions were required consisting of elemental formulas with very limited oral feeding that is still ongoing. He was diagnosed with eosinophilic esophagitis at the age of 3 years, which was treated oral viscous corticosteroids. Although initially responsive to oral corticosteroids, at age 6 years he was diagnosed with more extensive eosinophil gastrointestinal disease with eosinophilic gastritis and duodenitis requiring high dose systemic corticosteroids and nasgastric tube feeding with elemental formulas. In the ensuing years, eosinophilic intestinal disease activity frequently flared whenever his diet was expanded and complete control was never achieved despite treatment with systemic corticosteroids and high dose proton pump inhibitors. The patient was diagnosed with asthma at age 1 year and was treated with inhaled corticosteroids and bronchodilators. He was confirmed to have IgE-mediated sensitization to inhalant allergens including house dust mite, grass and tree pollen. He experienced several acute asthma exacerbations, often triggered by viral upper respiratory tract infections. The severity of the eosinophilic asthma and gastrointestinal disease prompted the initiation of treatment with mepolizumab (anti-interleukin-5 monoclonal antibody) at age 7yrs. Notably, hospitalization and systemic corticosteroid treatment was necessary on eight different occasions through his life, three of which were after the initiation of treatment with mepolizumab. At the age of 9 years and nine months, B-cell lymphopenia and hypogammaglobulinemia were identified and intravenous immunoglobulin replacement therapy was started. Serial blood testing revealed eosinophilia and a high total serum IgE level.

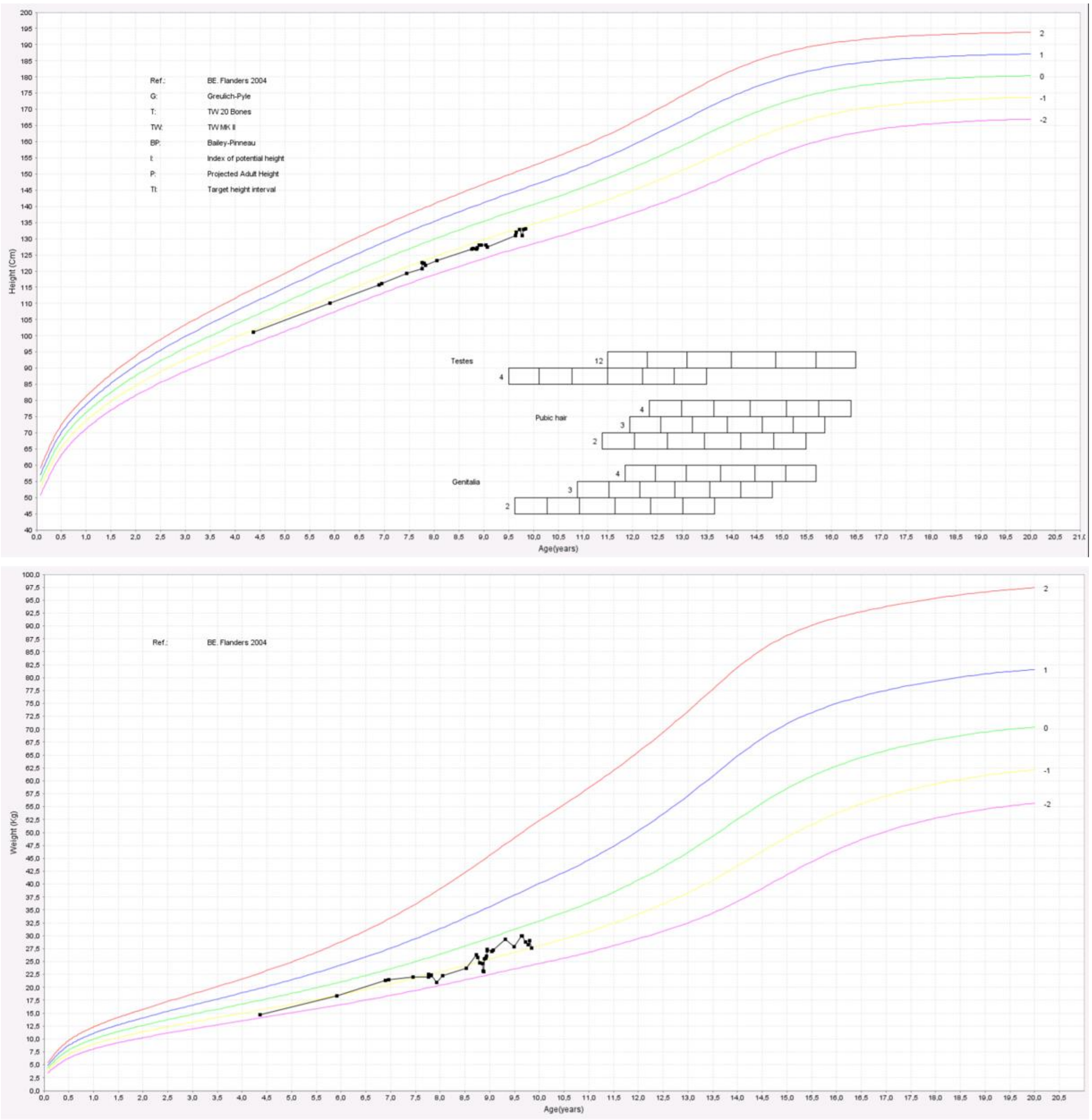

Figure 15: Height and weight growth charts for P14 (black lines) in reference to +/- 1 and +/- 2 standard deviation of the Flemish population of boys from birth to the age of 20 years.

Patient 15

Patient 15 (P15), an 8-year-old male, is the younger brother of P14. The patient was born term following a normal pregnancy and displayed no dysmorphic features. He has had normal weight and height trajectories (**Fig 16**). Analogous to his older brother, he presented with life-long severe atopic dermatitis. He was diagnosed with multiple, IgE mediated food allergies to egg, apple, wheat, soy, tomato, paprika, peanut and tree nuts. These allergies were confirmed with positive skin prick testing or specific IgE (RAST) tests. He experienced two anaphylactic reactions after eating chicken meat. The patient's diet was restricted to elemental formulas via nasogastric feeding with very limited oral feeding. At the age of 4 years he was diagnosed with eosinophilic esophagitis. He has subsequently suffered with relapsing eosinophilic oesophagitis, despite treatment with intense therapy with a combination of dietary restrictions, systemic and topical corticosteroids, and high dose high dose proton pump inhibitors. Serial blood testing revealed eosinophilia and a marked elevation of the serum total IgE level. B-cell lymphopenia and hypogammaglobinemia were detected at the age of 8 years and intravenous immunoglobulin replacement therapy was started.

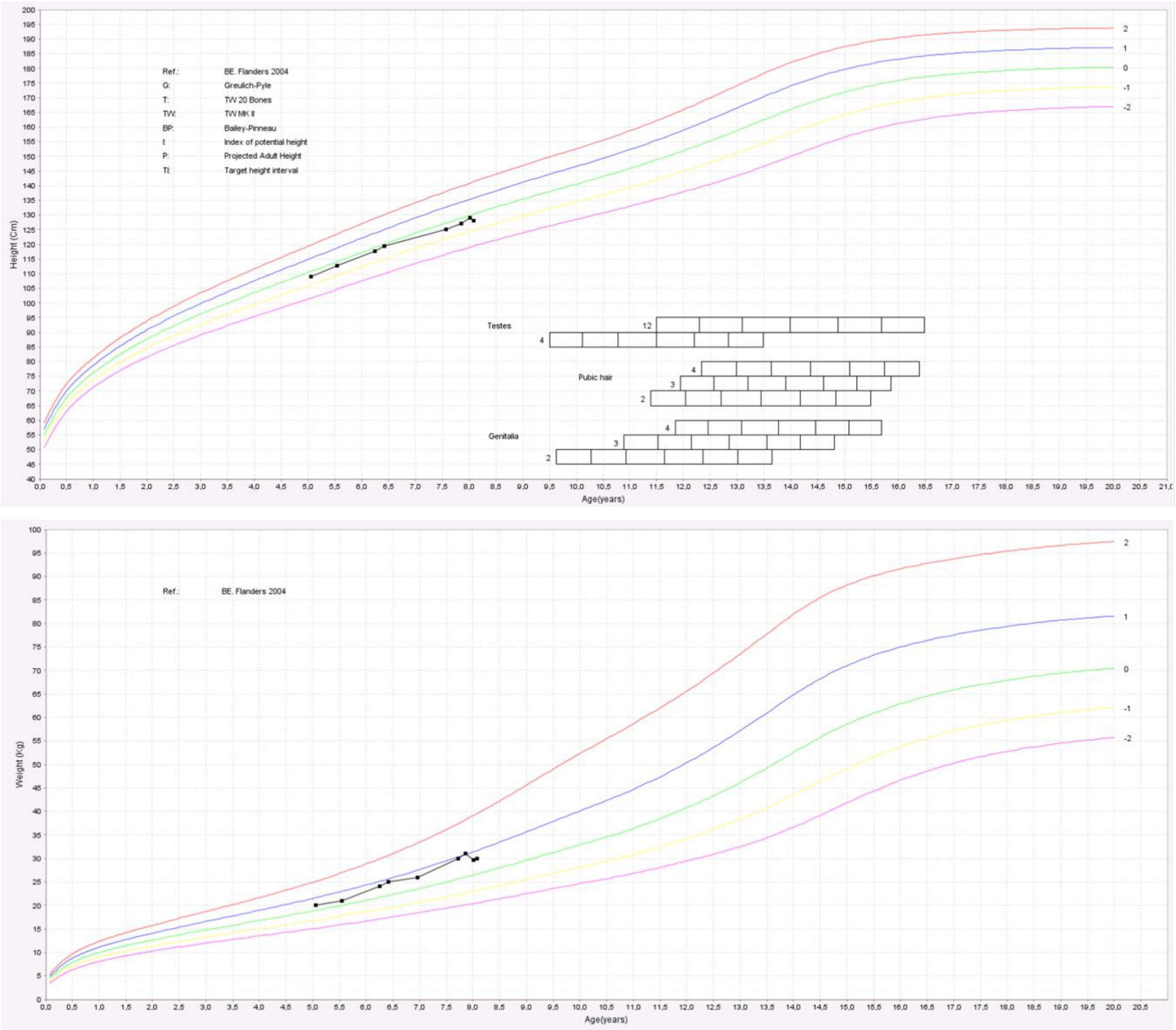

Figure 16: Height and weight growth charts for P15 (black lines) in reference to +/- 1 and +/- 2 standard deviation of the Flemish population of boys from birth to the age of 20 years.

Patient 16

Patient 16 (P16), a four-year-old female, is the younger sibling of P14 and P15. She was born term following a normal pregnancy and displayed no dysmorphic features. She also has a growth chart consistent with normal developmental trajectory (**Fig 17**). During the first year of her life, she developed severe atopic dermatitis, obstructive lung disease and multiple, IgE mediated food allergies. Skin prick testing and specific IgE (RAST) tests revealed sensitization to cow's milk, egg, potato, tree nuts, broccoli and cauliflower. Her diet was restricted to elemental formulas with limited oral feeding. At the age of two years she developed a first episode of eosinophilic oesophagitis. This was aggressively treated with high dose high dose proton pump inhibitors, systemic, and later oral viscous corticosteroids and the implementation of a six-food elimination diet designed for eosinophilic oesophagitis. Nevertheless, she experienced a flare of eosinophilic oesophagitis at the age of 4 years. She was also diagnosed with asthma at age 1 year requiring a 5-day hospitalization. Serial blood testing revealed eosinophilia and a high total serum IgE level. At the age of 4 years, her IgG level was considered at the lower border of the age-adjusted reference values while her B-cell count was normal.

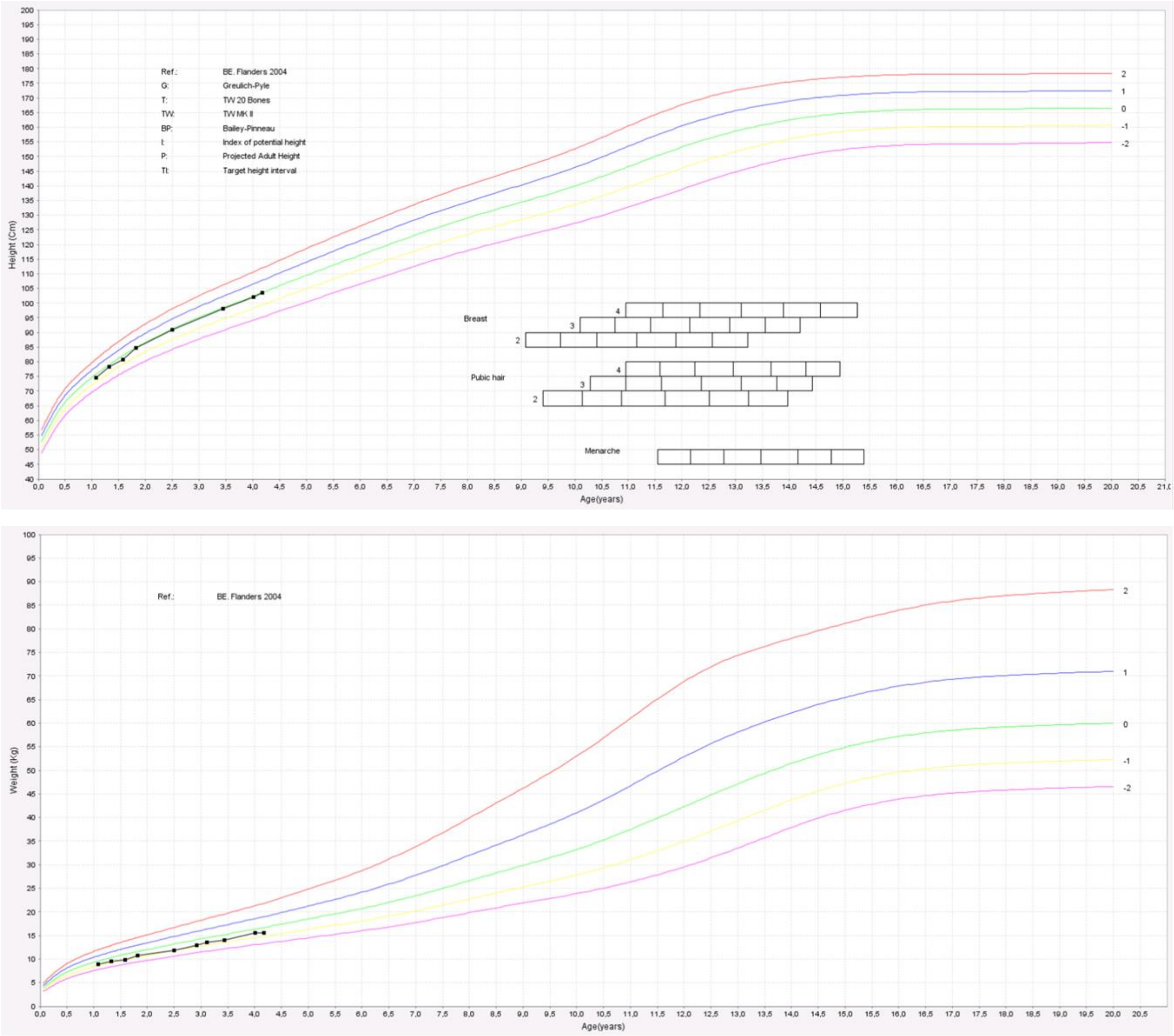

Figure 17: Height and weight growth charts for P16 (black lines) in reference to +/- 1 and +/- 2 standard deviation of the Flemish population of girls from birth to the age of 20 years.
