# Supplementary figures and images for: Human germline heterozygous gain-of-function STAT6 variants cause severe allergic disease
Source: J Exp Med. 2023 Mar 8;220(5):e20221755. doi: 10.1084/jem.20221755 (PMC10037107; doi:10.1084/jem.20221755)

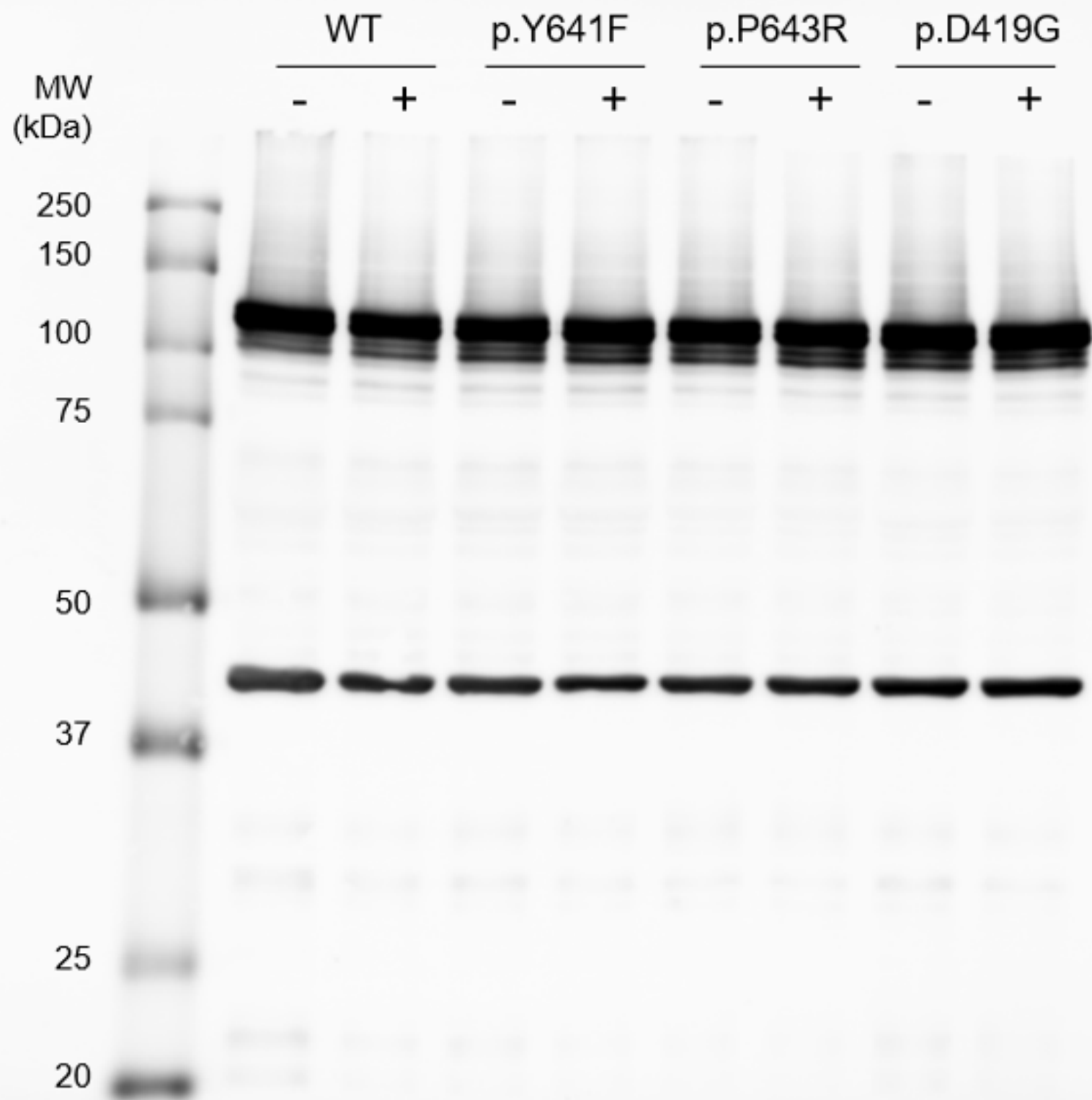

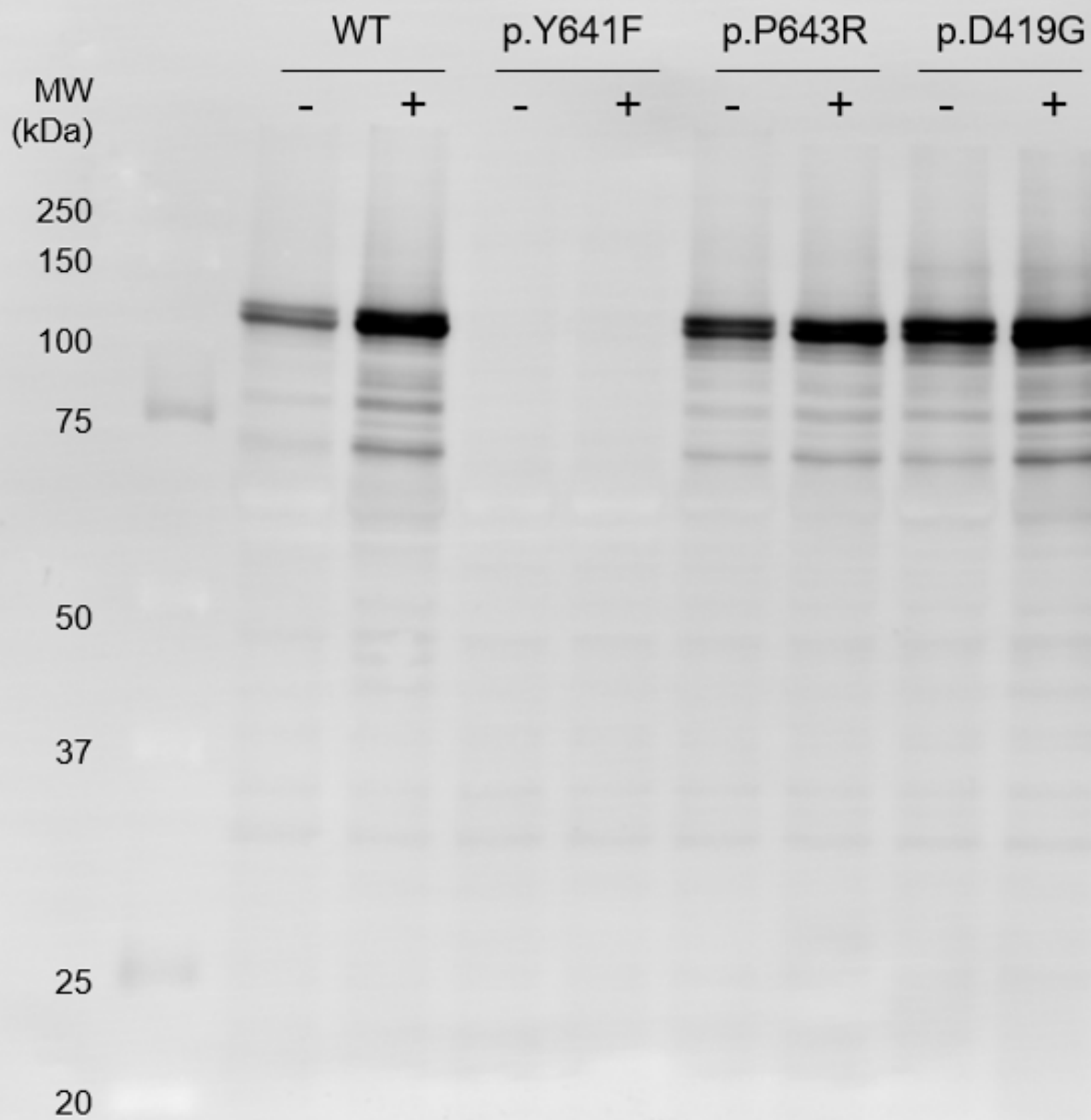

Supplement: SourceData F3 — contains original blots for Fig. 3. [file JEM_20221755_SourceDataF3.pdf]
